# Albumin/Thiacalix[4]arene Nanoparticles as Potential Therapeutic Systems: Role of the Macrocycle for Stabilization of Monomeric Protein and Self-Assembly with Ciprofloxacin

**DOI:** 10.3390/ijms231710040

**Published:** 2022-09-02

**Authors:** Luidmila Yakimova, Aisylu Kunafina, Olga Mostovaya, Pavel Padnya, Timur Mukhametzyanov, Alexandra Voloshina, Konstantin Petrov, Artur Boldyrev, Ivan Stoikov

**Affiliations:** 1 A.M. Butlerov Chemistry Institute, Kazan Federal University, 18 Kremlyovskaya Str., 420008 Kazan, Russia; 2 Arbuzov Institute of Organic and Physical Chemistry, FRC Kazan Scientific Center of RAS, 8 Arbuzov Street, 420088 Kazan, Russia

**Keywords:** sulfobetaines, thiacalixarene, self-assembly, bovine serum albumin, ciprofloxacin

## Abstract

The therapeutic application of serum albumin is determined by the relative content of the monomeric form compared to dimers, tetramers, hexamers, etc. In this paper, we propose and develop an approach to synthesize the *cone* stereoisomer of *p-tert*-butylthiacalix[4]arene with sulfobetaine fragments stabilization of monomeric bovine serum albumin and preventing aggregation. Spectral methods (UV-vis, CD, fluorescent spectroscopy, and dynamic light scattering) established the influence of the synthesized compounds on the content of monomeric and aggregated forms of BSA even without the formation of stable thiacalixarene/protein associates. The effect of thiacalixarenes on the efficiency of protein binding with the antibiotic ciprofloxacin was shown by fluorescence spectroscopy. The binding constant increases in the presence of the macrocycles, likely due to the stabilization of monomeric forms of BSA. Our study clearly shows the potential of this macrocycle design as a platform for the development of the fundamentally new approaches for preventing aggregation.

## 1. Introduction

Serum albumins have a wide range of medical applications [1,2,3,4,5]. They are components of many vaccines [1]. Albumin-based drugs are leading in terms of frequency and volume of transfusions among blood products, and therefore albumin quality indicators are given priority [2]. The main indicator of the albumin quality is the preservation of the native structure during synthesis and storage. Albumin-based drugs with a high content of monomeric albumin have greater therapeutic efficacy, osmotic activity, a long period of circulation in the body and low immunogenicity [3]. Thus, minimizing aggregation is of high priority for the albumin-based drug formulations. Another important area of research related to aggregation is the study of the neurodegenerative diseases such as Parkinson’s disease, Alzheimer’s disease and type II diabetes [4,5], and serum albumins are often used as models in the study of aggregation [6,7,8,9]. Therefore, development of the approaches for aggregation prevention is an extremely important task, where serum albumins can serve as important research object.

Developing compounds which can bind to proteins and prevent aggregation is a promising approach, and calixarenes are often used is a platform [10,11,12,13,14]. Obviously, polyfunctional compounds are most suitable for this purpose, as they can adjust to the protein due to different binding groups. On the other hand, such compounds may tend to aggregate themselves, so a competition between protein binding and self-association must be controlled. We propose using a thiacalix[4]arene as a platform for the synthesis of compounds with a controlled ability to self-assemble. Macrocyclic rings can be relatively easy functionalized [15,16]. Due to the lability of macrocyclic platform, the functional groups are arranged in space in a predetermined fashion and can be fixed in the several conformations (*cone*, *partial cone* and *1,3-alternate*) [15,17]. These arrangements allow binding to the target substrate and determine the tendency to self-assemble into nanostructures [18,19,20]. Modified thiacalix[4]arenes are amphiphilic structures in which polar hydrophilic substituents are spatially separated from the hydrophobic macrocyclic fragment, which leads to the formation of micellar structures in aqueous solutions that can include various biologically significant molecules [21,22,23]. We have already successfully used this type of compounds for protein binding, incl. enzymes [21,24]. By choosing different substituent groups the properties, including biological activity, of calixarenes can be adjusted to a particular application.

Among the substituent groups, the sulfobetaine fragments are of particular interest. Sulfobetaine derivatives simultaneously contain both positively and negatively charged ionic centers but maintain overall charge neutrality. As a rule, they exhibit low toxicity, excellent water solubility, wide isoelectric range, and high stability [25,26,27]. Betaine derivatives are used as components in some drug formulations [28].

Therefore, the aim of this work is the synthesis of thiacalix[4]arene sulfobetaine derivatives in the *cone* conformation and study their interaction with serum albumin with a special attention to the aggregation behavior. As a model, we chose bovine serum albumin (BSA), which is very close in structure to human (HSA) [1]. The potential stabilization of the monomeric form of albumin should potentially enhance its interaction with drugs. As a model drug, we chose a well-known antimicrobial drug in the fluoroquinolone series—ciprofloxacin [29,30]. Having both carboxyl and amine groups in its structure, ciprofloxacin binds to ionic (both cationic and anionic) compounds [31,32]. Ciprofloxacin is one of the most effective antimicrobial agents of the fluoroquinolone group. It is active against a wide range of pathogenic bacteria and has not lost its significance for several decades. It binds well to blood plasma proteins, in particular albumin, and is distributed in body tissues. In the present work we describe the synthesis of the novel sulfobetaine derivatives of thiacalix[4]arene and describe their effect on the aggregation of serum albumin and its interaction with ciprofloxacin.

## 2. Results and Discussion

### 2.1. Synthesis of Tetrasubstituted p-tert-butylthiacalix[4]arene Containing Sulfobetaine Fragments

**Scheme 1:**
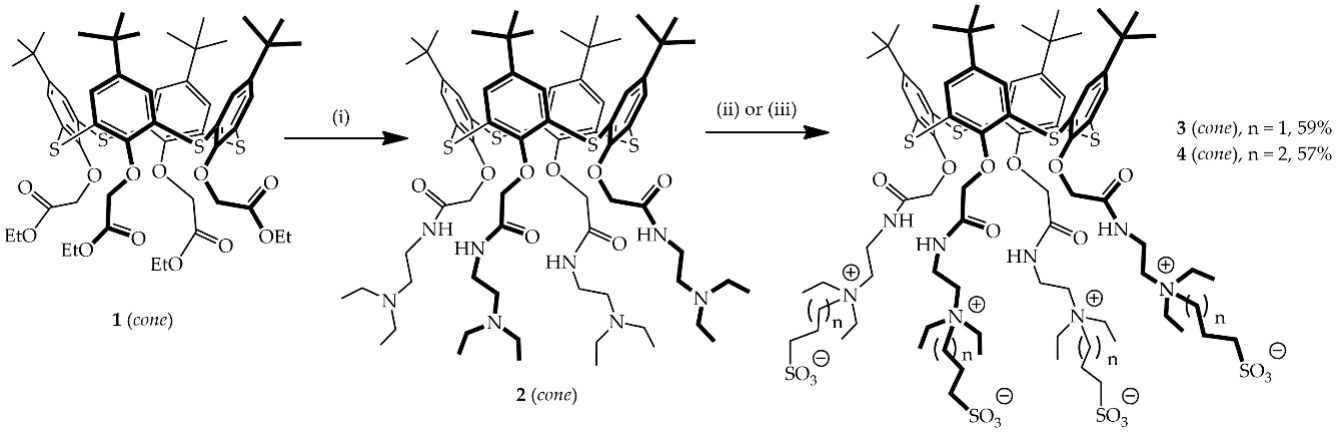
Reagents and conditions: (i)—*N,N*-diethylethane-1,2-diamine, C_6_H_5_CH_3_/CH_3_OH, reflux; (ii)—1,3-propanesultone, CH_3_CN, reflux, 72 h; (iii)—1,4-butanesultone, CH_3_CN, reflux, 240 h.

Earlier, we showed the potential of some sulfobetaine derivatives of thiacalix[4]arene for formation of thiacalix[4]arene/Ag nanoparticles [25,33]. Here, we synthesized new sulfobetaine derivatives of thiacalix[4]arene as perspective macrocycles able to stabilize BSA molecules. The aminolysis of compound **1** with N,N-diethylethane-1,2-diamine resulted in formation of the compounds **2** containing amide and tertiary amine groups at the lower rim (Scheme 1) [34]. Next, compound **2** was involved in the reaction with 1,3-propane- and 1,4-butanesultones to obtain the corresponding sulfobetaine derivatives **3** and **4**. However, reaction with 1,3-propanesultone gave product **3** with a rather low yield of 59%. Ternary amino groups as reaction centers lay in close spatial proximity to the macrocycle platform. Thus, we assumed that this factor eventually prevents the reaction from proceeding further. Earlier, the effect of the alkylidene spacer and the *p-tert*-butylthiacalix[4]arene macrocyclic platform on the reactivity of terminal groups [35,36] was demonstrated.

The failure of macrocycle **2** reaction with 1,4-butanesultone may be explained by the lower reactivity of the latter compared to 1,3-propanesultone [33]. Therefore, modified conditions were tested for this reaction utilizing four-fold excess of 1,4-butanesultone and four-fold increase in reaction time. Under these conditions the product **4** was isolated at 57% yield. The individuality of the products **3** and **4** and the completeness of substitution are confirmed by the presence of a single singlet at 1.06 ppm in their ^1^H NMR spectra due to the resonance of the *tert*-butyl group protons (Figures S1 and S6). Also, there is the significant downfield shift of the proton signals of the methylene fragments bound to the quaternary ammonium nitrogen atom in compounds **3** and **4** compared to the signals in compound **2** in the ^1^H NMR spectra [34]. The complete set of ^1^H, ^13^C NMR and IR spectroscopy and ESI mass spectrometry data confirm the formation of the tetrasubstituted products **3**, **4** (Figures S1–S10).

### 2.2. Cytotoxicity of Test Compounds **3**, **4** on Cancer and Non-Cancer Human Cell Lines

To verify the potential pharmaceutical application of the newly synthesized macrocycles, at the initial stage, we studied the synthesized macrocycles for their cytotoxic effect on normal liver cells (Chang liver) and cancer cell lines (M-HeLa, HuTu 80, MCF-7) in the concentration range of 1–100 µM. This concentration range is recommended for screening for new anticancer agents (1–100 µM) [37].

It was shown that compounds **3**, **4** do not demonstrate cytotoxic activity against all cell lines (Table 1). It opens prospects for the use of new macrocycles in biological media.

The experiments were repeated three times. The results are expressed as the mean ± standard deviation.

### 2.3. Association of Water-Soluble Sulfobetaines **3**, **4** with BSA in Solution and Solid Phase

Macrocycles **3** and **4** have been employed to engineer the albumin/thiacalix[4]arene nanoparticles as potential therapeutic systems, as these macrocycles have been established to be non-toxic. It is known that when macrocycles are localized inside an albumin globule, they trigger sequential monomer addition, with both monomer structures and some larger aggregates present in the solution. If a macrocycle is localized on the surface of a protein, protein aggregation is caused by the formation of associates with two or more globules [4].

Dynamic light scattering (DLS) was used to study the association of BSA in the presence of macrocycles **3** and **4** in aqueous solution (Figures S11–S40). In the absence of protein, the aqueous solutions of macrocycle **3** display high dispersity (PDI = 0.49 ÷ 0.56) and particle size in the range from 170 to 325 nm (Supporting Information, Table S2). However, compound **4** in PBS (pH 7.4) at concentrations of 1 × 10^−5^ and 5 × 10^−6^ M forms a monodisperse system with a particle diameter of 187 nm (PDI = 0.27 ÷ 0.30) (Figures S24 and S25). The protein itself in the studied buffer has a low polydispersity over the entire range of concentrations (1 × 10^−6^–5 × 10^−4^ M) (Figures S35–S40), predominantly being in the monomeric form [38].

**Figure 1:**
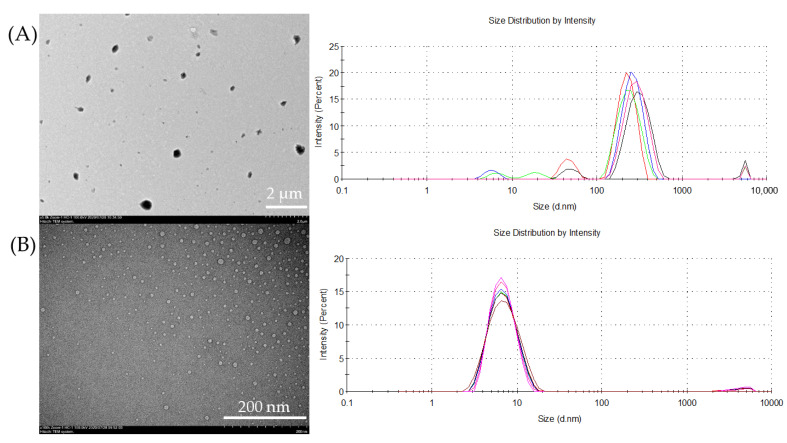
Size analysis of the (**A**) **3** and (**B**) **3**/BSA by TEM (**left**) and DLS (**right**). Colored lines represent multiple experiments.

**Figure 2:**
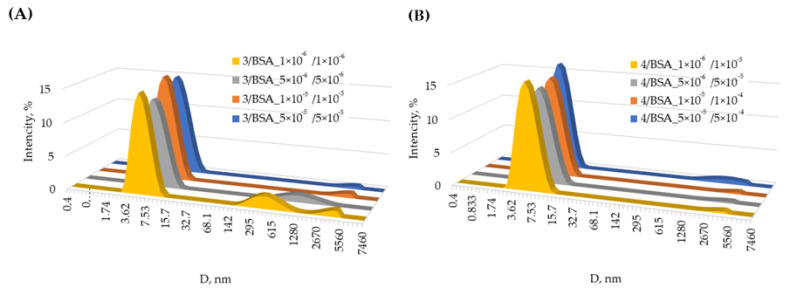
DLS profiles of solutions of BSA in the presence of the **3** (**A**) and **4** (**B**).

Content of BSA monomeric forms in the presence of compounds **3** and **4** changes in different ways. At an equimolar ratio (1/1) of protein and macrocycles, the **3**/BSA is monodisperse at almost any concentration of compound **3** (Figures S15–S22). The most homogeneous system is formed at 50 μM concentration of both components (PDI = 0.15), where the content of particles with a hydrodynamic diameter of 7.2 nm reached 98% (Figure 1B, Table S1). The particle size **3**/BSA determined by the DLS method agrees well with the TEM data (Figure 1B). As shown in Figure 1B, TEM results revealed that **3**/BSA particles are in spherical shapes with homogenous and relatively monodisperse distributions. The TEM image of macrocycle **3** shows aggregates having various shapes and sizes (Figure 1A). With decreasing concentrations, the polydispersity of the systems increases, while the content of monomeric forms decreases to 81% (PDI = 0.26) (Figure 2A, Table S1).

In the case of macrocycle **4**, at all concentrations studied, the equimolar system **4**/BSA is polydisperse (Table S1). The smallest polydispersity is found in the case of the minimum concentrations of components (1 μM) (PDI = 0.28) (Figure S30, Table S1). As the concentrations of the components increase, the PDI value increases to 0.43 (Figure S27, Table S1). In all cases, the content of the monomeric protein form in the presence of macrocycle **4** (72–80%) is lower than in the case of the individual protein, or comparable to it (for a concentration of 1 μM).

At 10-fold excess of BSA to macrocycles (10/1) alternative changes are observed (Figure 2, Table S2). In the presence of macrocycle **3**, a decrease in the content of monomeric forms of the protein is observed in comparison with the individual biopolymer (Figures S19–S22, Table S2). The amount of its monomeric form does not exceed 85% (Figure S22, Table S2). PDI is in the range of 0.30 ÷ 0.38 (Figures S19–S22, Table S2). It should be noted that the most polydisperse system is formed at high concentrations of components (500 µM protein). However, macrocycle **4** has a completely different effect on the aggregation properties of BSA: in its presence, the content of monomeric BSA increases in the entire range of concentrations studied. The content of particles with a diameter of 6–7 nm reaches 99% (Figure 2B and Figures S31–S34, Table S2).

Determination of electrokinetic potentials for several of the most monodisperse systems (with minimum PDI value) (Table 2) showed that all associates are negatively charged in the solution. For system **4**/BSA with a low content of sulfobetaine (molar ratio 1/10), the values of the electrokinetic potentials are higher (−(9.24 ÷ 9.87) mV) (Table 2). With an increase of the sulfobetaine concentration up to the equimolar ratio (system **3**/BSA), the electrokinetic potentials of the systems naturally decrease (−4.91 and −6.86 mV) (Table 2). Thus, it can be assumed that the protein molecules are located on the outer part of the associates formed. However, the small values of electrokinetic potentials in all cases indicate the low stability of the resulting colloidal systems [39].

Thus, we have shown that the addition of macrocycles **3** and **4** has a different effect on the amount of BSA monomers. It depends both on the concentration of the solutions and on the macrocycle/BSA molar ratio. Despite the difference in the behavior of macrocycles **3** and **4**, we found the optimal conditions when the content of the monomeric form predominates in the solution. Taking into account the size of albumin/thiacalix[4]arene nanoparticles based on macrocycle **3** and **4**, one reaches the conclusion that BSA molecule, wrapped around the macrocycle, is realized [40].

It is the difference in one methylene group that can have a significant effect on their self-assembly with BSA. In our previously paper [33] we showed that a sulfobetaine fragment with propylidene linker is cyclized to form a stable six-membered and charge compensation. Whereas in the case of the sulfobetaine fragment with the butylidene linker, no cycle is formed, and no charge compensation occurs in this fragment. A similar picture is obviously observed for macrocycles **3** and **4**, which differ from each other in the length of the alkylidene linker between the ammonium and sulfo groups. This leads to a different contribution of intramolecular forces (electrostatic, van der Waals, hydrophobic, hydrogen bonds, etc.). Compensation of charges in macrocycle **3** will neutralize electrostatic interactions. Van der Waals forces and hydrogen bonding will prevail. In the case of macrocycle **4**, electrostatic interactions will be predominant in accordance with [33]. Therefore, we used a set of methods to study the nature of the interaction between BSA and macrocycles **3** and **4**.

### 2.4. UV-Vis and Fluorescence Spectroscopy of Water-Soluble Sulfobetaines 3 and 4 with BSA

The most convenient method for studying the interaction of biological objects with various compounds is electron absorption spectroscopy in the UV and visible range [41]. However, this method failed to establish interaction of sulfobetaines **3** and **4** with BSA (Figures S41 and S42). BSA itself has an absorption in the region of 200–300 nm, due to the absorption of tyrosine and tryptophan fragments [42]. Macrocyclic compounds **3** and **4** containing aromatic fragments also absorb in the same region. However, when each compound (**3** or **4**) is mixed with BSA at 1/1 molar ratio, there is no significant change in the spectra compared to additively obtained spectra, in the region of 280 nm (Figures S41 and S42). Adding a 10-fold excess of protein solution to macrocycles **3** and **4** revealed significant hyperchromic effects in the spectra (Figures S43 and S44). After half an hour, the optical density of the solution did not change.

**Figure 3:**
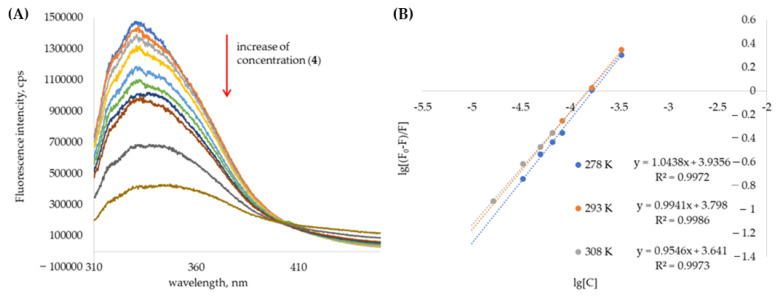
(**A**) Fluorescence spectra of BSA in the presence of **4** at 20 °C; (**B**) the graphs are plotted in the linearization of Stern–Volmer coordinates for **4**/BSA system.

Another sensitive method for studying the interaction (mechanism and thermodynamic parameters) is fluorescence spectroscopy [43]. BSA has an intense intrinsic fluorescence with a maximum at 330 nm (λ_ex_ = 285 nm). The fluorescence of BSA is due to the presence of fluorescently active tryptophan, tyrosine, and phenylalanine fragments in its structure [44]. In the presence of compounds **3** and **4**, a significant quenching of the protein emission is observed with a red shift of the emission maximum (up to 350 nm) (Figure 1A and Figures S45–S47, S51–S53). This fact is apparently due to an increase in the polarity of the environment of tryptophan fragments in the protein when sulfobetaines are introduced into the solution [41]. Therefore, it can be inferred that both macrocycles are located near the Trp residue in BSA molecule [42]. It is known that the BSA molecule contains two different tryptophan residues Trp134 and Trp212, which differ significantly in their environment [45]. The interactions between BSA with **3** and **4** are additionally studied via fluorescence spectroscopy at the excitation wavelength is 295 nm. This provides information about the nature of the binding interaction between a macrocycles and tryptophan residues from the BSA. A significant quenching of the protein emission is also observed with a shift in the emission maximum from 352 to 368 nm [43] (Figures S56 and S57). The obtained data allow us to assume that the binding occurs on the protein surface (Trp134) in the immediate vicinity of the tryptophan fragment. However, the mechanism of this process remains unclear. Two types of quenching are known: static (due to the formation of a non-fluorescent associate) and dynamic (due to collisions with quencher molecules and energy transfer to them) [43], or their combination [46]. To establish the quenching mechanism, experiments were carried out at various temperatures, after which graphs were plotted in the Stern–Volmer coordinates (Figure 3B and Figure S48). In the entire studied range of concentrations of **3** and **4**, the graphs were linear, which indicates a single quenching mechanism. A decrease in the slope of the straight lines with increasing temperature from 278 K to 308 K indicates a static quenching mechanism. It should be noted that in a few publications only dynamic quenching mechanism is noted for BSA in the presence of various binding agents [47,48]. However, in other works a static mechanism is also described [49,50,51].

The association constants (*K_a_*) and binding site (*n*) of macrocycle/BSA associates are calculated using the following formula [52]:

lg[(F_0_ − F)/F] = lg*K_a_* + *n*lg[C] (1)

According to the equation (1), *K_a_* can be calculated from the curve of lg[(F_0_−F)/F] versus lg[C], as shown in (1) (Figure 3 and Figure S49). The calculated results are summarized in Table 3. These results show that, within the temperature range studied, the value *K_a_* is calculated to be approximately 10^2^–10^3^, indicating medium strength interaction between macrocycles and BSA. The value *n* of **3**/BSA and **4**/BSA associates is close to 1, indicating that BSA has a single high affinity binding site for macrocycle. It is also found that as temperature increases, *K_a_* value decreases, suggesting that the stability of **3**/BSA and **4**/BSA associates decreases with the increasing of temperature.

To better understand the binding between BSA and macrocycles **3** and **4**, the van’t Hoff Equation (2) was used to calculate the thermodynamic enthalpy (H_0_) and entropy (S_0_) of **3**/BSA and **4**/BSA associates.

lnK = −ΔH_0_/RT + ΔS_0_/R (2)

As shown in Figures S50 and S55, the curves of ln*K_a_* and 1/T were used to determine the thermodynamic parameters of **3**/BSA and **4**/BSA associates at three different temperatures, i.e., 278 K, 298 K, and 308 K. Once the ΔH_0_ and ΔS_0_ values are determined, the variation in Gibbs free energy (ΔG_0_) can be calculated by the following standard Equation (3).

ΔG_0_ = ΔH_0_ − TΔS_0_ (3)

Values ΔH_0_, ΔS_0_ and ΔG_0_ at three different temperatures are listed in Table 3.

Generally, ΔG reflects the possibility of reaction, while ΔH and ΔS are the main quantities for judging the binding force. From the viewpoint of thermodynamics, ΔH > 0 and ΔS > 0 suggest hydrophobic forces; ΔH < 0 and ΔS < 0 imply van der Waals forces and hydrogen bonds; ΔH < 0 and ΔS > 0 reflect electrostatic forces [52]. In summary, the negative ΔH_0_ and ΔS_0_ values indicate that hydrogen bonding and van der Waals interactions play a major role in the interaction of the **3** into the BSA. The negative ΔH_0_ and positive ΔS_0_ values indicate that electrostatic forces play a major role in the interaction of the **4** into the BSA. Thermodynamic parameters and nature of the binding forces confirmed our assumption about the significant effect of the length of the alkylidene linker between ammonium and sulfo groups in macrocycles **3** and **4** on the interaction with the protein.

### 2.5. Circular Dichroism Spectroscopy of Water-Soluble Sulfobetaines **3** and **4** with BSA and Molecular Docking of Their Associates

**Figure 4:**
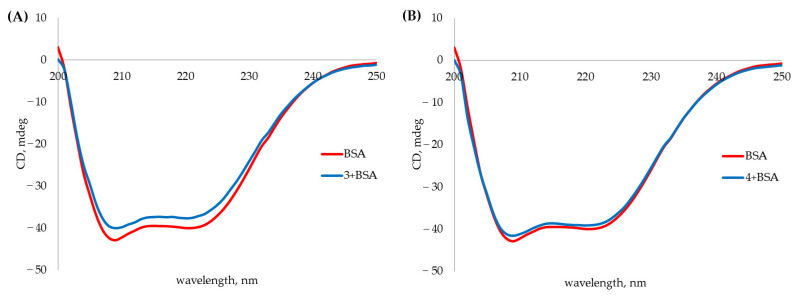
Circular dichroism spectra of (**A**) BSA (3.3 × 10^−7^ M) and the associate of **3** (3.3 × 10^−6^ M) with BSA (3.3 × 10^−7^ M) in buffer at pH 7.4; (**B**) BSA (3.3 × 10^−7^ M) and the associate of **4** (3.3 × 10^−6^ M) with BSA (3.3 × 10^−7^ M) in buffer at pH 7.4.

To assess the effect of the interaction of BSA with thiacalixarenes on the state of the protein structure, the circular dichroism method was used. Circular dichroism spectroscopy is a sensitive method for monitoring the conformational state of biopolymers such as proteins and DNA. The CD spectrum of BSA has two negative signals with maxima at 208 nm (π–π* transition) and 222 (n–π* transition) nm (Figure 4), which corresponds to the mostly α-helical protein conformation whose content can be estimated by [53]:

α-helix (%) = [(−MRE_208_ − 4000)/(33,000 − 4000)] × 100, (4)

where MRE_208_ is the MRE value observed at 208 nm, 4000 is the MRE value of the β shape and random coil conformation at 208 nm, and 33,000 is the MRE value of the pure α-helix at 208 nm. The MRE_208_ value used to indicate the change in secondary structure of BSA determined by [53]:

MRE_208_ = CD (mdeg)_208_/(10 × n × l × C_p_), (5)

where n is the number of amino acid residues (583 for BSA), l is the cell path length and C_p_ is the molar concentration of BSA. The results demonstrate that the secondary structure of BSA has a partial change from α-helical content. The addition of a 10-fold excess of sulfobetaines **3** and **4** leads only to a slight change in the secondary protein structure, as evidenced by a change in the amplitude of the signals. For compound **4**, it turns out to be minimal (Figure 4). In both cases, a slight hypochromia is observed, which indicates a slight decrease in the proportion of the α-helix in the packing of the biopolymer molecules [53]. The α-helical content decreased from 62.02% of free BSA to 57.12% (**3**/BSA) and 60.14% (**4**/BSA). The changes in case of macrocycle **3** is more significant. We believe that this is due to the different structure of macrocycles **3** and **4** and different types of intermolecular interactions that arise during the association of BSA with macrocycles **3** and **4** according to fluorescence data. Based on these analyses, we conclude that the addition of macrocycles **3** and **4** slightly alters the secondary structure of BSA, resulting in a decrease in α-helical content, which is nonetheless still dominant in the secondary structure. We believe that changes in the secondary structure of the protein may be associated with a change in the polarity of the medium caused by the addition of sulfobetaines **3** and **4**. Such behavior of proteins is well known [54,55]. Furthermore, this rationalizes the fact that there are different modes of interaction in the two different systems (electrostatic, van der Waals, and hydrogen bonds). It is in good agreement with the results of other spectral methods discussed above.

**Figure 5:**
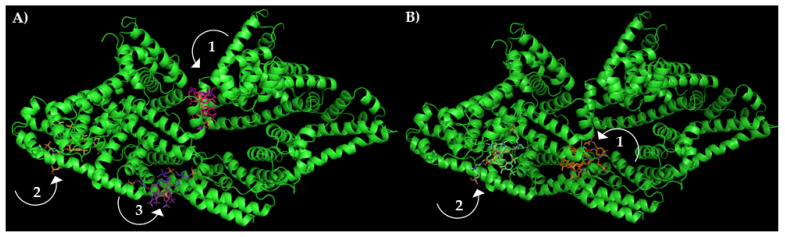
Potential sites for binding macrocycle **3** (**A**) and **4** (**B**) in BSA molecules calculated by DINC 2.0 web server.

Thus, it was shown that thiacalix[4]arenes **3** and **4** interact with BSA without significantly affecting the native structure of the protein. In this regard, we further analyzed the possible protein binding sites with ligands **3** and **4** using the molecular docking method. Molecular docking is a standard computational approach to predict binding modes of protein-ligand associates by exploring different orientations and conformations of the ligand. DINC 2.0 web server [56] was used to determine potential sites of the BSA molecule for binding the macrocycles studied. DINC is a parallelized meta-docking method for the incremental docking of ligands. The strategy of DINC involves incrementally docking the overlapping fragments with a growing number of atoms, while maintaining the number of the flexible bonds constant during this incremental process [57]. The grid size was set to ca. 150 Å × 100 Å × 150 Å xyz points and grid center was designated at protein center (64.2, 25.8, 32.1). Binding energies (kcal/mol) in the DINC 2.0 server output were −5.90, −5.70, −5.60 and −5.50, −5.30 in case of **3** and **4**, respectively. The obtained docking results exhibited relatively high potential for binding of these ligands with BSA (Figure 5). The binding site was located deep inside the protein structure (sites 1, 2, 3). Figure 5 shows the preferred binding sites for macrocycles **3** and **4** in the BSA structure. Here, note that the calculated combined Gibbs free energy is quite close with our experimental result ΔG = −18.2 kJ/mol (4.4 kcal/mol) for **3** and ΔG = −21.4 kJ/mol (5.1 kcal/mol) for **4** at 298 K. This fact additionally confirms the possibility of the formation of associates and the direct effect of macrocycles on the protein monomer content.

### 2.6. Effect of Water-Soluble Sulfobetaines 3 and 4 on the Efficiency BSA/Ciprofloxacin Interactions

**Figure 6:**
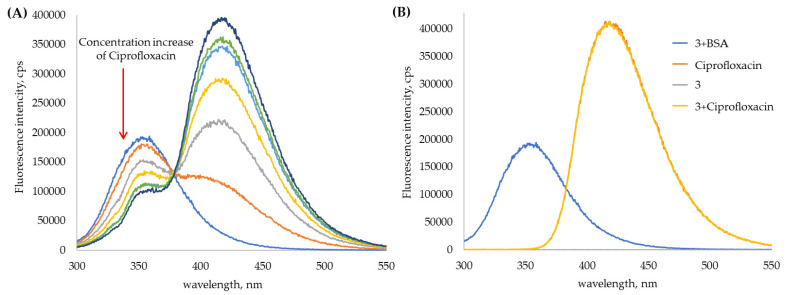
Fluorescence spectra of (**A**) titration of **3** (1 × 10^−5^ M)/BSA (1 × 10^−5^ M) with ciprofloxacin (antibiotic concentration changed from 0 to 5 × 10^−5^ M) (**B**) **3** (1 × 10^−5^ M), ciprofloxacin (4.17 × 10^−5^ M), **3** + BSA system (1/1), **3** + ciprofloxacin system (1/1).

To reveal the effect of macrocycles **3** and **4** on the binding properties of BSA, we studied the interaction of BSA with ciprofloxacin in the presence of these macrocycles. Using the fluorescence spectroscopy, we assessed the efficiency of drug binding to protein in the presence and in the absence of macrocycles. The antibiotic itself fluoresces intensely, having an emission maximum at 415 nm when irradiated with light with a wavelength of 285 nm (Figure 6A). Under these conditions, the macrocycles themselves do not have fluorescence. In the presence of macrocycles **3** and **4**, the intensity of the antibiotic emission does not change (Figure 6B and Figure S58), indicating a lack of the association of ciprofloxacin with thiacalixarenes. At the same time, there is a clear quenching of the BSA emission at 350 nm in the presence of the antibiotic (Figure 6A and Figures S57, S61, S65, S69 and S73).

Based on the results obtained during study of the association of BSA with macrocycles **3** and **4**, we chose the systems **3**/BSA and **4**/BSA in ratios of 1/1 and 1/10. The concentrations of the protein were 10 and 100 μM, respectively. The choice of such systems is due to the increased content of the monomeric protein forms. As shown above, monomeric forms predominate in the case of the **3**/BSA system at a molar ratio of 1/1, and for **4**/BSA, at 1/10 molar ratio. With the addition of the increasing concentrations of ciprofloxacin, a decrease in the intensity of protein emission in the region of 350 nm is observed with an increase in emission at 415 nm, due to an increase in the concentration of the antibiotic (Figure 6A). It should be noted that the antibiotic practically does not fluoresce at the protein emission maximum, which makes it possible to study their interaction at this wavelength and determine the binding constant. Due to the presence of the intense, intrinsic fluorescence of ciprofloxacin the region of emission registration was limited to the range of 300–370 nm for the titration the protein and its associates with macrocycles. The association constants of the protein and its macrocycle associates with the antibiotic were calculated fitting the binding isotherms with a 1:1 binding model [58,59]. The Bindfit application, which was developed for supramolecular systems (Bindfit v0.5 (Open Data Fit, 2016); http://supramolecular. org/bindfit/ (accessed on 1 January 2016)), was used to perform the fit. To confirm the proposed stoichiometry, the titration data were also processed by the binding model at the host:guest ratio = 1:2 and 2:1 (Figures S59–S78). However, in these cases the constants were determined with a much greater uncertainty. The value of the association constant of BSA with ciprofloxacin in the absence of compounds **3** and **4** was 2760 M^−1^ (Table 4).

For **3**/BSA/ciprofloxacin associate (**3**/BSA, 1/1 molar ratio, monodispersed system), the association constant turned out to be somewhat higher (3020 M^−1^). However, at a ratio of 1/10, at which the content of the monomeric form of albumin is reduced, the interaction efficiency decreases (K_a_ = 2710 M^−1^). Interestingly, for the **4**/BSA system in the case of a polydisperse system (ratio 1/1), the interaction efficiency with ciprofloxacin is the lowest (K_a_ = 1220 M^−1^), while for a monodisperse system with a predominance of the monomeric form (ratio 1/10) the efficiency of interaction increases (K_a_ = 2010 M^−1^). Thus, the higher the content of the monomeric form of BSA is, the higher the binding constant of ciprofloxacin by the macrocycle/BSA system is. It is worth noting that the stabilization of the BSA monomer by thiacalix[4]arene with a propylidene linker resulted in more efficient binding of ciprofloxacin than BSA itself.

## 3. Materials and Methods

### 3.1. General

^1^H, ^13^C and 2D ^1^H-^1^H NOESY NMR spectra were recorded on the Bruker Avance-400 (400, 100 MHz respectively) spectrometer (Bruker Corp., Billerica, MA, USA) (^13^C{^1^H} 100 MHz and ^1^H 400 MHz). Chemical shifts were determined against the signals of residual protons of deuterated solvent (DMSO-*d_6_*). The concentration of the sample solutions was equal to 3–5%. Attenuated total internal reflectance IR spectra were recorded with Spectrum 400 Fourier spectrometer (Perkin Elmer Inc, Waltham, MA, USA). Elemental analysis was performed with Perkin Elmer 2400 Series II instrument (Perkin Elmer, Waltham, MA, USA). Electrospray ionization mass spectra (ESI) were obtained on an AmazonX mass spectrometer (Bruker Daltonik GmbH, Bremen, Germany). The measurements were carried out in the positive ions registration regime in the *m*/*z* range from 100 to 2800. The voltage on the capillary was 4500 V. Nitrogen was used as the drying gas with a temperature of 300 °C and a flow rate of 10 L min^−1^. The compounds were dissolved in water to a concentration of 10^−6^ g L^−1^. Data were processed using DataAnalysis 4.0 (Bruker Daltonik GmbH, Bremen, Germany). Melting points were determined using the Boetius Block apparatus (VEB Kombinat Nagema, Radebeul, Germany). Most chemicals were purchased from Acros (Fair Lawn, NJ, USA) and used as received without additional purification.

Compounds **1**, **2** were synthesized by literature method [60,61].

**General procedure for the synthesis of compounds 3 and 4**

The mixture of 5,11,17,23-tetra-*tert*-butyl-25,26,27,28-tetrakis[(N-(3′,3′-diethylaminoethyl)-carbamoylmethoxy]-2,8,14,20-tetrathiacalix[4]arene (*cone*) **(2)** (0.20 g, 0.16 mmol) and 1,3-propanesultone (0.64 mmol) (for compound **3**) or 1,4-butanesultone (2.56 mmol) (for compound **4**) in 15 mL of solvent (CH_3_CN) was refluxed for 72 h (compound **3**) and 240 h (compound **4**). After cooling, the precipitate formed was filtered off. Filtrate was washed with CH_3_CN. The resulting precipitates were dried in vacuo over P_2_O_5_.

**5,11,17,23-Tetra-*tert*-butyl-25,26,27,28-tetrakis[(N-(3′,3′-diethyl-3′-{3”-sulfonatopropyl})ammoniumethyl)-carbamoylmethoxy]-2,8,14,20-tetrathiacalix[4]arene** (*cone*) **(3)**. Yield 0.16 g (59%), Mp: 224 °C. ^1^H NMR (DMSO-*d_6_*, δ, ppm, *J*/Hz): 1.06 (s, 36H, (CH_3_)_3_C), 1.22 (s, 24H, N^+^CH_2_CH_3_), 1.97 (m, 8H, -N^+^CH_2_CH_2_CH_2_SO_3_^−^), 2.05 (m, 8H, -N^+^CH_2_CH_2_CH_2_SO_3_^−^), 2.58 (m, 8H, -N^+^CH_2_CH_2_CH_2_SO_3_^−^), 3.30 (m, 8H, -NCH_2_CH_2_NH), 3.61 (m, 8H, NCH_2_CH_2_NH), 4.85 (s, 8H, OCH_2_CO), 7.4 (s, 8H, ArH), 8.74 (br. s, 4H, CONH). ^13^C NMR (DMSO-*d_6_*, δ, ppm) 169.39, 157.93, 147.32, 135.14, 128.57, 74.66, 56.47, 54.86, 53.46, 47.80, 47.09, 39.94, 34.42, 32.47, 31.18, 18.52, 7.53. FTIR ATR (ν, cm^−1^): 1665 (C=O), 2958 (N^+^), 3320 (NH). MS (ESI): calculated 918.2 [M+2H]^2+^, found: 918.4 [M+2H]^2+^. El. Anal. Calcd for C_84_H_136_N_8_O_20_S_8_ (%): C 55.00%, H 7.47%, N 6.11%, S 13.98%. Found (%): C 55.15%, H 7.41%, N 6.21%, S 13.89%.

**5,11,17,23-Tetra-*tert*-butyl-25,26,27,28-tetrakis[(N-(3′,3′-diethyl-3′-{3”-sulfonatobutyl})ammoniumethyl)-carbamoylmethoxy]-2,8,14,20-tetrathiacalix[4]arene (*cone*) (4).** Yield 0.16 g (57%), Mp: 215 °C. ^1^H NMR (DMSO-*d_6_*, δ, ppm, *J*/Hz): 1.06 (s, 36H, (CH_3_)_3_C), 1.19 (s, 24H, N^+^CH_2_CH_3_), 1.65 (m, 8H, -N^+^CH_2_CH_2_CH_2_CH_2_SO_3_^−^), 1.77 (m, 8H, -N^+^CH_2_CH_2_CH_2_CH_2_SO_3_^−^), 2.54 (m, 8H, -N^+^CH_2_CH_2_CH_2_CH_2_SO_3_^−^), 3.31 (m, 8H, -NCH_2_CH_2_NH), 3.33 (m, 8H, -N^+^CH_2_CH_2_CH_2_CH_2_SO_3_^−^), 3.60 (m, 8H, NCH_2_CH_2_NH), 4.88 (s, 8H, OCH_2_CO), 7.39(s, 8H, ArH), 8.79 (br. s, 4H, CONH). ^13^C NMR (DMSO-*d_6_*, δ, ppm) 169.44, 157.95, 147.18, 135.00, 128.59, 74.89, 74.12, 57.20, 55.12, 53.43, 50.63, 39.93, 34.40, 32.65, 31.18, 22.67, 20.41, 7.61. FTIR ATR (ν, cm^−1^): 1673 (C=O), 2961 (N^+^), 3316 (NH). MS (ESI): calculated 945.8 [M+2H]^2+^, found: 945.9 [M+2H]^2+^. El. Anal. Calcd for C_88_H_143_N_8_O_20_S_8_ (%): C 55.94%, H 7.63%, N 5.93%, S 13.57%. Found (%): C 55.92%, H 7.47%, N 5.97%, S 13.79%.

### 3.2. UV-Visible Spectroscopy

UV-visible spectra were recorded on the Shimadzu UV-3600 spectrophotometer using a 1 cm quartz cuvette at 25 °C. BSA was used as received. For 1/1 molar ratio: the 1 × 10^−4^ M solution of the BSA (300 μL) in phosphate buffer (PBS) pH = 7.4 (0.01 M phosphate buffer, 0.0027 M potassium chloride and 0.137 M sodium chloride, pH 7.4) was added to 300 μL of the solution of host (macrocycle **3** or **4**) (1 × 10^−4^ M) in phosphate buffer (pH = 7.4) and diluted to final volume of 3 mL with phosphate buffer. For 1/10 molar ratio: the 5 × 10^−4^ M solution of the BSA (300 μL) in PBS was added to 300 μL of the solution of host (macrocycle **3** or **4**) (5 × 10^−5^ M) in PBS and diluted to final volume of 3 mL with phosphate buffer. Then UV spectra of the solutions were recorded after 1 h mixing.

### 3.3. Dynamic Light Scattering (DLS)

#### 3.3.1. Particles’ Size

The particles size was determined by the Zetasizer Nano ZS instrument (Worcestershire, UK) at 20 °C. The instrument contains 4 mW He-Ne laser operating at a wavelength of 633 nm and incorporated noninvasive backscatter optics (NIBS). The measurements were performed at the detection angle of 173° and the software automatically determined the measurement position within the quartz cuvette. The experiments were carried out for each solution in triplicate. Synthesized *p-tert*-butylthiacalix[4]arenes **3** or **4** were dissolved completely in PBS buffer (0.01 M phosphate buffer, 0.0027 M potassium chloride and 0.137 M sodium chloride, pH 7.4) at concentrations used in research (from 1 × 10^−6^ M to 5 × 10^−5^ M). BSA concentration was from 1 × 10^−6^ M to 5 × 10^−4^ M.

#### 3.3.2. Zeta Potentials

Zeta (ζ) potentials were measured on a Zetasizer Nano ZS from Malvern Instruments (Worcestershire, UK). Samples were prepared as for the DLS measurements and were transferred with the syringe to the disposable folded capillary cell for measurement. The zeta potentials were measured using the Malvern M3-PALS method and averaged from three measurements.

### 3.4. Transmission Electron Microscopy (TEM)

TEM measurements were made at the Interdisciplinary Center for Analytical Microscopy of the Kazan Federal University. Analysis of samples was carried out using a Hitachi HT7700 Exalens transmission electron microscope (Tokyo, Japan) with an Oxford Instruments X-Maxn 80T EDS detector working in STEM mode. Samples of macrocycles **3**, **4**, BSA and their associates were prepared similarly to those studied by the DLS method. 10 μL of the suspension was placed on a carbon-coated 3 mm copper grid and dried at room temperature using special holder for microanalysis. Analysis was held at the accelerating voltage of 80 kV in STEM mode using Oxford Instruments X-Max^n^ 80T EDS detector.

### 3.5. Fluorescence Spectroscopy

Fluorescence spectra were recorded on the Fluorolog 3 luminescent spectrometer (Horiba Jobin Yvon, Longjumeau, France). The excitation wavelengths were selected as 285 nm and 295 nm. For **3**/BSA and **4**/BSA systems, the emission scan range was 310–450 nm. Excitation and emission slits were 3 nm. Quartz cuvettes with an optical path length of 10 mm were used. Cuvette was placed at the front face position to avoid the inner filter effect. Fluorescence spectra were automatically corrected by the Fluorescence program. Spectra were recorded at 278, 293 and 308 K in PBS buffer (0.01 M phosphate buffer, 0.0027 M potassium chloride and 0.137 M sodium chloride, pH 7.4). Concentration of the BSA was 10 µM. Concentrations of the compounds **3** and **4** were varied from 1 × 10^−4^ M to 1 × 10^−3^ M.

When determining the interaction constants of associates **3**/BSA and **4**/BSA (macrocycle/protein molar ratios were chosen 1/1 and 1/10) with ciprofloxacin, the excitation wavelength was selected as 285 nm. The emission scan range was 300–550 nm when studying the spectral properties of a protein in the presence of ciprofloxacin. During the titration of the **3**/BSA and **4**/BSA associates with the antibiotic, scanning of emission wavelengths was limited to the range of 300–370 nm. Excitation and emission slits were 2 nm. Quartz cuvettes with an optical path length of 10 mm were used. Cuvette was placed at the front face position to avoid the inner filter effect. Fluorescence spectra were automatically corrected by the Fluorescence program. Spectra were recorded at 298 K in PBS. Concentration of the BSA was 10 or 100 µM. Concentrations of the compounds **3** and **4** were 10 µM. Concentration of the Ciprofloxacin was 4.17 × 10^−5^ M and its concentration during titration were varied from 3.33 × 10^−5^ M to 4 × 10^−4^ M.

### 3.6. Circular Dichroism (CD) Studies

Changes in the intensity of the CD signal of the BSA, alone and in the presence of the compounds **3** and **4**, were recorded from 200 nm to 250 nm at 293 K using the Jasco J-1500 spectropolarimeter (Easton, MA, USA) in the quartz cuvette with the 10 mm optical path length. Experiment was carried out in PBS buffer (0.01 M phosphate buffer, 0.0027 M potassium chloride and 0.137 M sodium chloride, pH 7.4). BSA concentration was 3.3 × 10^−7^ M. The concentration of compounds **3** and **4** was 3.3 × 10^−6^ M.

### 3.7. Cytotoxicity of Test Compounds on Cancer and Normal Human Cell Lines

The M−HeLa clone 11 human, epithelioid cervical carcinoma, strain of HeLa, clone of M—Hela; human duodenal adenocarcinoma (HuTu 80); human breast adenocarcinoma cells (MCF-7) from the Type Culture Collection of the Institute of Cytology (Sankt-Petersburg) and human Chang liver from the collection and Research Institute of Virology, Russian Academy of Medical Sciences (Moscow) were used in the experiments.

Cytotoxic effects of the test compounds on human cancer and normal cells were estimated by means of the multifunctional Cytell Cell Imaging system (GE Health Care Life Science, Sweden) using the Cell Viability Bio App which precisely counts the number of cells and evaluates their viability from fluorescence intensity data [60]. The cells were cultured in a standard Eagle’s nutrient medium manufactured at the Chumakov Institute of Poliomyelitis and Virus Encephalitis (PanEco company) and supplemented with 10% fetal calf serum and 1% nonessential amino acids.

The cells were plated into a 96-well plate (Eppendorf) at a concentration of 1 × 10^5^ cells/mL, 150 μL of medium per well, and cultured in a CO_2_ incubator at 37 °C. 24 h after seeding the cells into wells, the compound under study was added at a preset dilution, 150 μL to each well. The dilutions of the compounds were prepared immediately in nutrient media. The experiments were repeated three times. Intact cells cultured in parallel with experimental cells were used as a control. The cytotoxic effect of the test compounds was determined at concentrations of 1–100 μM. The calculation of IC_50_, the concentration of the drug causing inhibition of cell growth by 50%, was performed using the program MLA—Quest Graph™ IC50 Calculator (AAT Bioquest, Inc., Sunnyvale, CA, USA, https://www.aatbio.com/tools/ic50-calculator (accessed on 25 July 2019)).

### 3.8. Molecular Docking

DINC 2.0 web server [56,57] was used to study molecular interactions between macrocycles **3** and **4** with BSA. DINC is a parallelized meta-docking method for the incremental docking of ligands. The strategy of DINC involves incrementally docking overlapping fragments with a growing number of atoms, while maintaining the number of flexible bonds constant during this incremental process [57]. The grid size was set to ~30 Å × 30 Å × 30 Å xyz points and grid center was designated at dimensions (x, y, and z): 0.8, 0.8, and −6.4.

## 4. Conclusions

In summary, new water-soluble *p-tert*-butylthiacalix[4]arene derivatives with a propylidene and butylidene linker in sulfobetaine fragment in the *cone* conformation were synthesized. Synthesized macrocycles do not demonstrate cytotoxic activity against normal liver cells (Chang liver) and cancer cell lines (M-HeLa, HuTu 80, MCF-7). It opens up prospects for the use of new macrocycles in biological media. We have constructed non-toxic supra-biomolecular structures based on self-assembly involving BSA and these macrocycles. Various spectral methods (UV-Vis, CD, fluorescence spectroscopy, DLS) have shown that these derivatives are able to stabilize monomeric forms of BSA with hydrodynamic diameter about 7 nm at different conditions. It is the difference in one methylene group in sulfobetaine fragment that can have a significant effect on their self-assembly with BSA. This led to a different contribution of intramolecular forces (electrostatic, van der Waals, and hydrogen bonds) in the associate formation. Results for spectroscopic studies suggest that thiacalix[4]arene with propylidene linker could bind to BSA using hydrogen bonding and van der Waals forces. The negative ΔH0 and positive ΔS0 values indicate that electrostatic forces play a major role in the interaction of the thiacalix[4]arene with butylidene linker into the BSA. The negative values of ΔG in both cases indicate that the interaction process was spontaneous. Molecular docking with DINC 2.0 web server determined several potential binding sites with negative binding energy and confirmed the experimental data on the interaction of macrocycles studied with BSA. We evaluated the effect of the content of BSA monomers in the systems macrocycle/BSA on the efficiency of ciprofloxacin binding. The assemblies successfully uptakes the antibiotic drug, ciprofloxacin. It was shown, the higher the content of the monomeric form of BSA is, the higher the binding constant of ciprofloxacin by the macrocycle/BSA system is. It is worth noting that the stabilization of the BSA monomer by thiacalix[4]arene with a propylidene linker resulted in more efficient binding of ciprofloxacin than BSA itself. Such host-assisted protein assemblies are promising for stabilizing/protecting the protein structure in the most effective therapeutic monomer form and a useful method to inhibit protein misfolding and aggregate, responsible for several neurodegenerative diseases. We hope that the results of our work will make it possible to develop fundamentally new approaches to the stabilization of native forms of proteins. The use of these approaches will help to increase the effectiveness of drugs.

## Figures and Tables

**Table 1:** In vitro cytotoxic effects (µM) of sulfobetaine derivatives **3**, **4**.

Test Compounds	IC_50_ (µM)			
	M-HeLa	HuTu 80	MCF-7	Chang Liver
**3**	500	500	500	500
**4**	>500	>500	>500	>500

**Table 2:** Electrokinetic potentials (ζ) of the aggregates, obtained for BSA in the presence of the compounds **3** and **4** in PBS (pH = 7.4) at 20 °C, and polydispersity index (PDI).

System	C, M	ζ, mV	PDI
**3**/BSA	5 × 10^−5^/5 × 10^−5^	−6.86 ± 0.92	0.15 ± 0.02
**3**/BSA	1 × 10^−5^/1 × 10^−5^	−4.91 ± 0.27	0.22 ± 0.04
**4**/BSA	5 × 10^−5^/5 × 10^−4^	−9.74 ± 1.71	0.22 ± 0.03
**4**/BSA	1 × 10^−5^/1 × 10^−4^	−9.24 ± 1.14	0.15 ± 0.02
**4**/BSA	5 × 10^−6^/5 × 10^−5^	−9.87 ± 2.62	0.15 ± 0.01

**Table 3:** Calculated parameters of **3**/BSA and **4**/BSA associates at different testing temperatures (278, 298, 308 K) at PBS (pH 7.4).

System	T, K	lg*K_a_*	ΔH_0_, KJ mol^−1^	ΔS_0_, J mol^−1^ R^−1^	ΔG_0_, KJ mol^−1^
**3**/BSA	278	3.72	−40.9	−76.9	−19.5
	293	3.16			−18.0
	308	2.98			−17.2
**4**/BSA	278	3.94	−16.3	16.7	−21.0
	293	3.80			−21.3
	308	3.64			−21.5

**Table 4:** Binding constants of ciprofloxacin with BSA in the absence and presence of **3** and **4**.

Macrocycle	C_BSA_, μM	Macrocycle/BSA Molar Ratio	K_a_ [M^−1^] (% Error)
-	10	0/1	2760 (3.7)
**3**	10	1/1	3020 (2.5)
	100	1/10	2710 (2.2)
**4**	10	1/1	1220 (3.9)
	100	1/10	2010 (2.4)

## Data Availability

The data presented in this study are available in Supplementary Materials.

## Supplementary Materials

The following supporting information can be downloaded at: https://www.mdpi.com/article/10.3390/ijms231710040/s1.
