# Supplementary material for: Albumin/Thiacalix[4]arene Nanoparticles as Potential Therapeutic Systems: Role of the Macrocycle for Stabilization of Monomeric Protein and Self-Assembly with Ciprofloxacin
Source: Int J Mol Sci. 2022 Sep 2;23(17):10040. doi: 10.3390/ijms231710040 (PMC9455997; doi:10.3390/ijms231710040)
Supplement: Supplementary file 1 [file ijms-23-10040-s001.zip › ijms-1865990-supplementary.pdf]

Albumin/thiacalix[4]arene nanoparticles as potential therapeutic systems: role of the macrocycle for stabilization of monomeric protein and self-assembly with ciprofloxacin

Luidmila Yakimova [1,*], Aisylu Kunafina [1], Olga Mostovaya [1], Pavel Padnya [1], Timur Mukhametzyanov [1], Alexandra Voloshina [2], Konstantin Petrov [2], Artur Boldyrev [1], and Ivan Stoikov [1,*]

[1]A.M. Butlerov Chemical Institute, Kazan Federal University, 420008 Kazan, Kremlevskaya 18, Russian Federation

[2]Arbuzov Institute of Organic and Physical Chemistry, FRC Kazan Scientific Center of RAS, Arbuzov str. 8, Kazan, 420088, Russian Federation

e-mail: mila.yakimova@mail.ru, ivan.stoikov@mail.ru

Electronic Supplementary Information (42 pages)

1. NMR, ESI, IR spectra of compounds **3**-**4**.

2. Dynamic light scattering

3. UV spectra

4. Fluorescence

5. Tables

1. NMR, ESI, IR spectra of compounds 3, 4

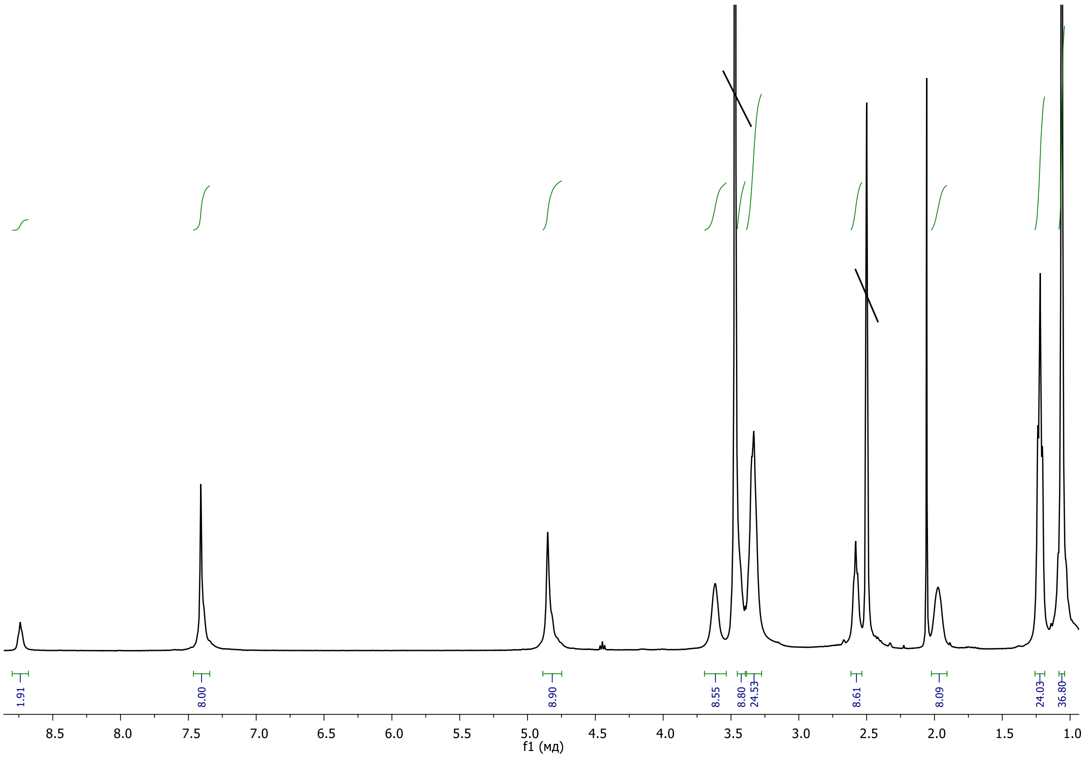

Figure S1. ^{1}H NMR spectrum of 5,11,17,23-Tetra-*tert*-butyl-25,26,27,28-tetrakis[(N-(3',3'-diethyl-3'-{3"-sulfonatopropyl})ammoniumethyl)-carbamoylmethoxy]-2,8,14,20-tetrathiacalix[4]arene (*cone*) **(3)**, DMSO-d_6, 298 K, 400 MHz.

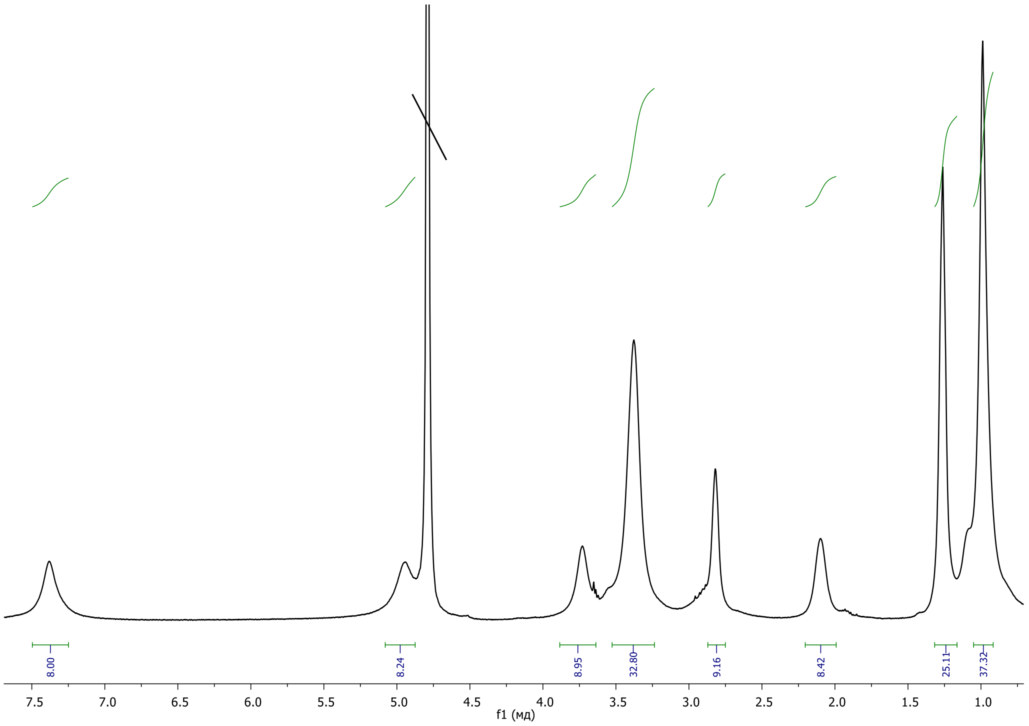

Figure S2. ^{1}H NMR spectrum of 5,11,17,23-Tetra-*tert*-butyl-25,26,27,28-tetrakis[(N-(3',3'-diethyl-3'-{3"-sulfonatopropyl})ammoniumethyl)-carbamoylmethoxy]-2,8,14,20-tetrathiacalix[4]arene (*cone*) **(3)**, D_2O, 298 K, 400 MHz.

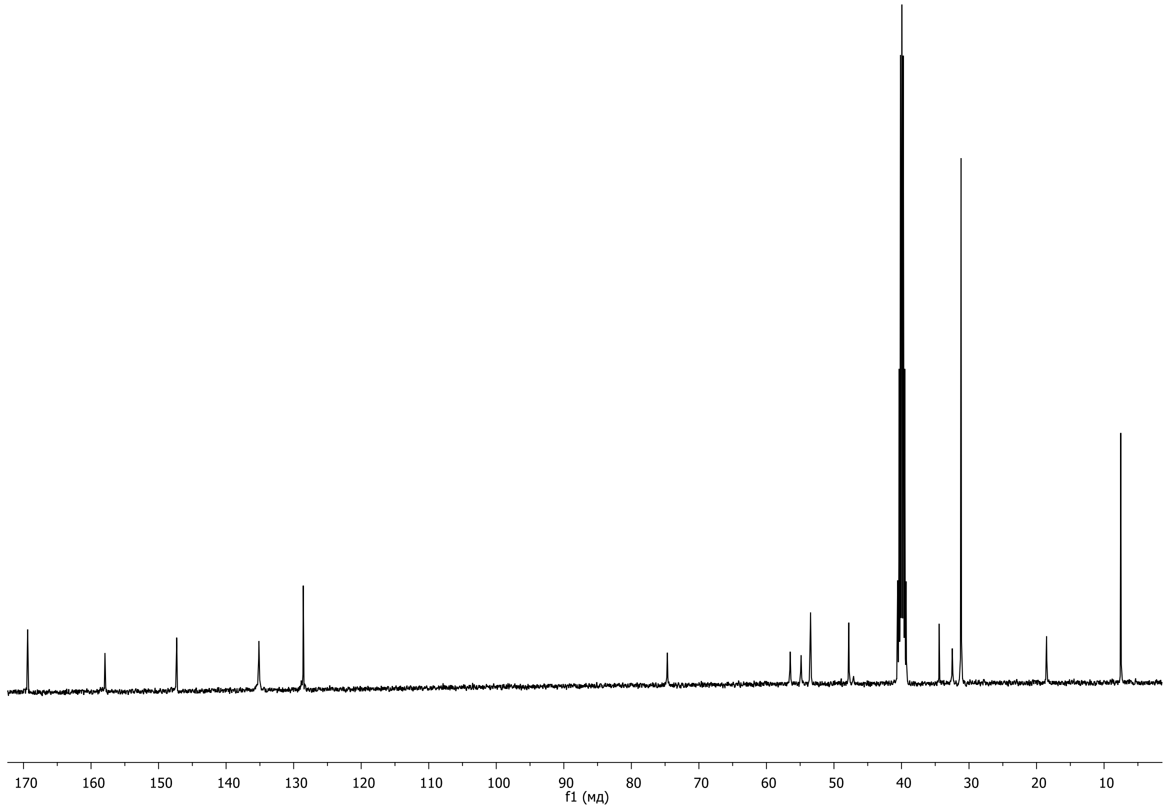

Figure S3. ^{13}C NMR spectrum of 5,11,17,23-Tetra-*tert*-butyl-25,26,27,28-tetrakis[(N-(3’,3’-diethyl-3’-{3”-sulfonatopropyl})ammoniumethyl)-carbamoylmethoxy]-2,8,14,20-tetrathiacalix[4]arene (cone) (**3**), DMSO-d_6, 298 K, 400 MHz.

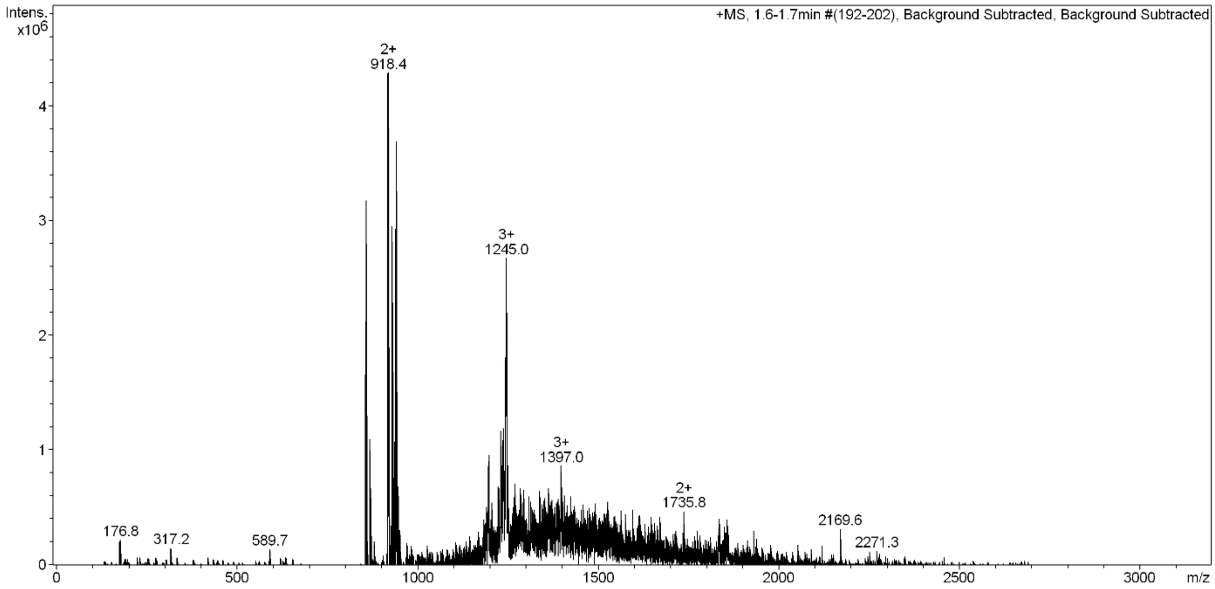

Figure S4. Mass spectrum (ESI) of 5,11,17,23-Tetra-*tert*-butyl-25,26,27,28-tetrakis[(N-(3’,3’-diethyl-3’-{3”-sulfonatopropyl})ammoniumethyl)-carbamoylmethoxy]-2,8,14,20-tetrathiacalix[4]arene (*cone*) **(3).**

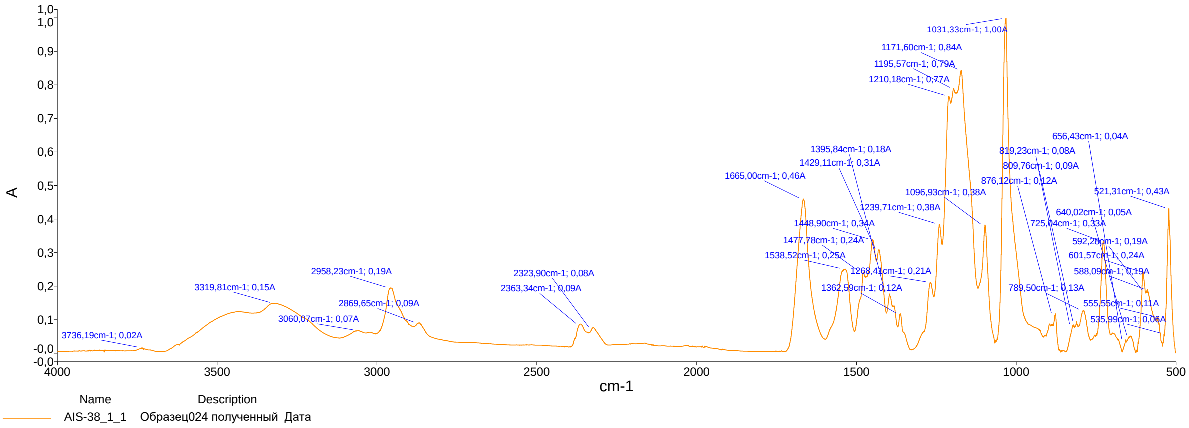

Figure S5. IR spectrum of 5,11,17,23-Tetra-*tert*-butyl-25,26,27,28-tetrakis[(N-(3’,3’-diethyl-3’-{3”-sulfonatopropyl})ammoniumethyl)-carbamoylmethoxy]-2,8,14,20-tetrathiacalix[4]arene (*cone*) **(3).**

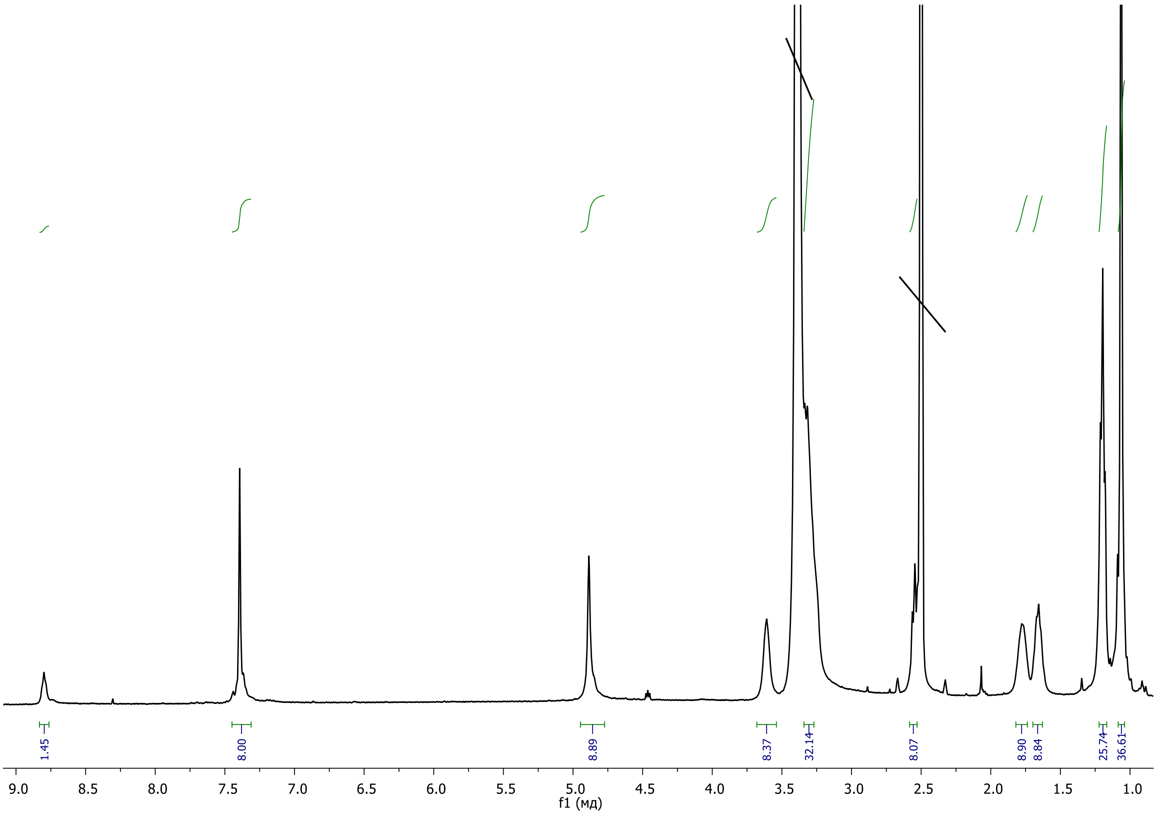

Figure S6. ^{1}H NMR spectrum of 5,11,17,23-Tetra-*tert*-butyl-25,26,27,28-tetrakis[(N-(3’,3’-diethyl-3’-{3”-sulfonatobutyl})ammoniumethyl)-carbamoylmethoxy]-2,8,14,20-tetrathiacalix[4]arene
(*cone*) **(4)**, DMSO-d_6, 298 K, 400 MHz.

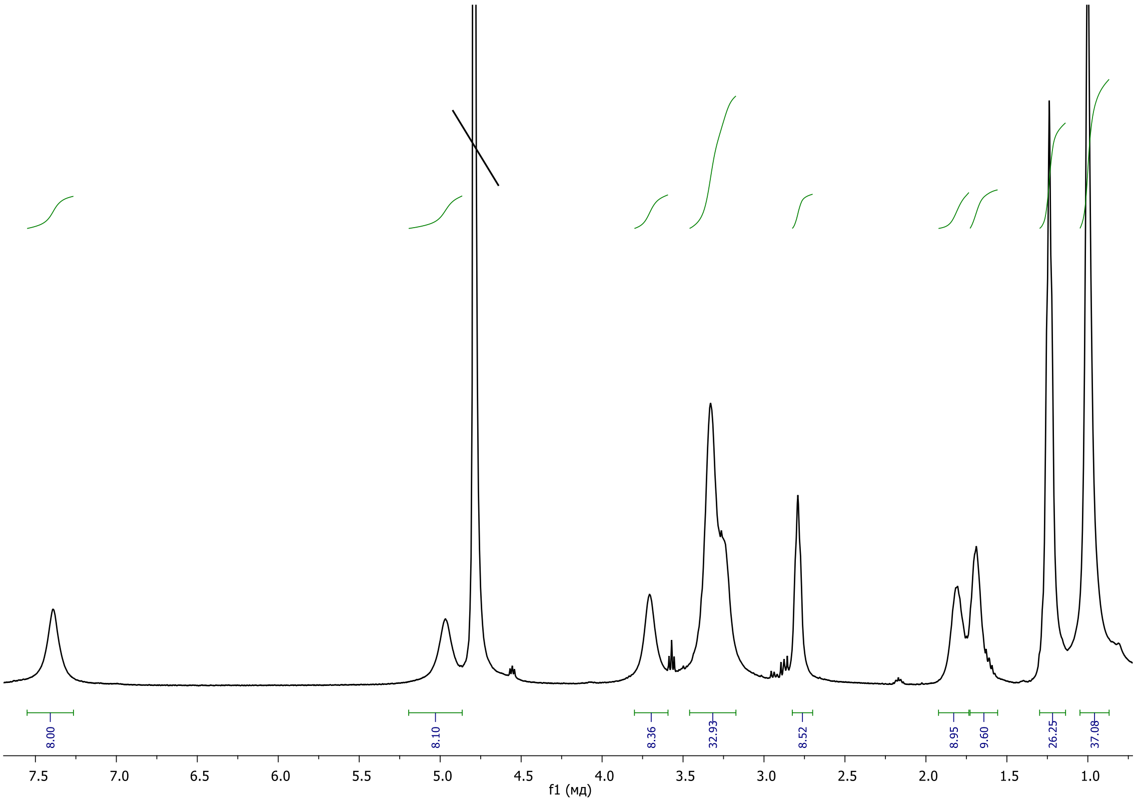

Figure S7. ^{1}H NMR spectrum of 5,11,17,23-Tetra-*tert*-butyl-25,26,27,28-tetrakis[(N-(3',3'-diethyl-3'-{3"-sulfonatobutyl})ammoniumethyl)-carbamoylmethoxy]-2,8,14,20-tetrathiacalix[4]arene
(*cone*) **(4)**, D_2O, 298 K, 400 MHz.

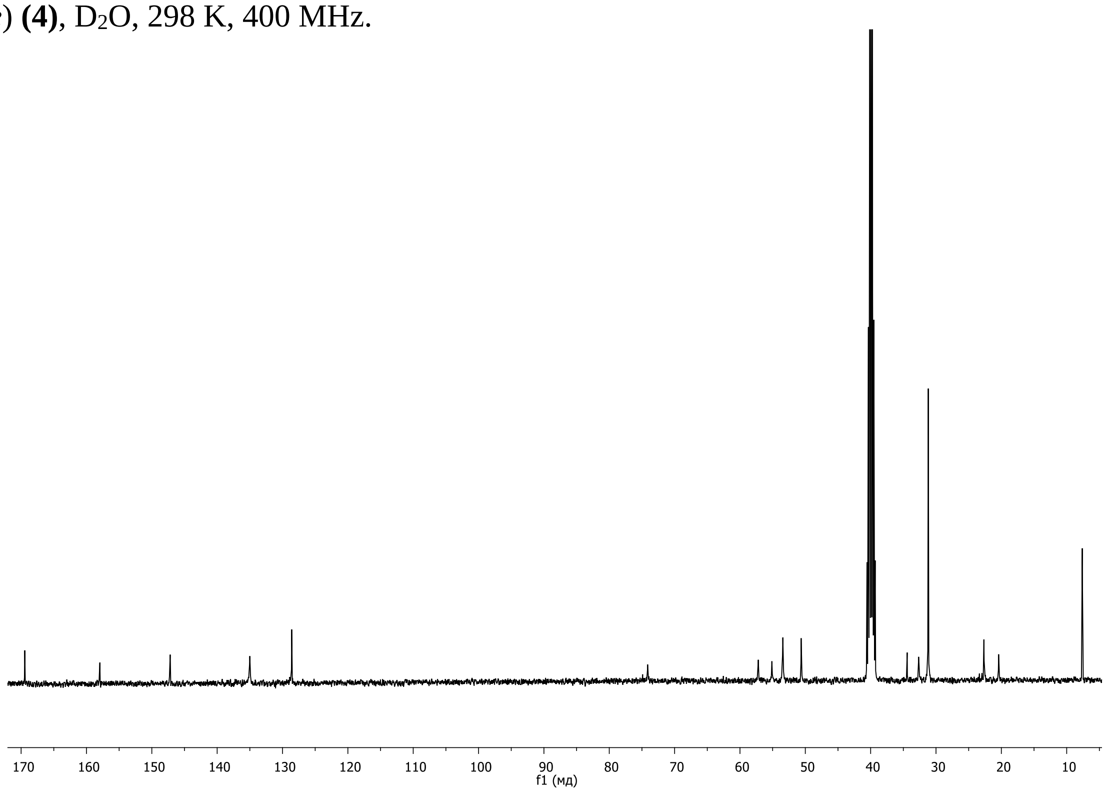

Figure S8. ^{13}C NMR spectrum of 5,11,17,23-Tetra-*tert*-butyl-25,26,27,28-tetrakis[(N-(3',3'-diethyl-3'-{3"-sulfonatobutyl})ammoniumethyl)-carbamoylmethoxy]-2,8,14,20-tetrathiacalix[4]arene (*cone*) **(4)**, DMSO-d_6, 298 K, 400 MHz.

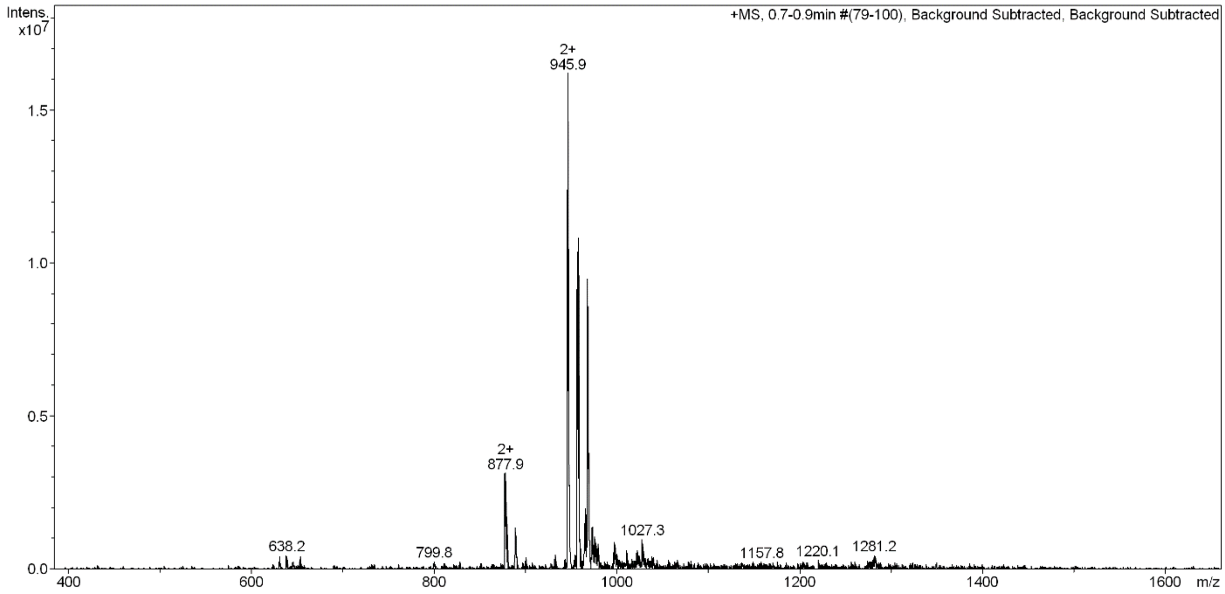

Figure S9. Mass spectrum (ESI) of 5,11,17,23-Tetra-*tert*-butyl-25,26,27,28-tetrakis[(N-(3’,3’-diethyl-3’-{3”-sulfonatobutyl})ammoniumethyl)-carbamoylmethoxy]-2,8,14,20-tetrathiacalix[4]arene (*cone*) **(4).**

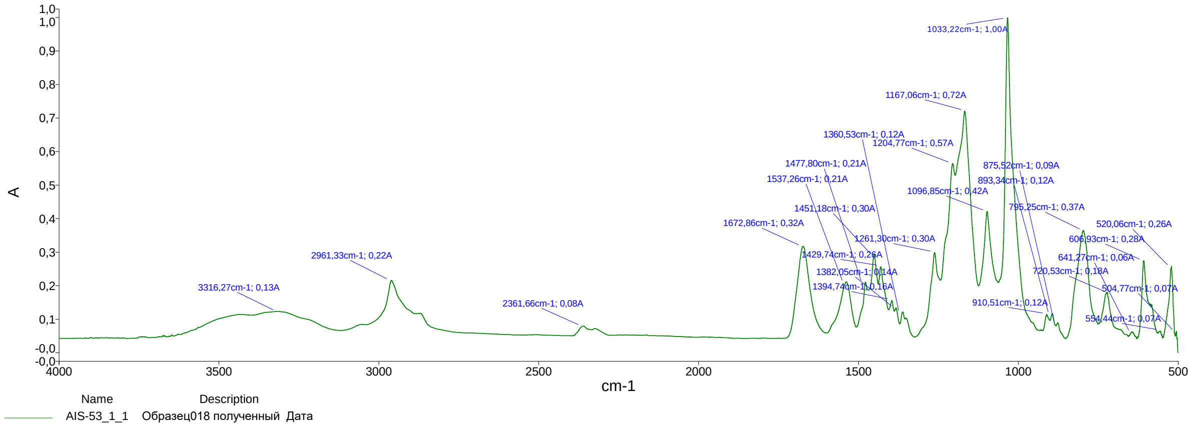

Figure S10. IR spectrum of 5,11,17,23-Tetra-*tert*-butyl-25,26,27,28-tetrakis[(N-(3’,3’-diethyl-3’-{3”-sulfonatobutyl})ammoniumethyl)-carbamoylmethoxy]-2,8,14,20-tetrathiacalix[4]arene
(*cone*) **(4).**

2. Dynamic light scattering

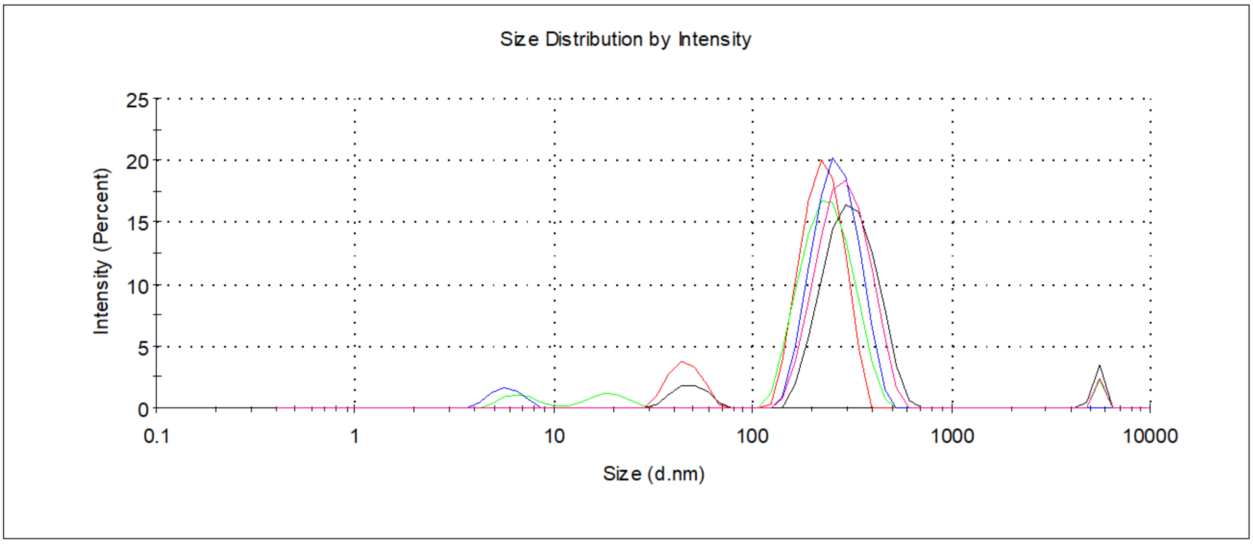

Figure S11. Size distribution of 5,11,17,23-tetra-tert-butyl-25,26,27,28-tetrakis[(N-(3',3'-diethyl- 3'-{3"-sulfonatopropyl})ammoniumethyl)-carbamoylmethoxy]-2,8,14,20-tetrathiacalix[4]arene (cone) (**3**) in phosphate buffer at 25°C (5×10^{-5} M).

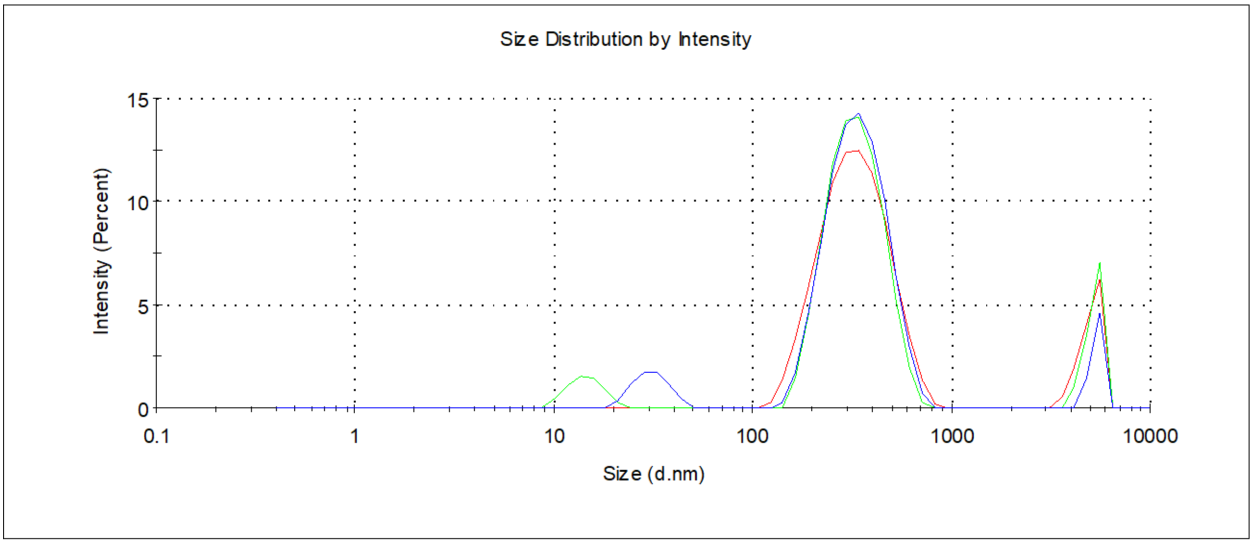

Figure S12. Size distribution of 5,11,17,23-tetra-tert-butyl-25,26,27,28-tetrakis[(N-(3',3'-diethyl- 3'-{3"-sulfonatopropyl})ammoniumethyl)-carbamoylmethoxy]-2,8,14,20-tetrathiacalix[4]arene (cone) (**3**) in phosphate buffer at 25°C (1×10^{-5} M).

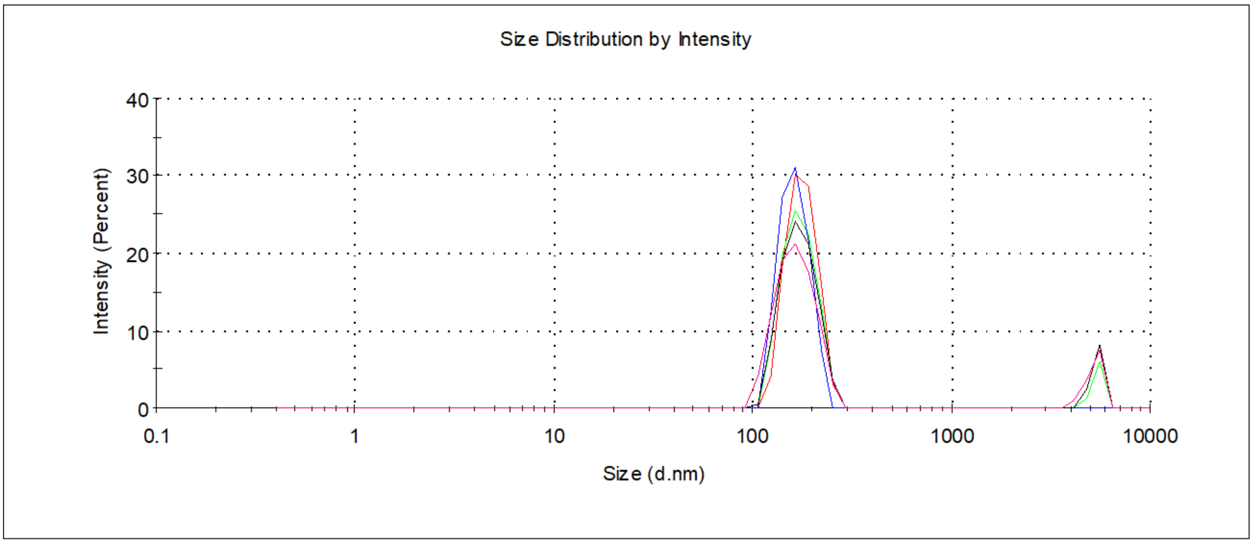

Figure S13. Size distribution of 5,11,17,23-tetra-tert-butyl-25,26,27,28-tetrakis[(N-(3’,3’-diethyl- 3’-{3”-sulfonatopropyl})ammoniumethyl)-carbamoylmethoxy]-2,8,14,20-tetrathiacalix[4]arene (cone) (**3**) in phosphate buffer at 25°C (5×10^{-6} M).

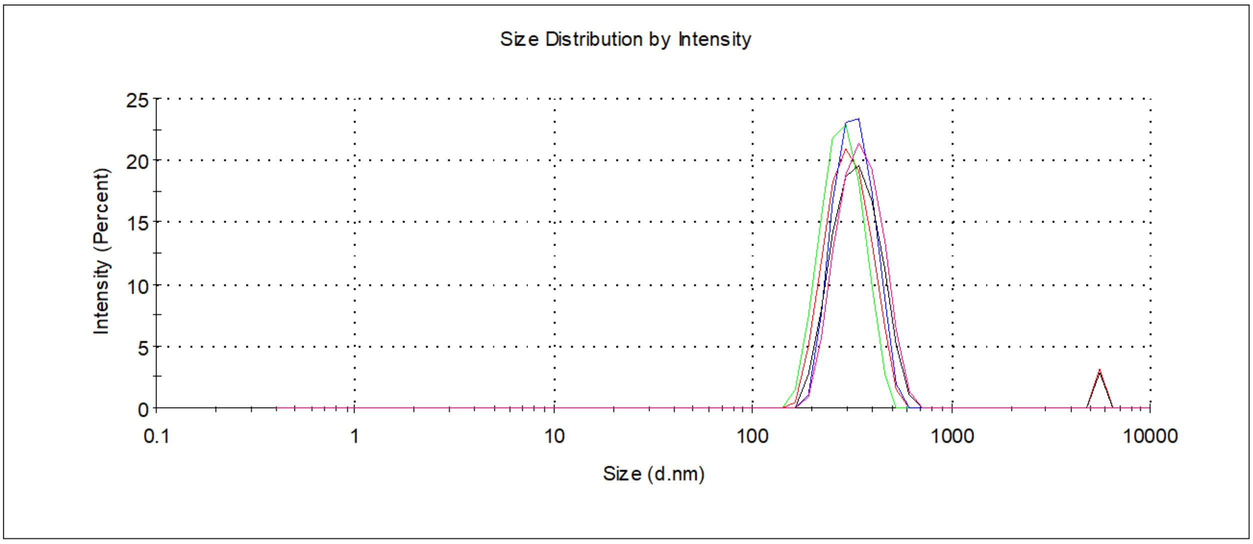

Figure S14. Size distribution of 5,11,17,23-tetra-tert-butyl-25,26,27,28-tetrakis[(N-(3’,3’-diethyl- 3’-{3”-sulfonatopropyl})ammoniumethyl)-carbamoylmethoxy]-2,8,14,20-tetrathiacalix[4]arene (cone) (**3**) in phosphate buffer at 25°C (1×10^{-6} M).

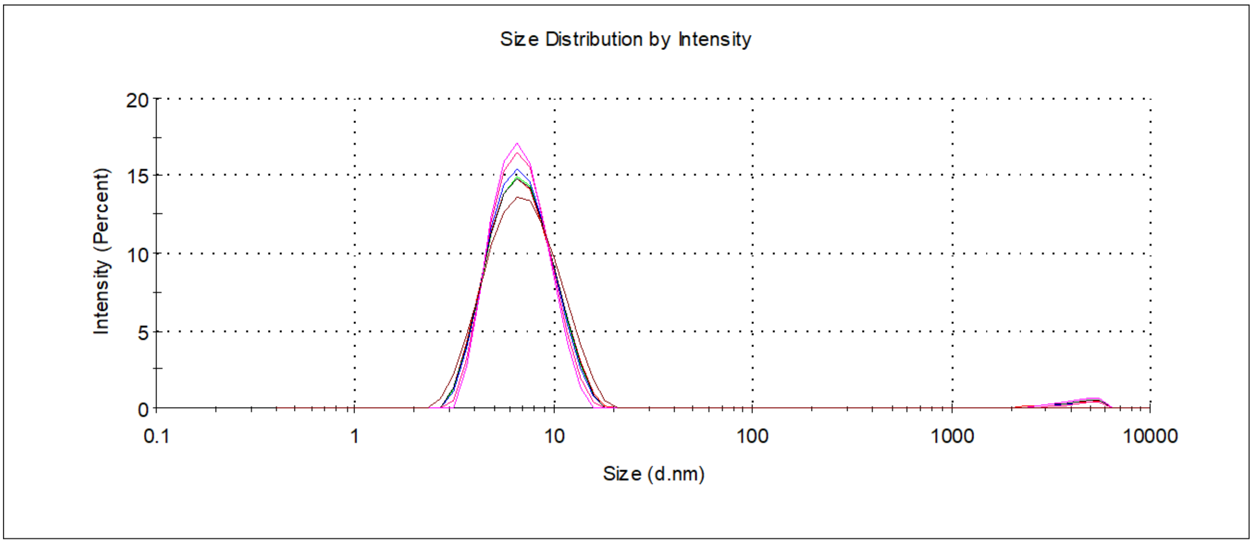

Figure S15. Size distribution of 5,11,17,23-tetra-tert-butyl-25,26,27,28-tetrakis[(N-(3’,3’-diethyl- 3’-{3”-sulfonatopropyl})ammoniumethyl)-carbamoylmethoxy]-2,8,14,20-tetrathiacalix[4]arene (cone) (**3**) in phosphate buffer with BSA (1:1) at 25°C (5×10^{-5} M).

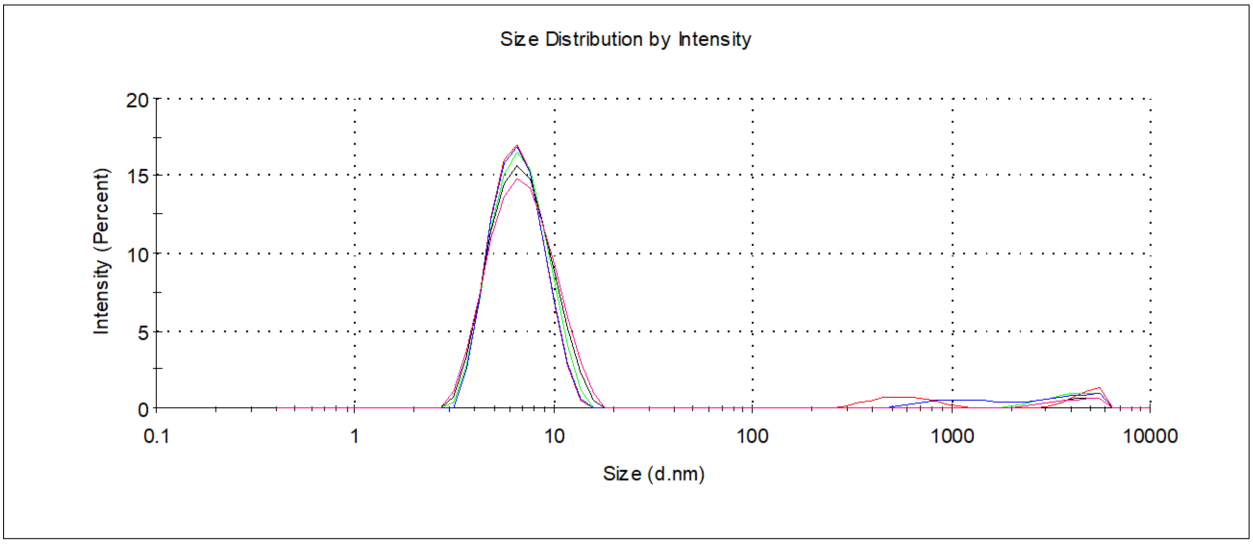

Figure S16. Size distribution of 5,11,17,23-tetra-tert-butyl-25,26,27,28-tetrakis[(N-(3’,3’-diethyl- 3’-{3”-sulfonatopropyl})ammoniumethyl)-carbamoylmethoxy]-2,8,14,20-tetrathiacalix[4]arene (cone) (**3**) in phosphate buffer with BSA (1:1) at 25°C (1×10^{-5} M).

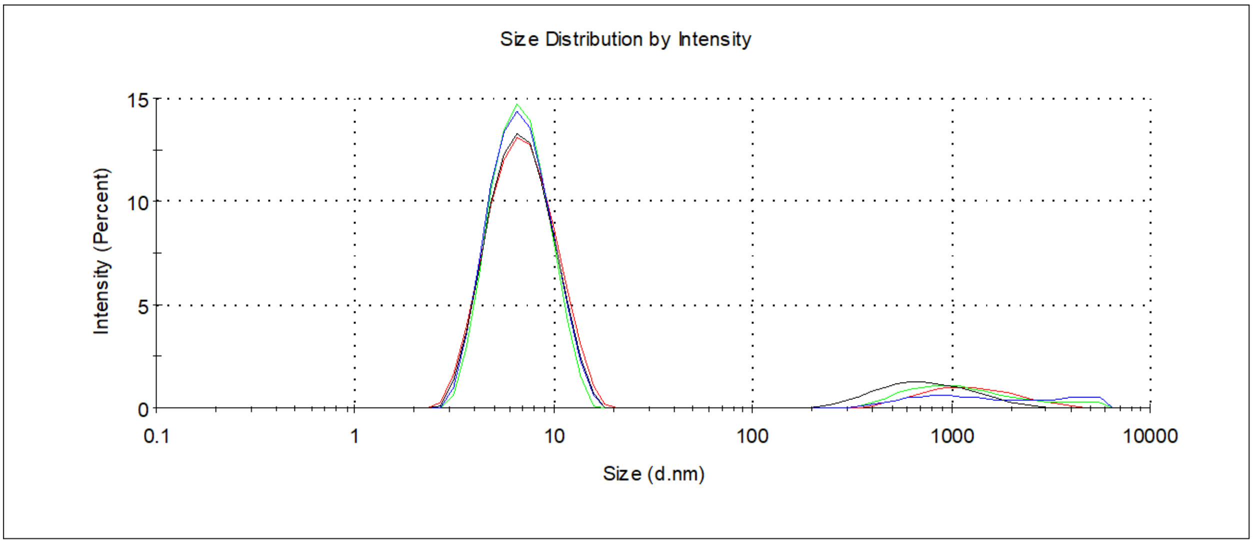

Figure S17. Size distribution of 5,11,17,23-tetra-tert-butyl-25,26,27,28-tetrakis[(N-(3’,3’-diethyl- 3’-{3”-sulfonatopropyl})ammoniumethyl)-carbamoylmethoxy]-2,8,14,20-tetrathiacalix[4]arene (cone) (**3**) in phosphate buffer with BSA (1:1) at 25°C (5×10^{-6} M).

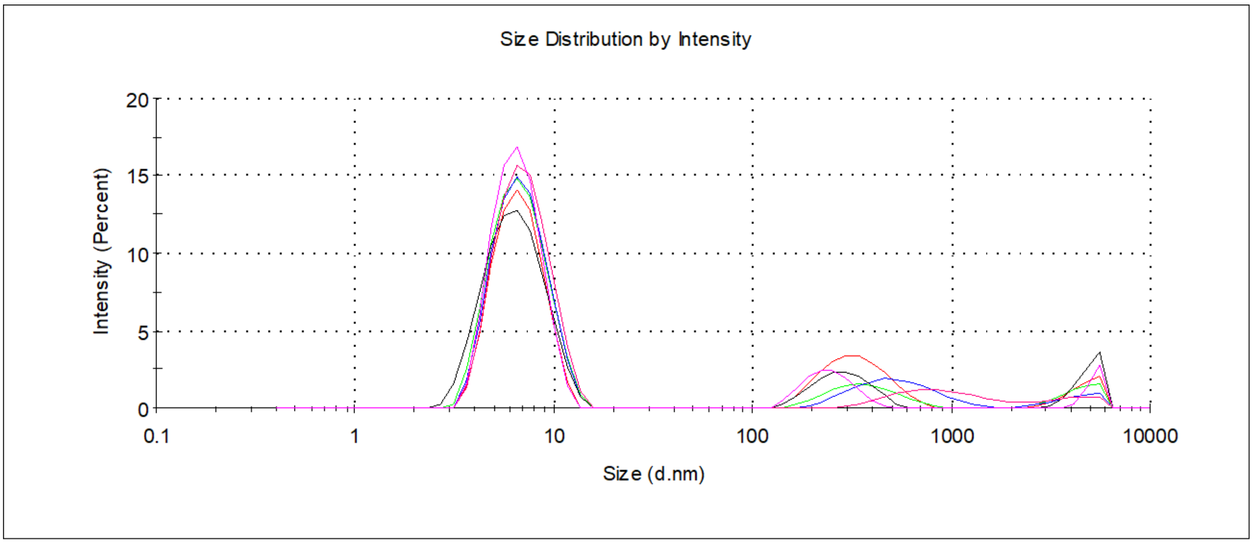

Figure S18. Size distribution of 5,11,17,23-tetra-tert-butyl-25,26,27,28-tetrakis[(N-(3’,3’-diethyl- 3’-{3”-sulfonatopropyl})ammoniumethyl)-carbamoylmethoxy]-2,8,14,20-tetrathiacalix[4]arene (cone) (**3**) in phosphate buffer with BSA (1:1) at 25°C (1×10^{-6} M).

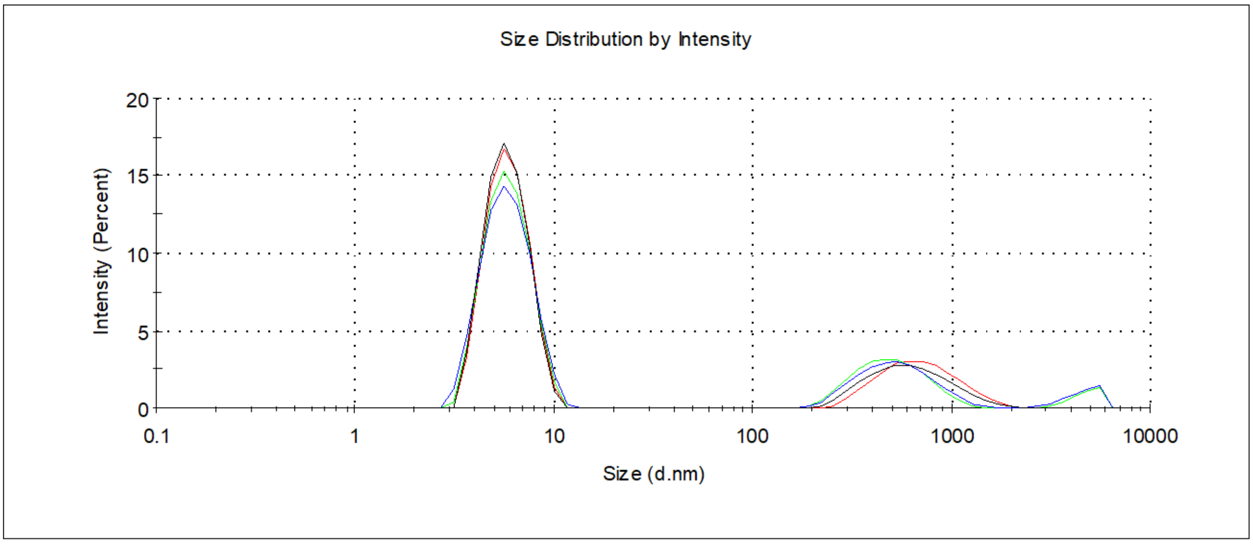

Figure S19. Size distribution of 5,11,17,23-tetra-tert-butyl-25,26,27,28-tetrakis[(N-(3’,3’-diethyl- 3’-{3”-sulfonatopropyl})ammoniumethyl)-carbamoylmethoxy]-2,8,14,20-tetrathiacalix[4]arene (cone) (**3**) in phosphate buffer with BSA (1:10) at 25°C (5×10^{-5} M/5×10^{-4} M).

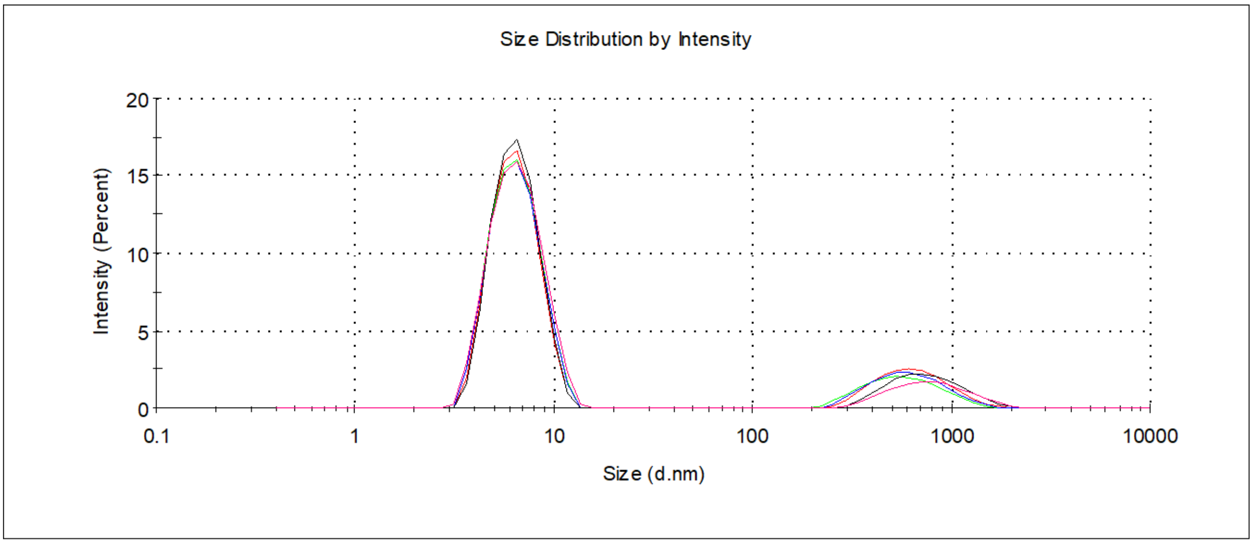

Figure S20. Size distribution of 5,11,17,23-tetra-tert-butyl-25,26,27,28-tetrakis[(N-(3’,3’-diethyl- 3’-{3”-sulfonatopropyl})ammoniumethyl)-carbamoylmethoxy]-2,8,14,20-tetrathiacalix[4]arene (cone) (**3**) in phosphate buffer with BSA (1:10) at 25°C (1×10^{-5} M/1×10^{-4} M).

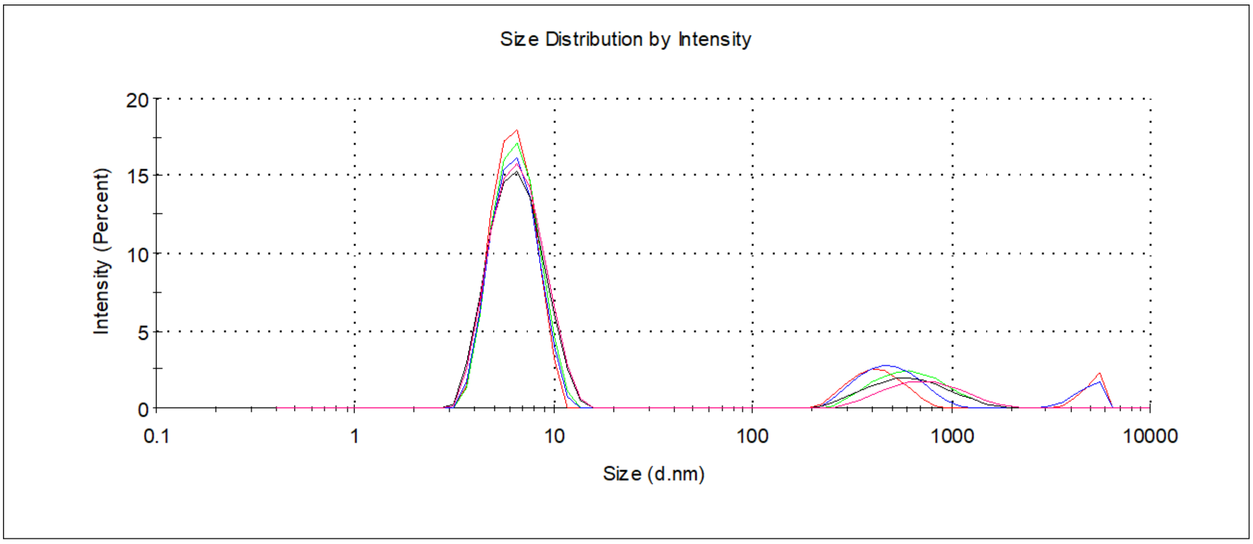

Figure S21. Size distribution of 5,11,17,23-tetra-tert-butyl-25,26,27,28-tetrakis[(N-(3',3'-diethyl- 3'-{3''-sulfonatopropyl})ammoniumethyl)-carbamoylmethoxy]-2,8,14,20-tetrathiacalix[4]arene (cone) (**3**) in phosphate buffer with BSA (1:10) at 25°C (5×10^{-6} M/5×10^{-5} M).

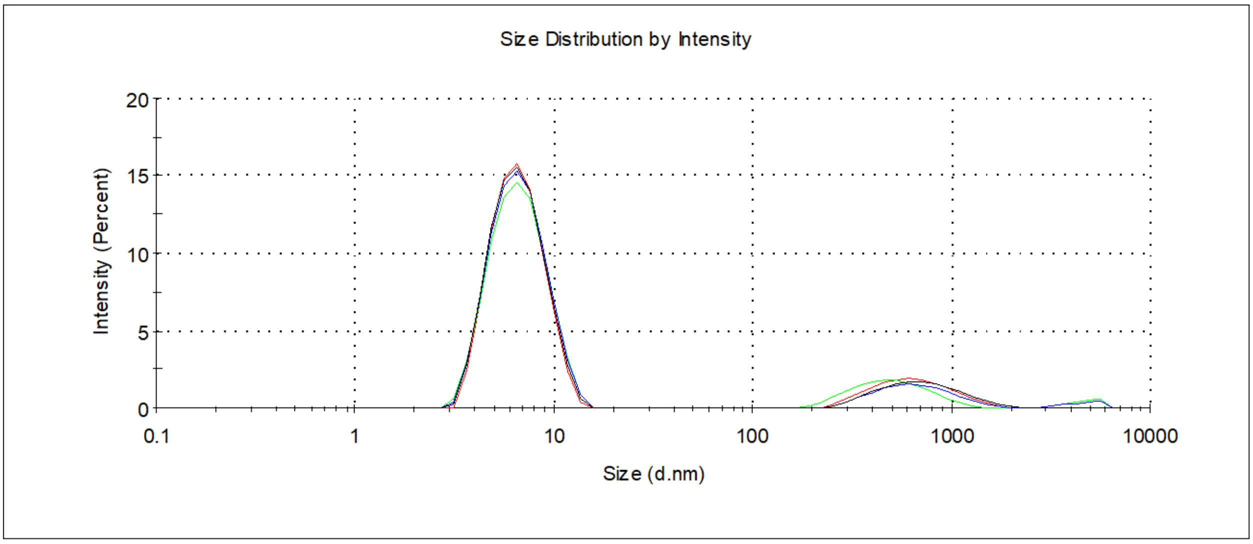

Figure S22. Size distribution of 5,11,17,23-tetra-tert-butyl-25,26,27,28-tetrakis[(N-(3',3'-diethyl- 3'-{3''-sulfonatopropyl})ammoniumethyl)-carbamoylmethoxy]-2,8,14,20-tetrathiacalix[4]arene (cone) (**3**) in phosphate buffer with BSA (1:10) at 25°C (1×10^{-6} M/1×10^{-5} M).

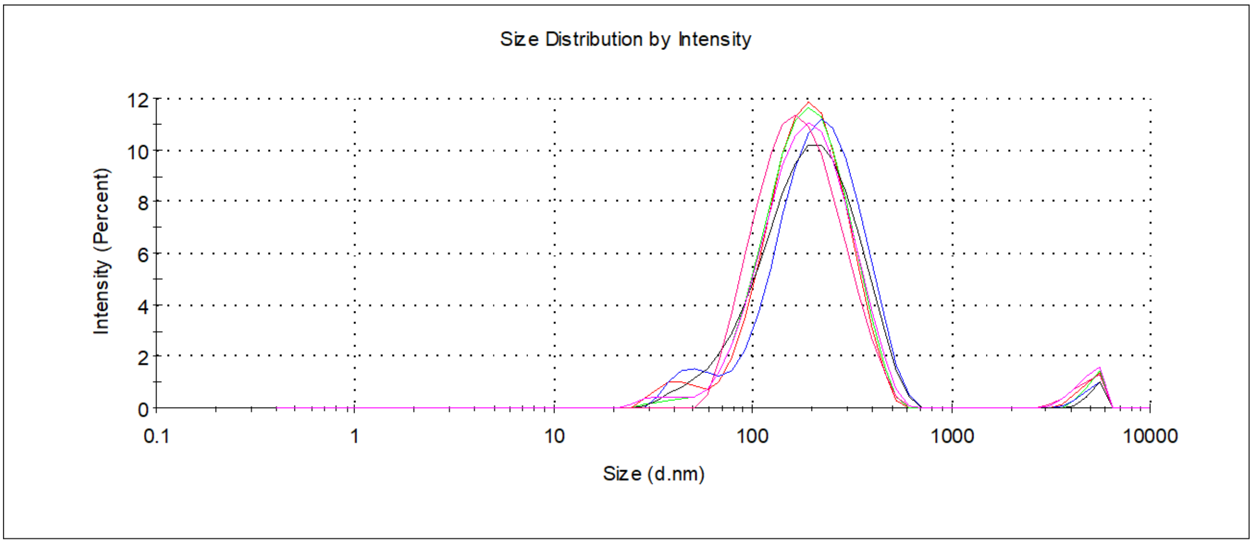

Figure S23. Size distribution of 5,11,17,23-tetra-tert-butyl-25,26,27,28-tetrakis[(N-(3’,3’-diethyl- 3’-{3”-sulfonatopropyl})ammoniumethyl)-carbamoylmethoxy]-2,8,14,20-tetrathiacalix[4]arene (cone) (**4**) in phosphate buffer at 25°C (5×10^{-5} M).

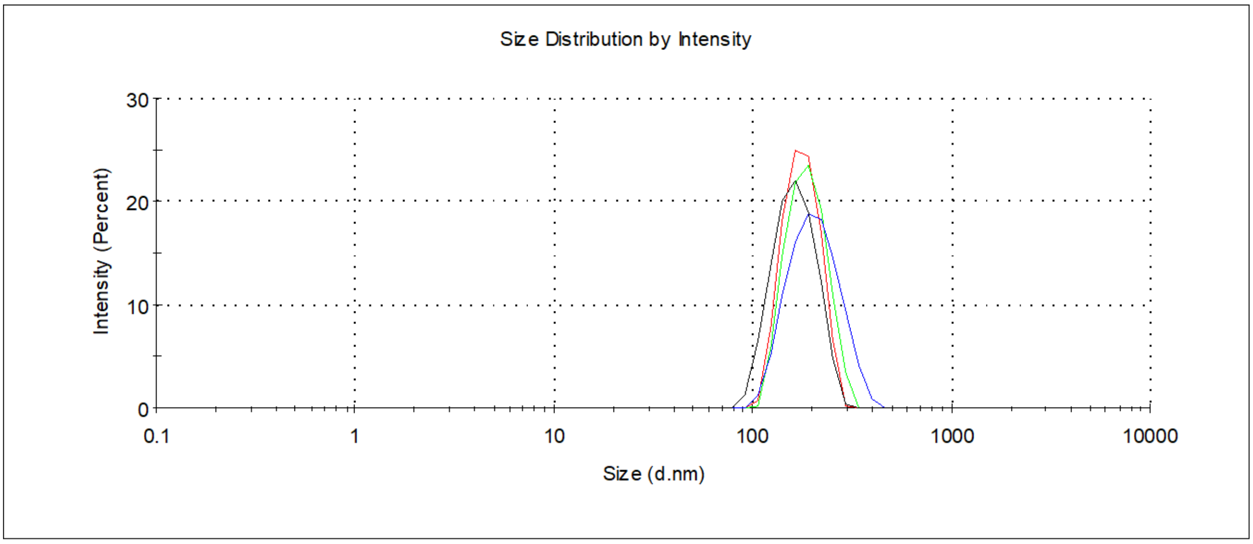

Figure S24. Size distribution of 5,11,17,23-tetra-tert-butyl-25,26,27,28-tetrakis[(N-(3’,3’-diethyl- 3’-{3”-sulfonatopropyl})ammoniumethyl)-carbamoylmethoxy]-2,8,14,20-tetrathiacalix[4]arene (cone) (**4**) in phosphate buffer at 25°C (1×10^{-5} M).

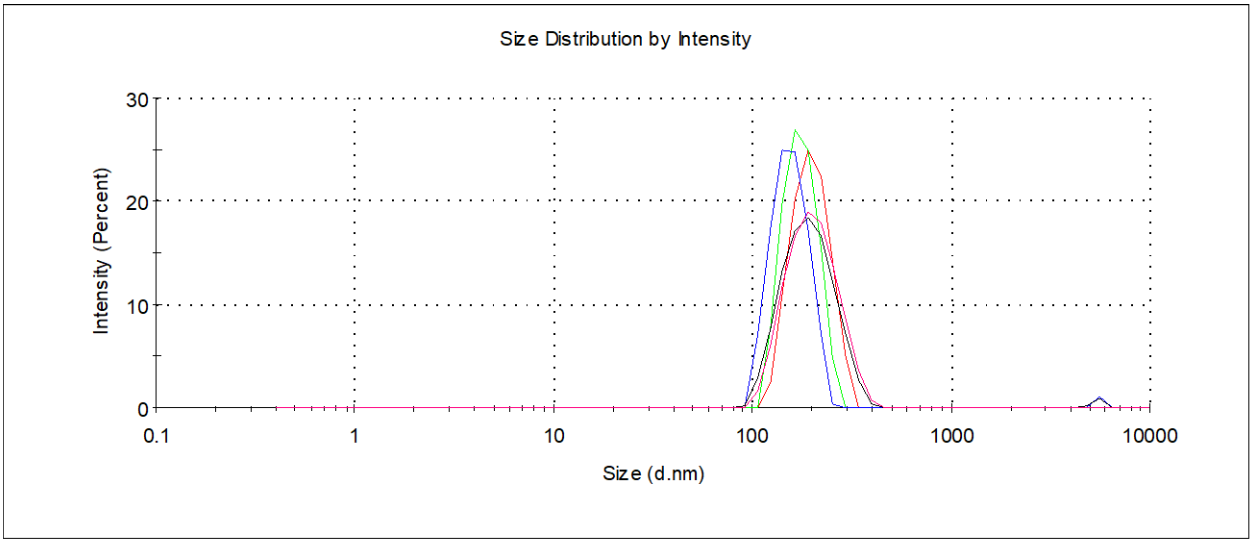

Figure S25. Size distribution of 5,11,17,23-tetra-tert-butyl-25,26,27,28-tetrakis[(N-(3’,3’-diethyl- 3’-{3”-sulfonatopropyl})ammoniumethyl)-carbamoylmethoxy]-2,8,14,20-tetrathiacalix[4]arene (cone) (**4**) in phosphate buffer at 25°C (5×10^{-6} M).

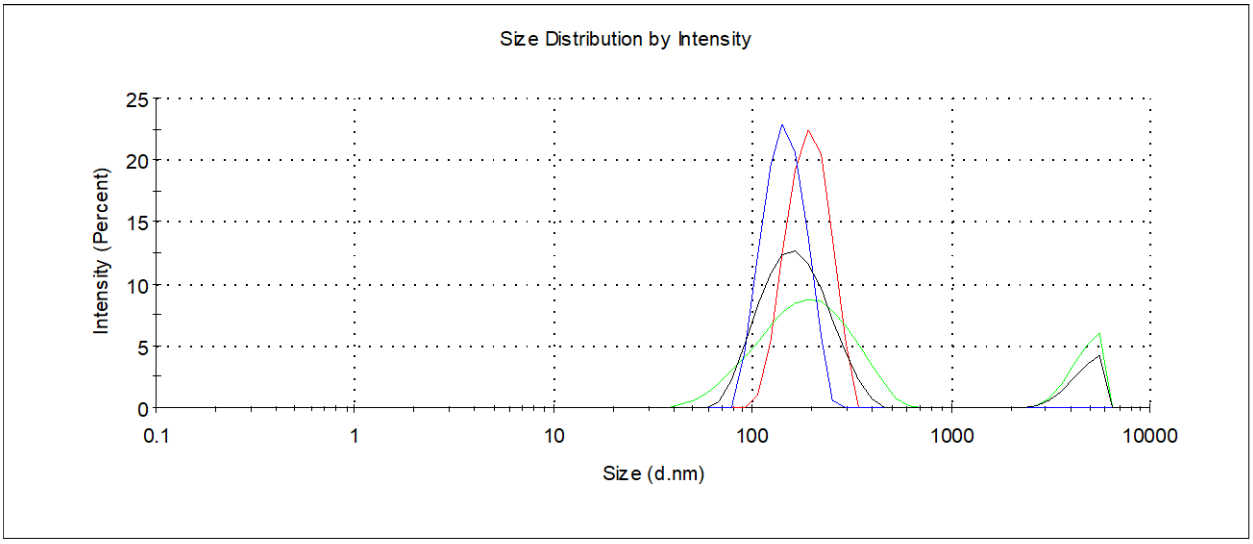

Figure S26. Size distribution of 5,11,17,23-tetra-tert-butyl-25,26,27,28-tetrakis[(N-(3’,3’-diethyl- 3’-{3”-sulfonatopropyl})ammoniumethyl)-carbamoylmethoxy]-2,8,14,20-tetrathiacalix[4]arene (cone) (**4**) in phosphate buffer at 25°C (1×10^{-6} M).

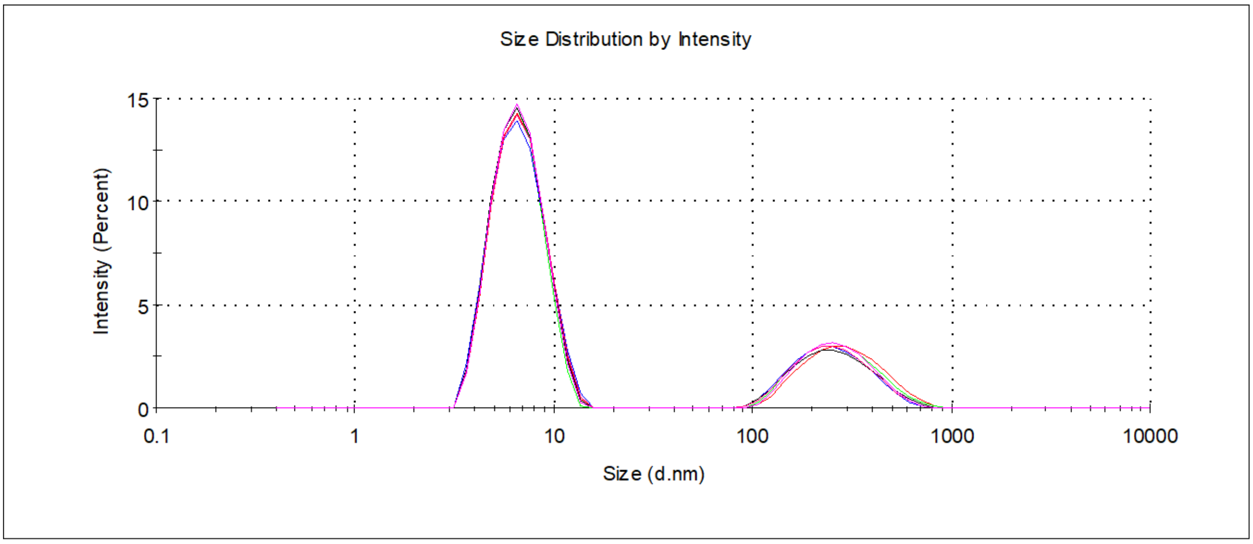

Figure S27. Size distribution of 5,11,17,23-tetra-tert-butyl-25,26,27,28-tetrakis[(N-(3',3'-diethyl- 3'-{3"-sulfonatopropyl})ammoniumethyl)-carbamoylmethoxy]-2,8,14,20-tetrathiacalix[4]arene (cone) (**4**) in phosphate buffer with BSA (1:1) at 25°C (5×10^{-5} M).

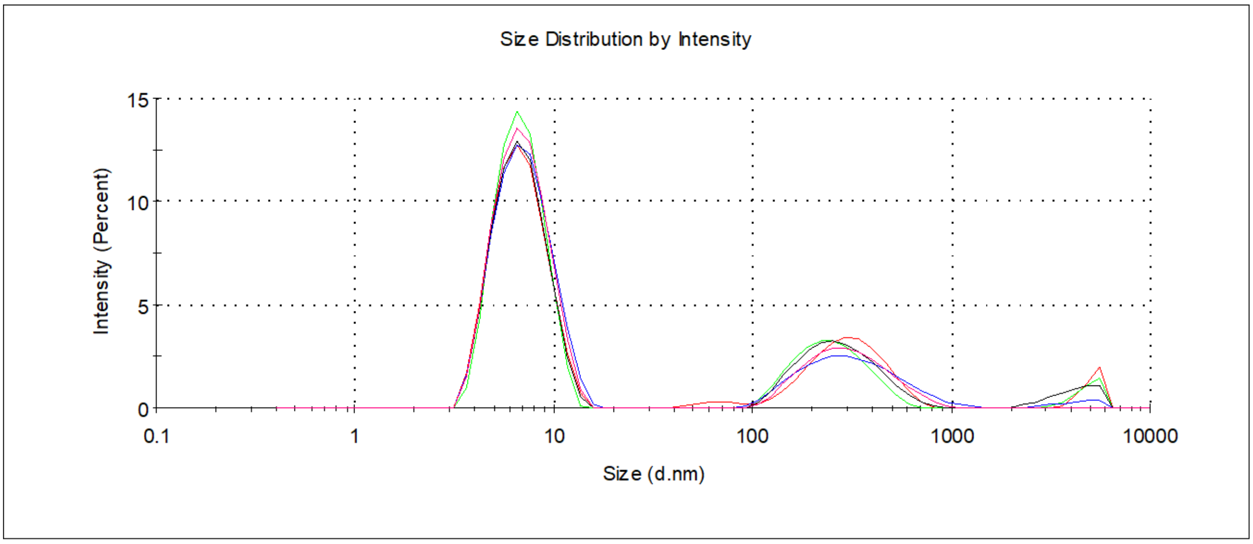

Figure S28. Size distribution of 5,11,17,23-tetra-tert-butyl-25,26,27,28-tetrakis[(N-(3',3'-diethyl- 3'-{3"-sulfonatopropyl})ammoniumethyl)-carbamoylmethoxy]-2,8,14,20-tetrathiacalix[4]arene (cone) (**4**) in phosphate buffer with BSA (1:1) at 25°C (1×10^{-5} M).

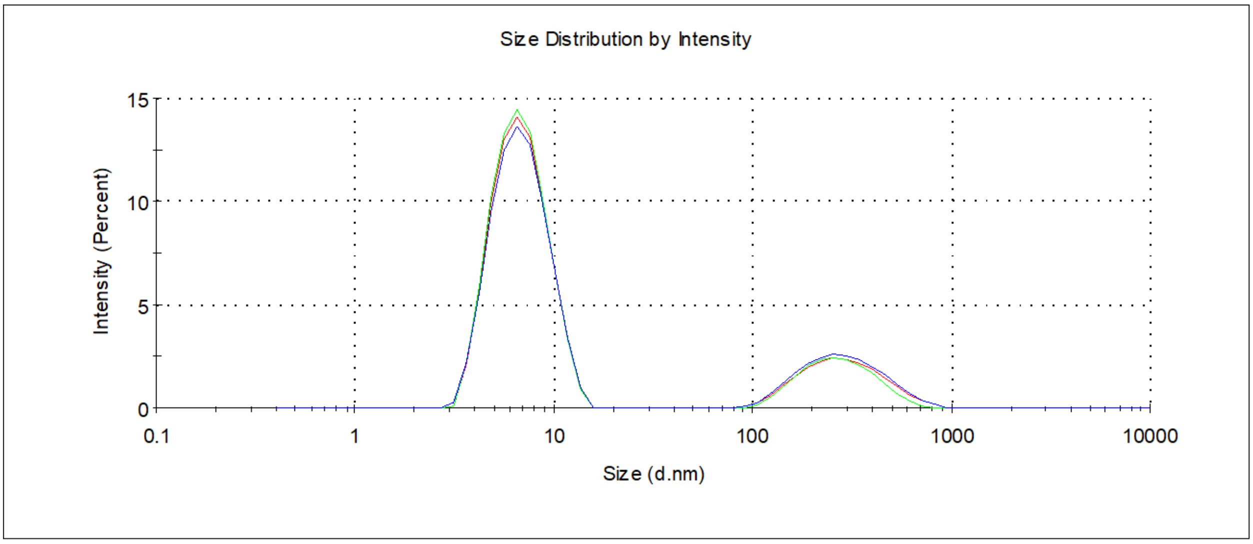

Figure S29. Size distribution of 5,11,17,23-tetra-tert-butyl-25,26,27,28-tetrakis[(N-(3’,3’-diethyl- 3’-{3”-sulfonatopropyl})ammoniumethyl)-carbamoylmethoxy]-2,8,14,20-tetrathiacalix[4]arene (cone) (**4**) in phosphate buffer with BSA (1:1) at 25°C (5×10^{-6} M).

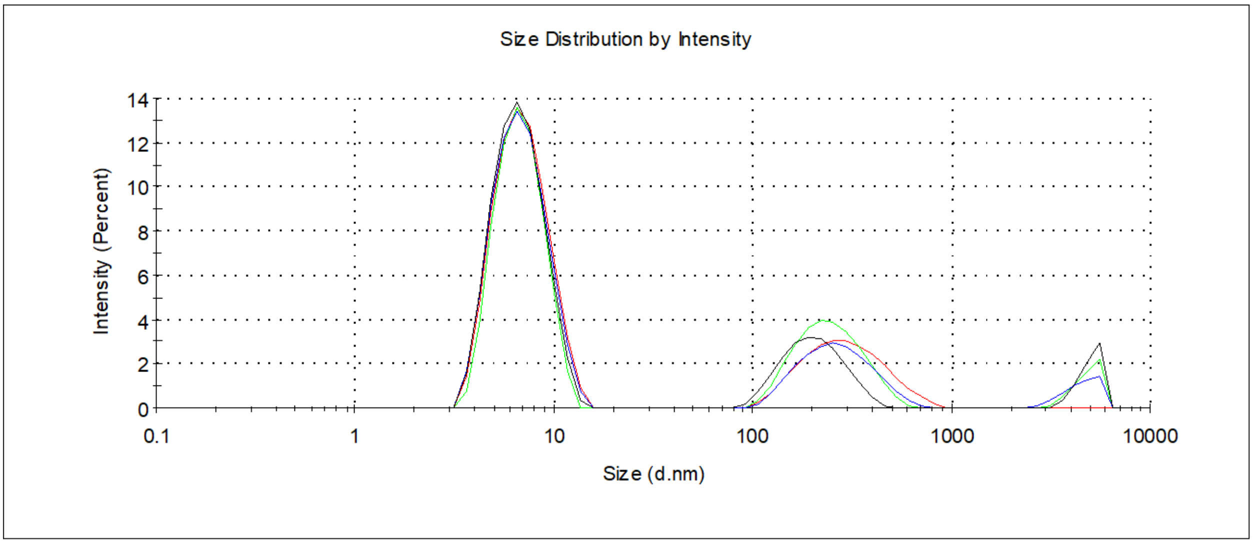

Figure S30. Size distribution of 5,11,17,23-tetra-tert-butyl-25,26,27,28-tetrakis[(N-(3’,3’-diethyl- 3’-{3”-sulfonatopropyl})ammoniumethyl)-carbamoylmethoxy]-2,8,14,20-tetrathiacalix[4]arene (cone) (**4**) in phosphate buffer with BSA (1:1) at 25°C (1×10^{-6} M).

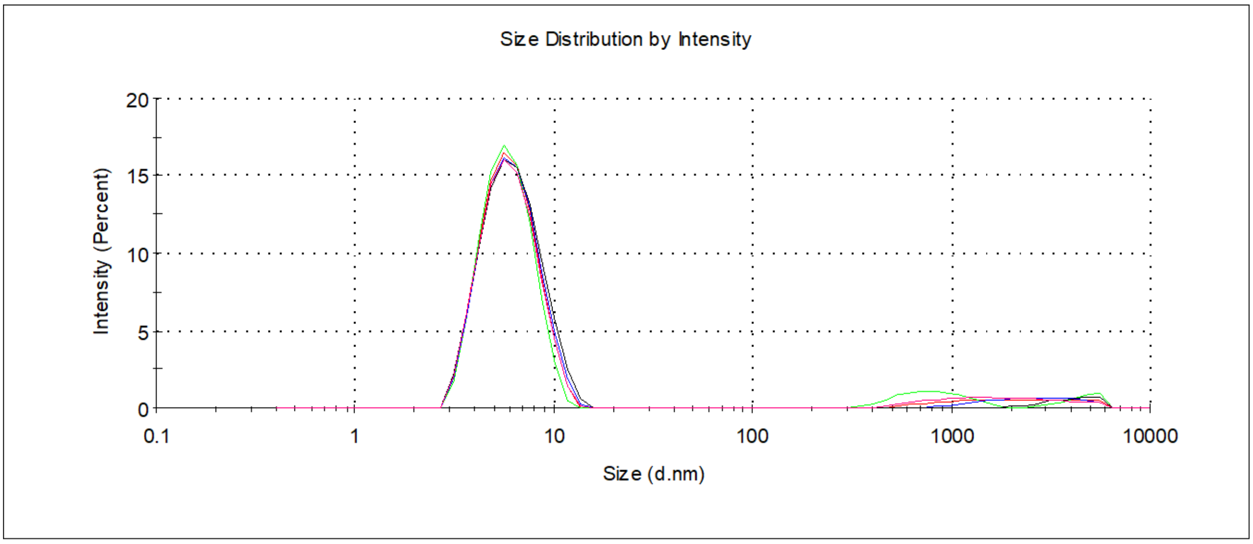

Figure S31. Size distribution of 5,11,17,23-tetra-tert-butyl-25,26,27,28-tetrakis[(N-(3',3'-diethyl- 3'-{3"-sulfonatopropyl})ammoniumethyl)-carbamoylmethoxy]-2,8,14,20-tetrathiacalix[4]arene (cone) (**4**) in phosphate buffer with BSA (1:10) at 25°C (5×10^{-5} M/5×10^{-4} M).

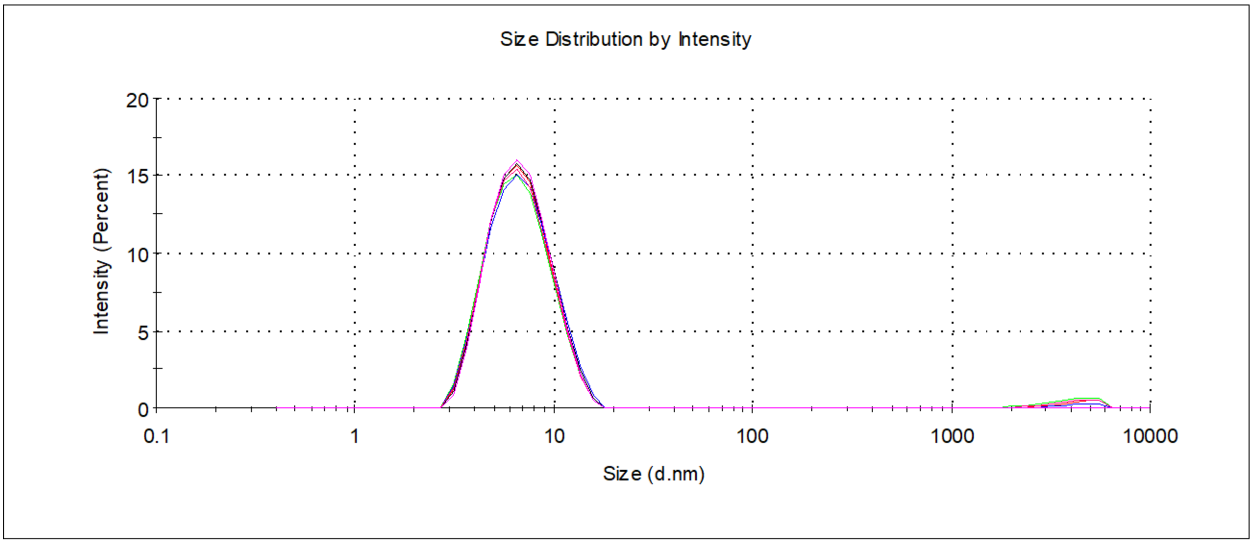

Figure S32. Size distribution of 5,11,17,23-tetra-tert-butyl-25,26,27,28-tetrakis[(N-(3',3'-diethyl- 3'-{3"-sulfonatopropyl})ammoniumethyl)-carbamoylmethoxy]-2,8,14,20-tetrathiacalix[4]arene (cone) (**4**) in phosphate buffer with BSA (1:10) at 25°C (1×10^{-5} M/1×10^{-4} M).

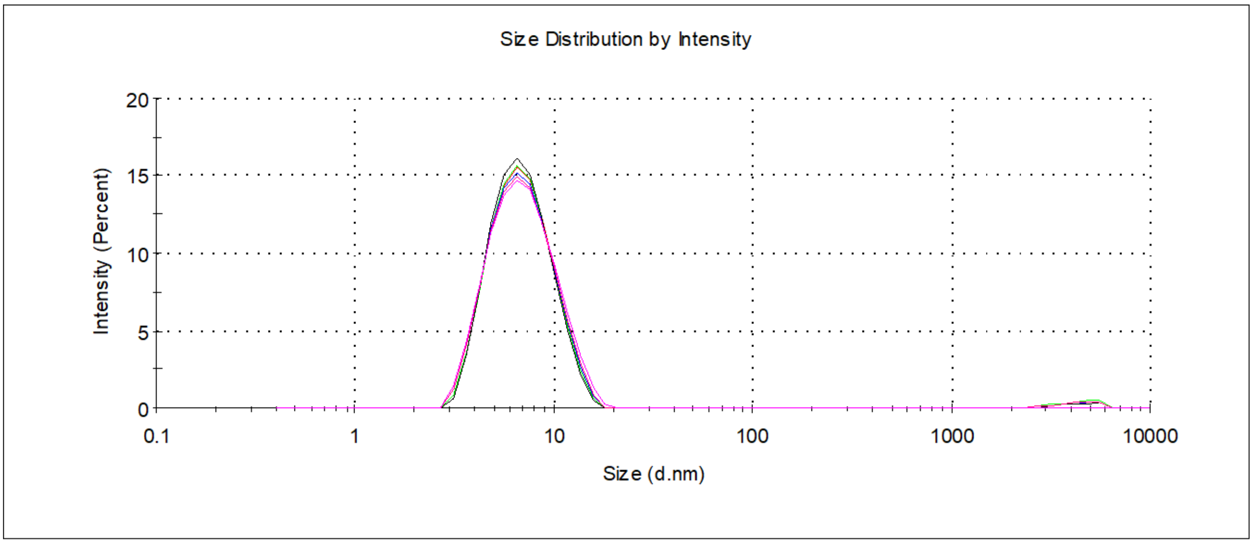

Figure S33. Size distribution of 5,11,17,23-tetra-tert-butyl-25,26,27,28-tetrakis[(N-(3',3'-diethyl- 3'-{3''-sulfonatopropyl})ammoniumethyl)-carbamoylmethoxy]-2,8,14,20-tetrathiacalix[4]arene (cone) (**4**) in phosphate buffer with BSA (1:10) at 25°C (5×10^{-6} M/5×10^{-5} M).

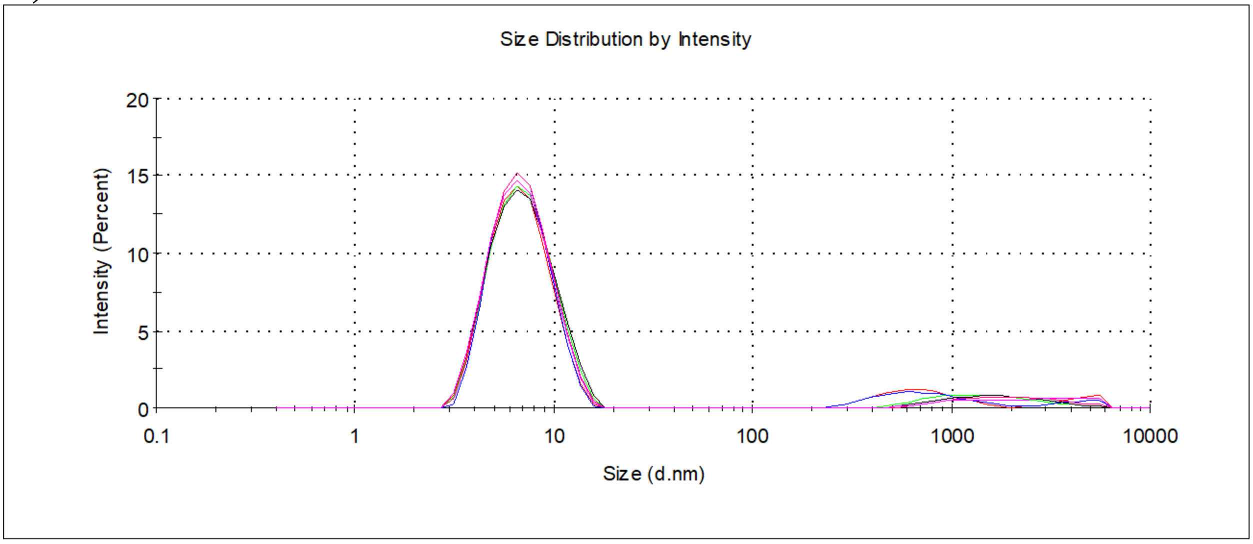

Figure S34. Size distribution of 5,11,17,23-tetra-tert-butyl-25,26,27,28-tetrakis[(N-(3',3'-diethyl- 3'-{3''-sulfonatopropyl})ammoniumethyl)-carbamoylmethoxy]-2,8,14,20-tetrathiacalix[4]arene (cone) (**4**) in phosphate buffer with BSA (1:10) at 25°C (1×10^{-6} M/1×10^{-5} M).

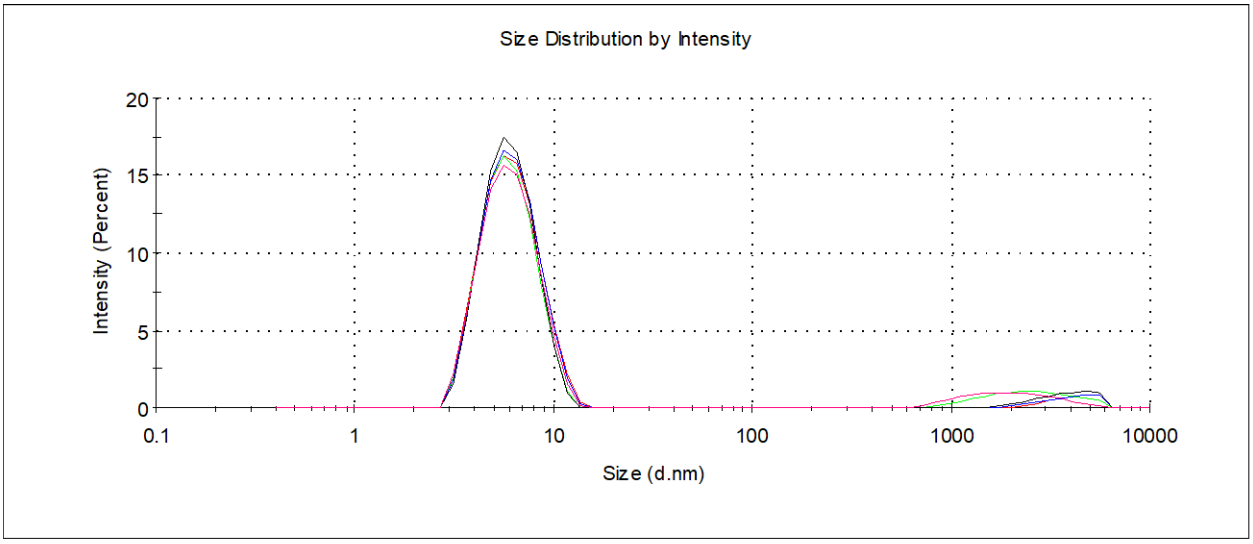

Figure S35. Size distribution of BSA in phosphate buffer at 25°C (5×10^{-4} M).

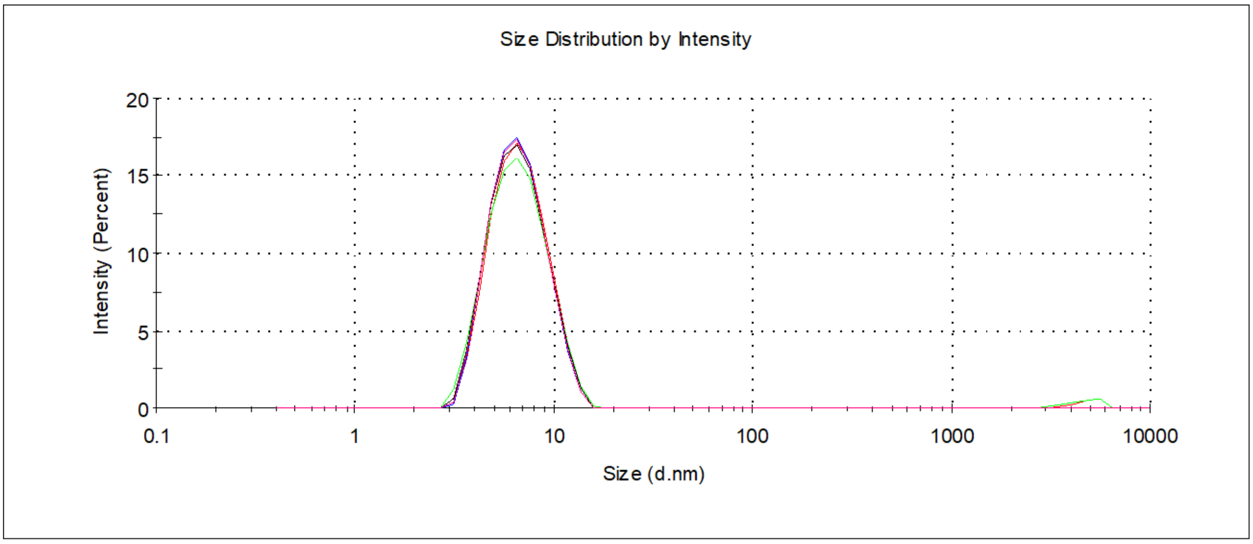

Figure S36. Size distribution of BSA in phosphate buffer at 25°C (1×10^{-4} M).

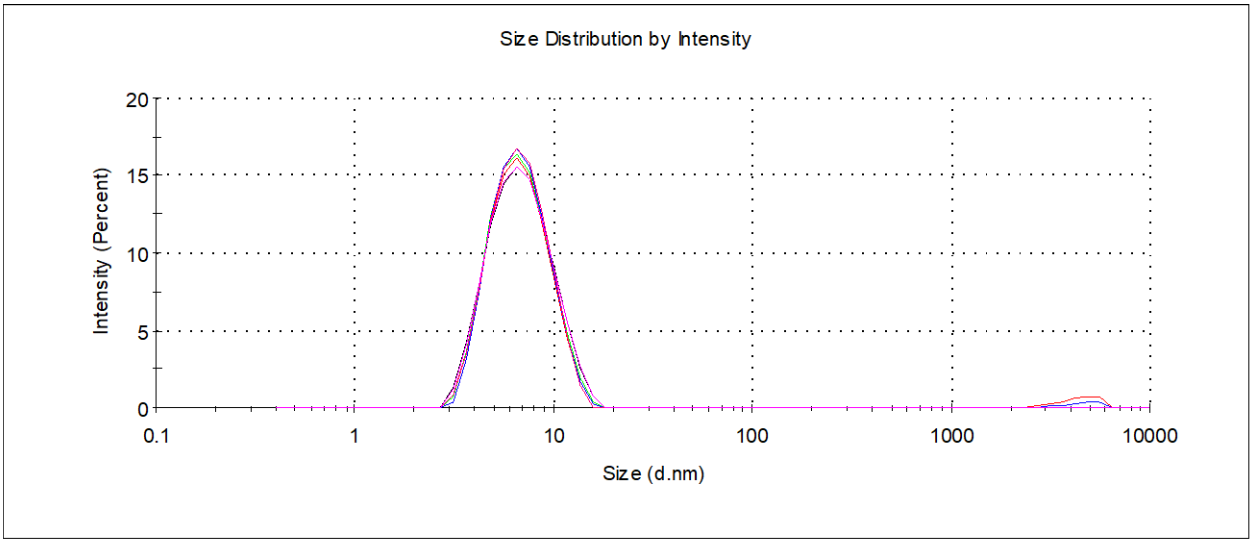

Figure S37. Size distribution of BSA in phosphate buffer at 25°C (5×10^{-5} M).

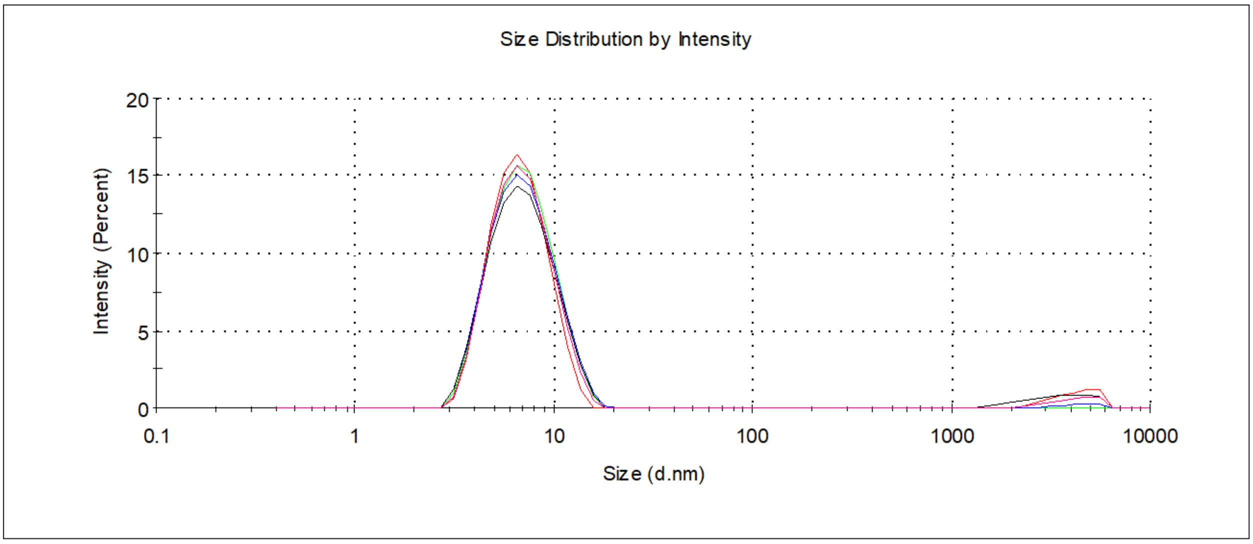

Figure S38. Size distribution of BSA in phosphate buffer at 25°C (1×10^{-5} M).

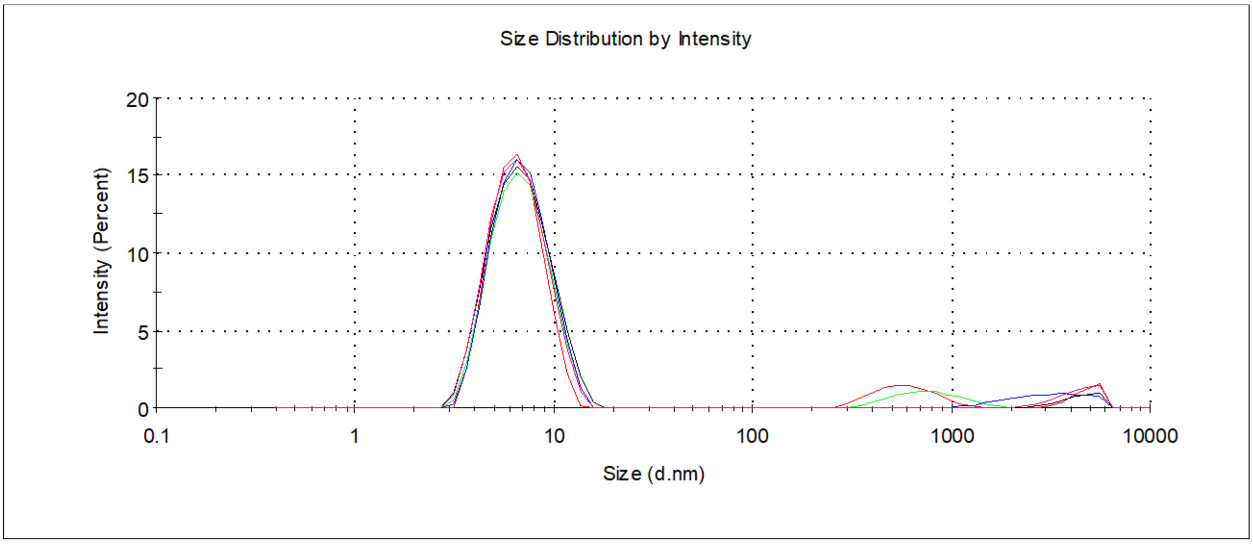

Figure S39. Size distribution of BSA in phosphate buffer at 25°C (5×10^{-6} M).

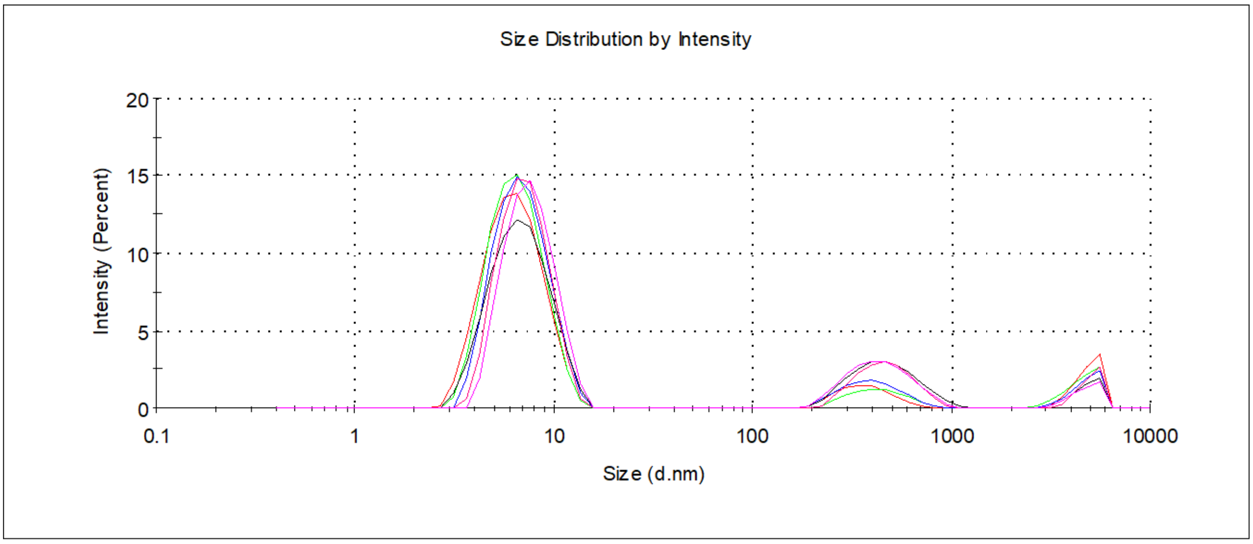

Figure S40. Size distribution of BSA in phosphate buffer at 25°C (1×10^{-6} M).

3. UV spectra

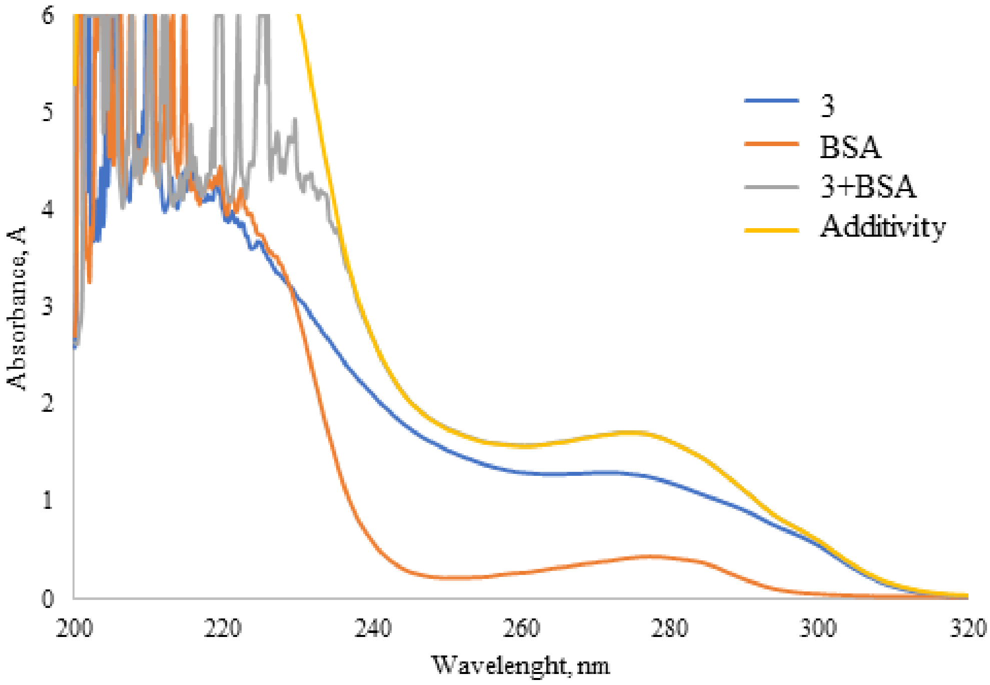

Figure S41. UV-*vis* spectra of BSA (300 mkM) in the presence of **3** (300 mkM) in phosphate buffer (pH=7.4).

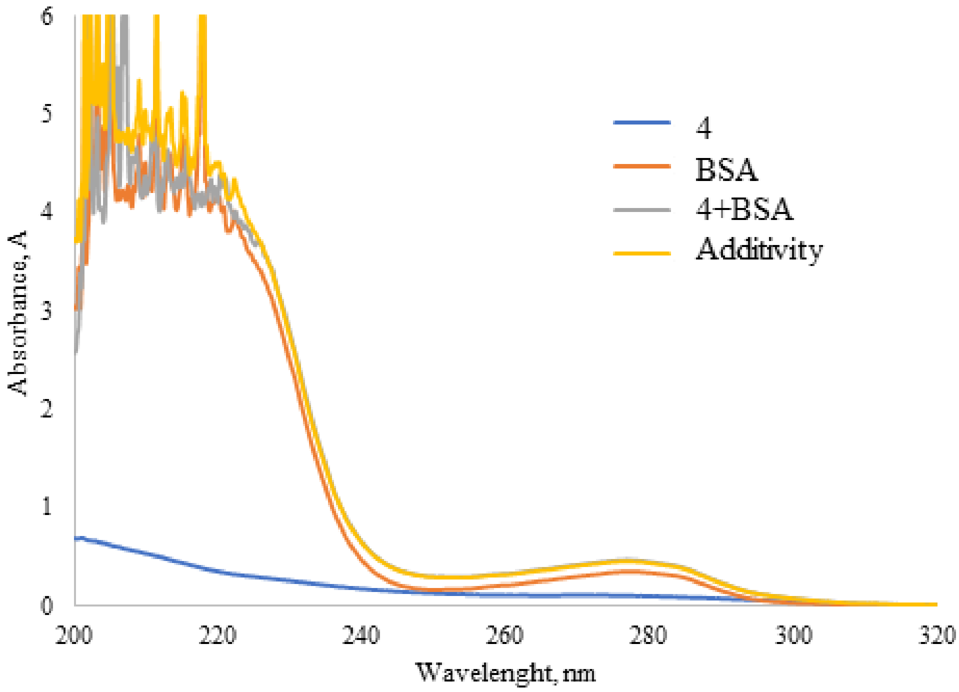

Figure S42. UV-*vis* spectra of BSA (300 mkM) in the presence of **4** (300 mkM) in phosphate buffer (pH=7.4).

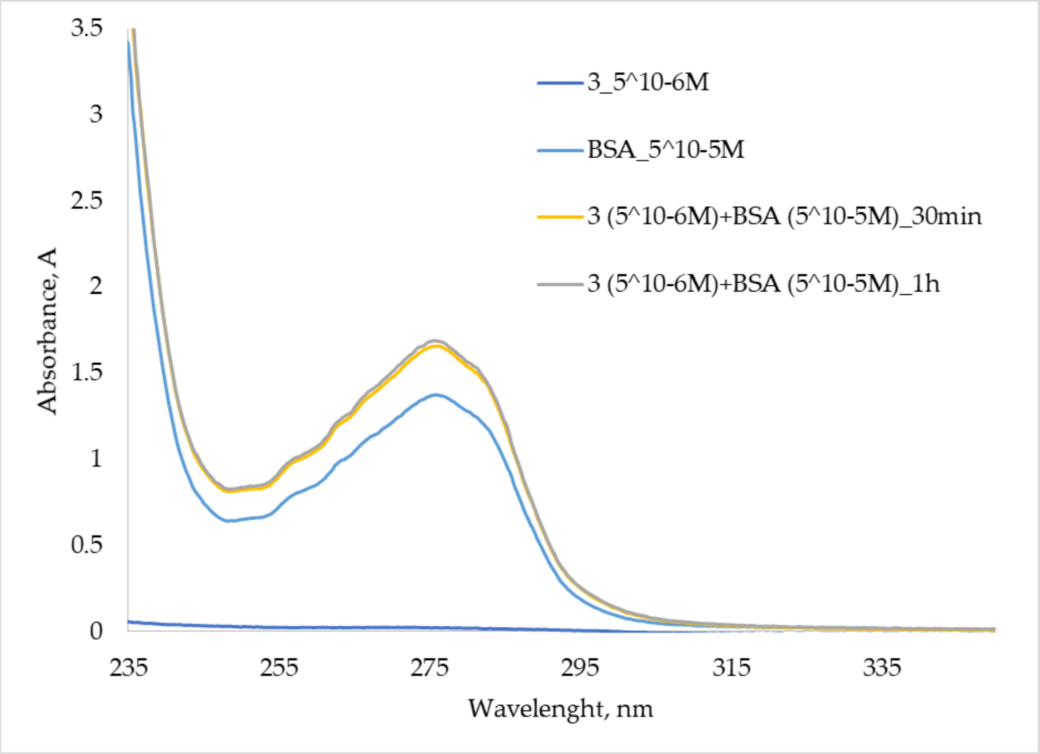

Figure S43. UV-*vis* spectra of BSA in the presence of **3** in phosphate buffer (pH=7.4).

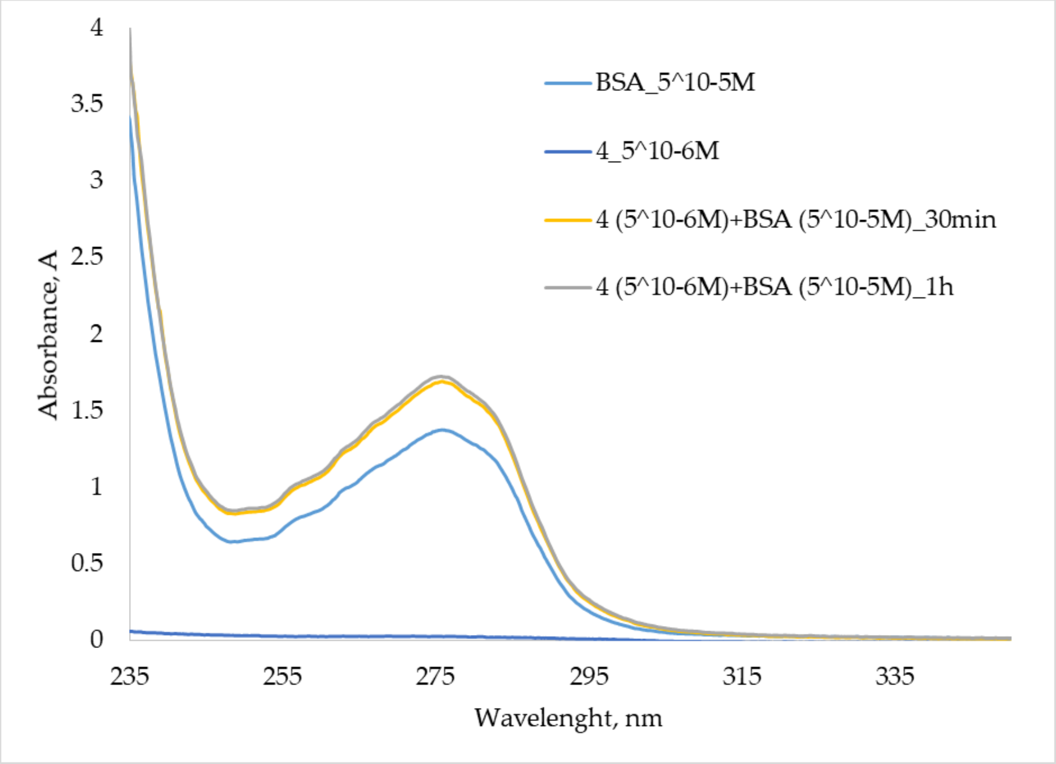

Figure S44. UV-*vis* spectra of BSA in the presence of **4** in phosphate buffer (pH=7.4).

4. Fluorescence

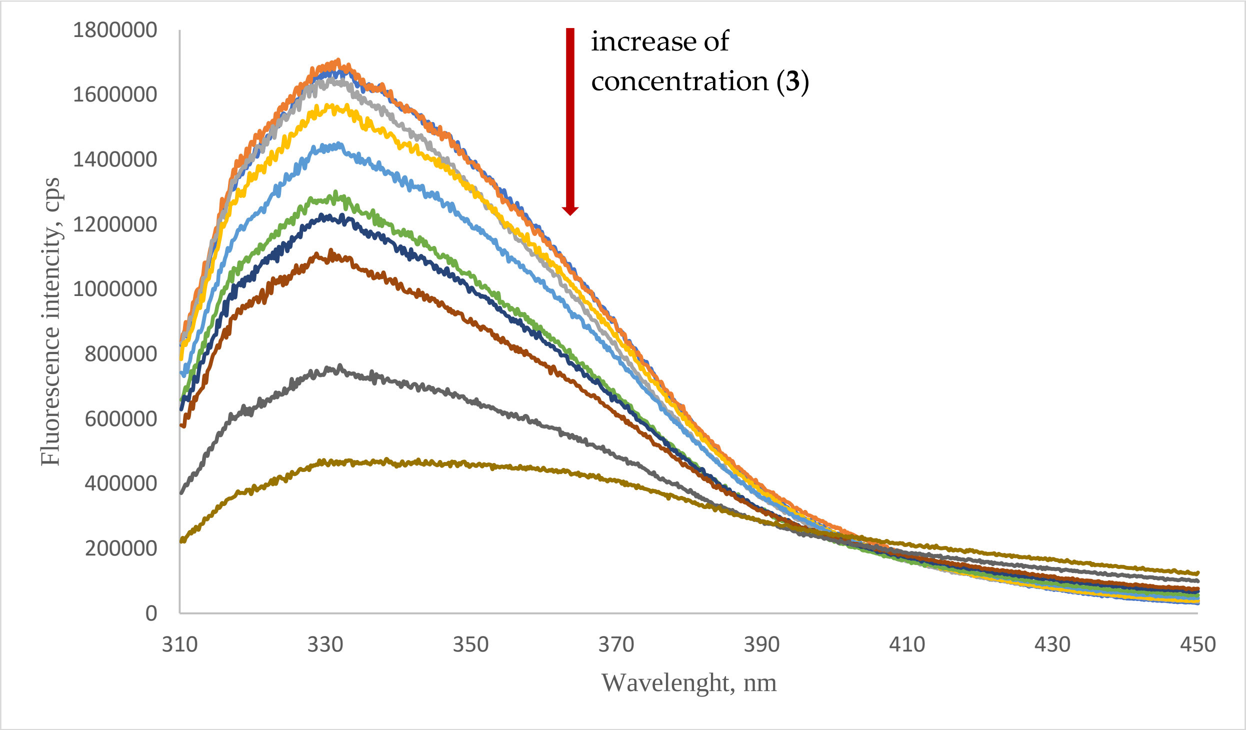

Figure S45. Fluorescence spectra of BSA in the presence of **3** at 5 °C.

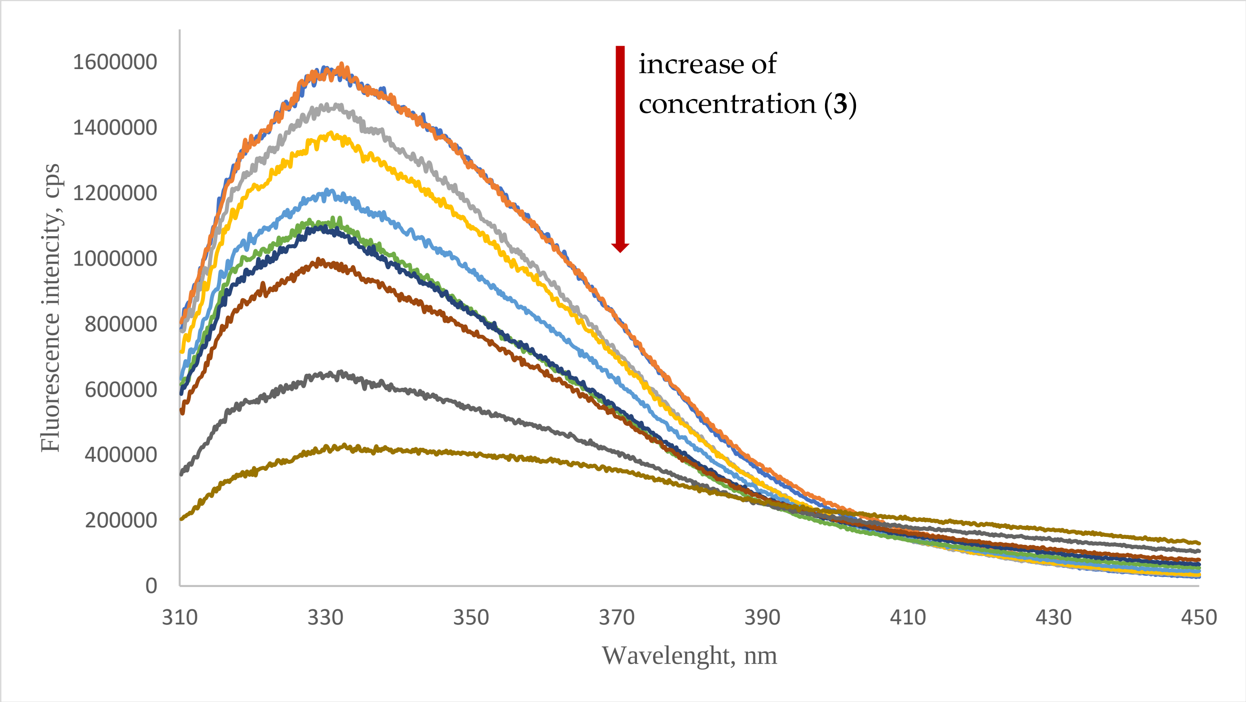

Figure S46. Fluorescence spectra of BSA in the presence of **3** at 20 °C.

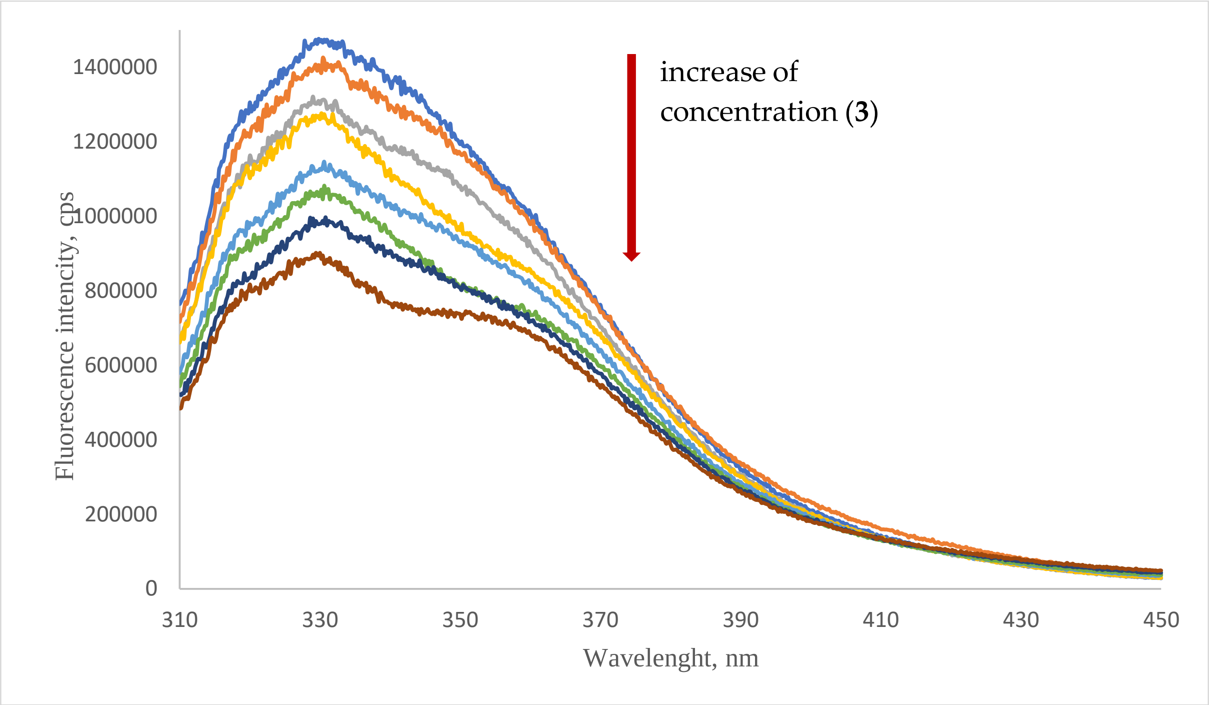

Figure S47. Fluorescence spectra of BSA in the presence of **3** at 35 °C.

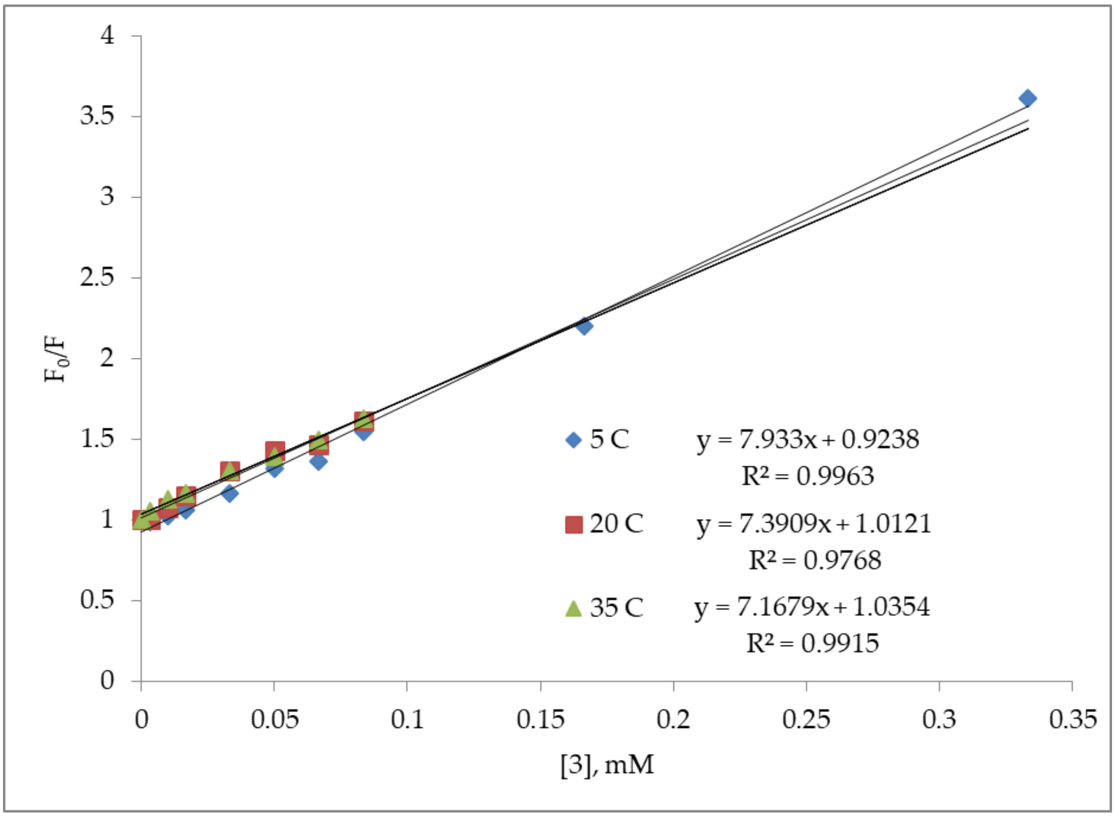

Figure S48. The graphs are plotted in the Stern-Volmer coordinates for **3**/BSA system.

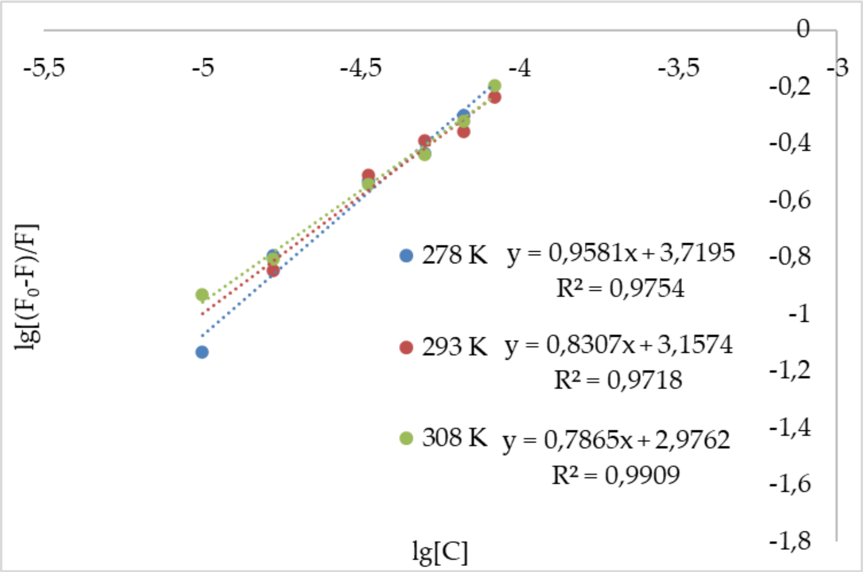

Figure S49. A plot of lg[(F_0–F)/F] vs. lg [C] for **3**/BSA system at different temperatures.

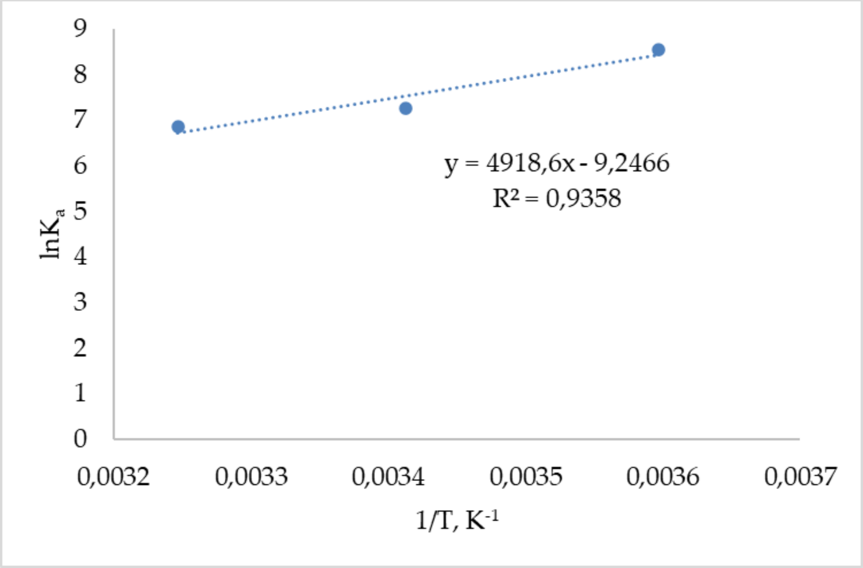

Figure S50. van’t Hof plot for **3**/BSA system at different temperatures.

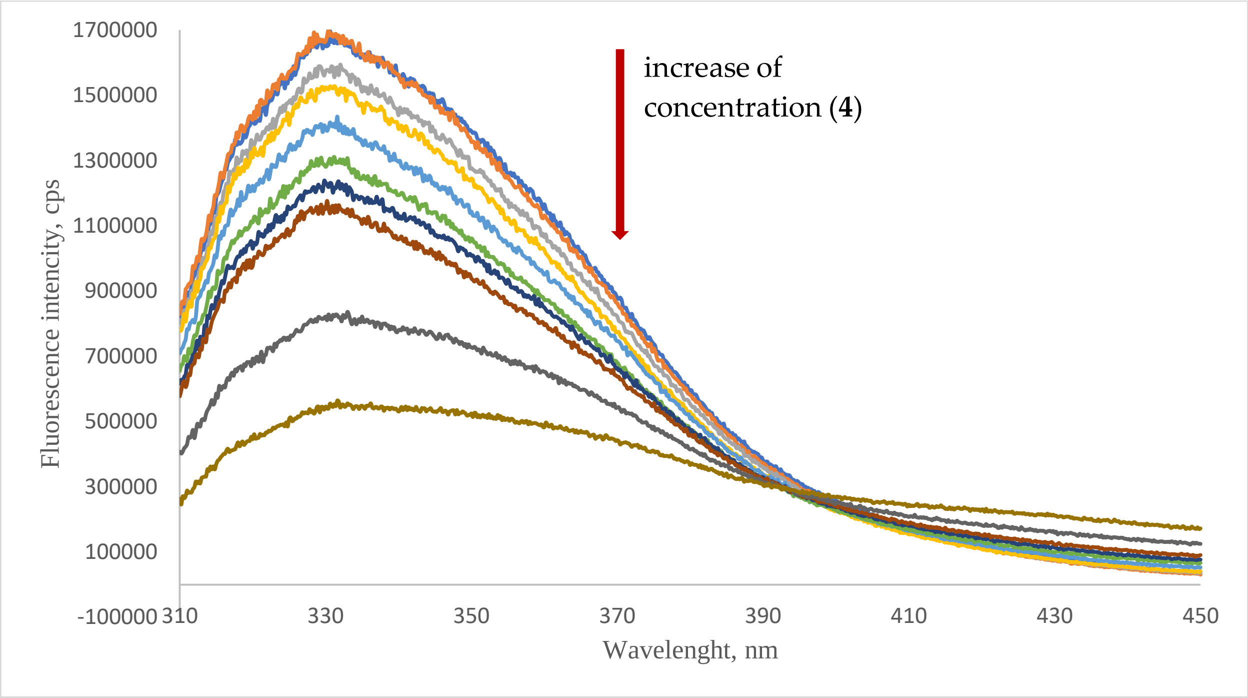

Figure S51. Fluorescence spectra of BSA in the presence of **4** at 5 °C.

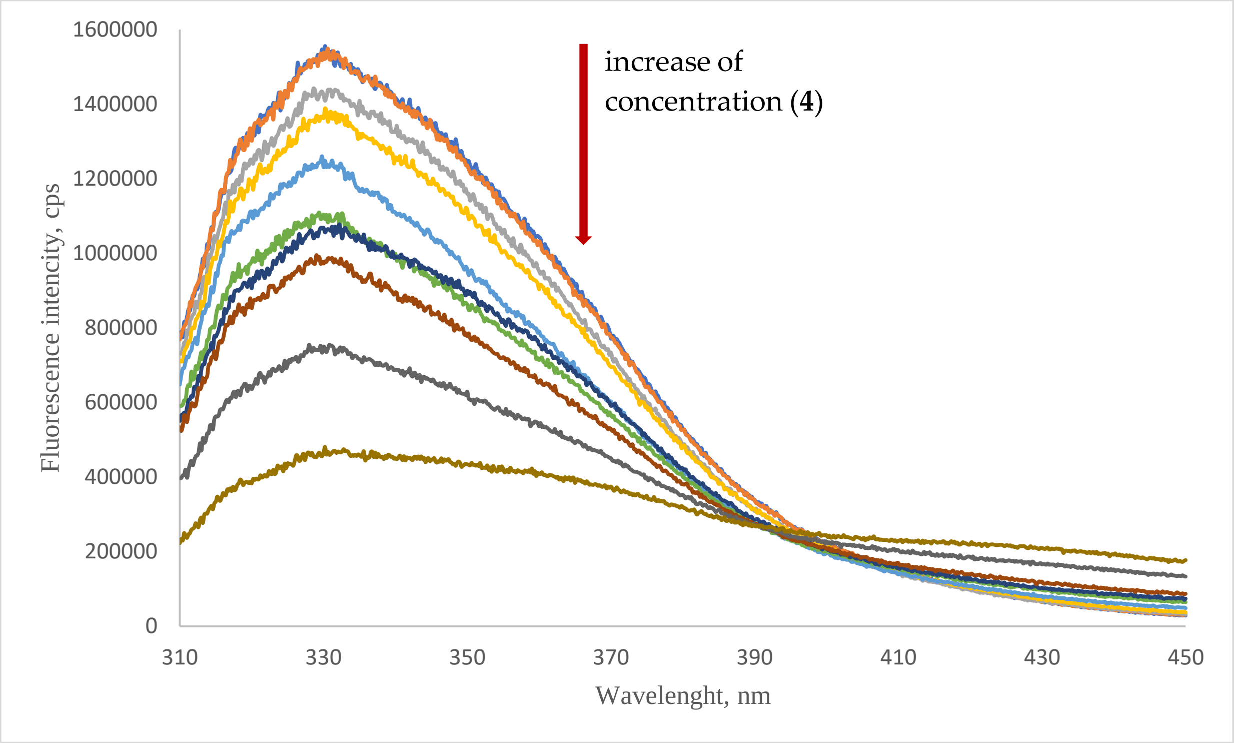

Figure S52. Fluorescence spectra of BSA in the presence of **4** at 20 °C.

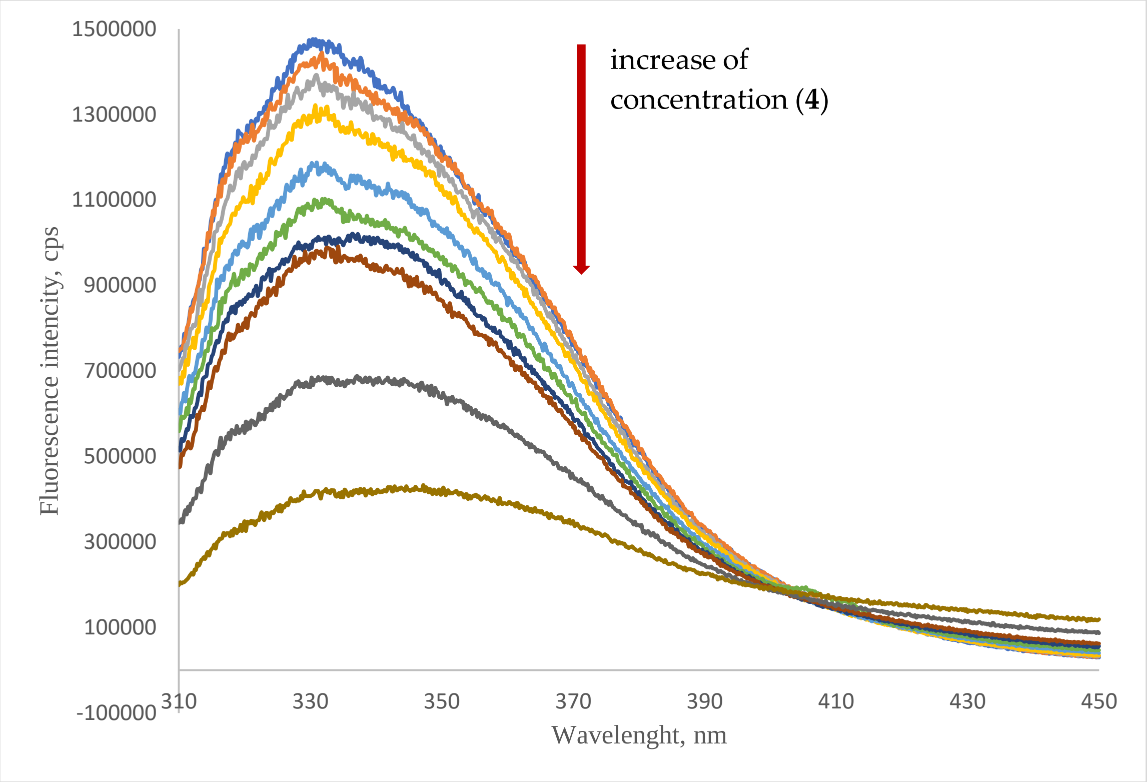

Figure S53. Fluorescence spectra of BSA in the presence of **4** at 35 °C.

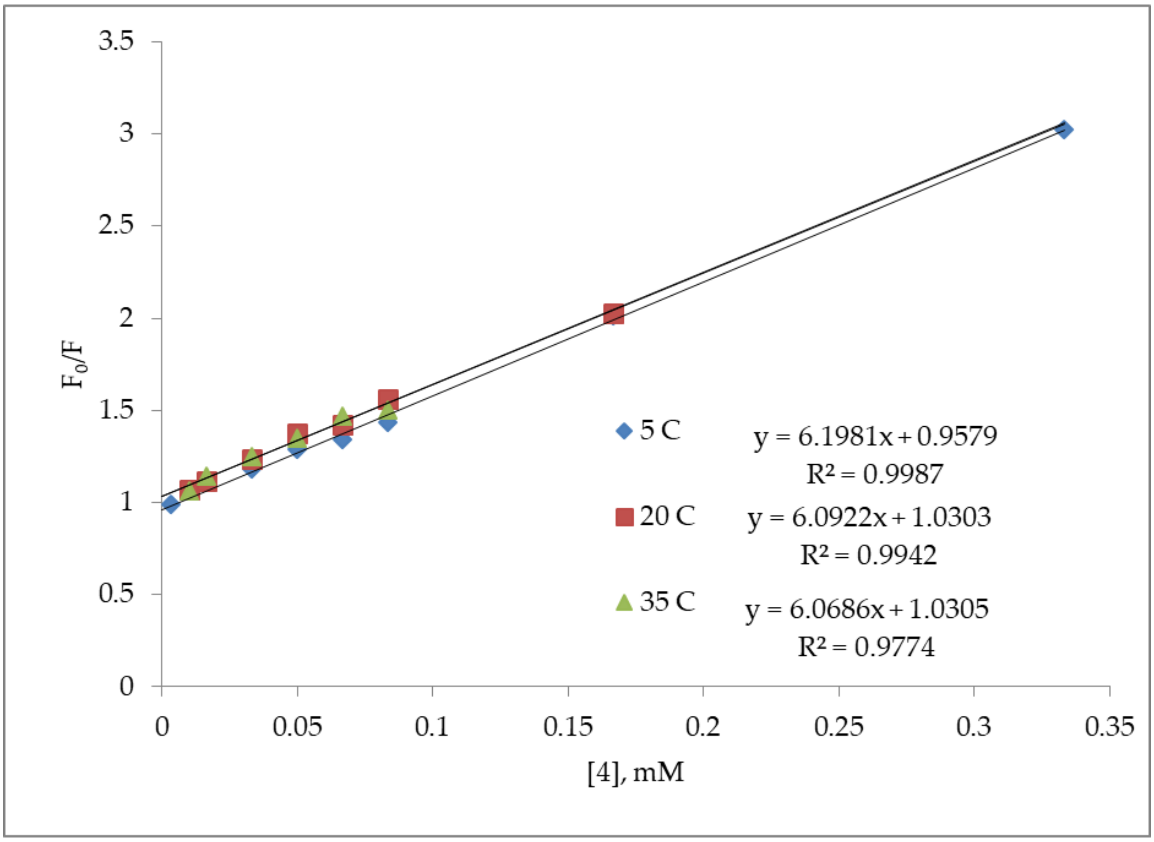

Figure S54. The graphs are plotted in the Stern-Volmer coordinates for **4**/BSA system.

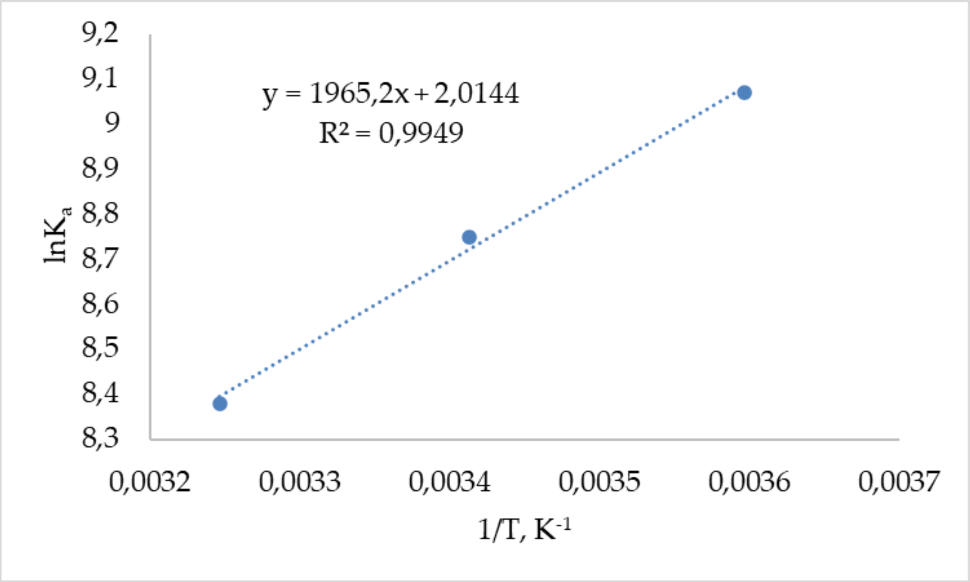

Figure S55. van’t Hof plot for **4**/BSA system at different temperatures.

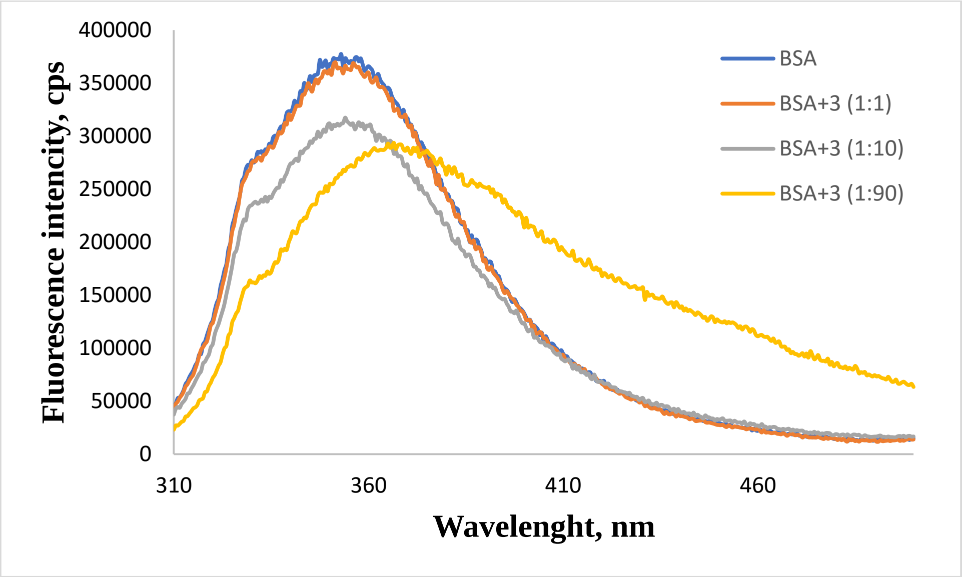

Figure S56. Fluorescence spectra of BSA (10^{-6} M) in the presence of **3** (excitation at 295 nm) in PBS.

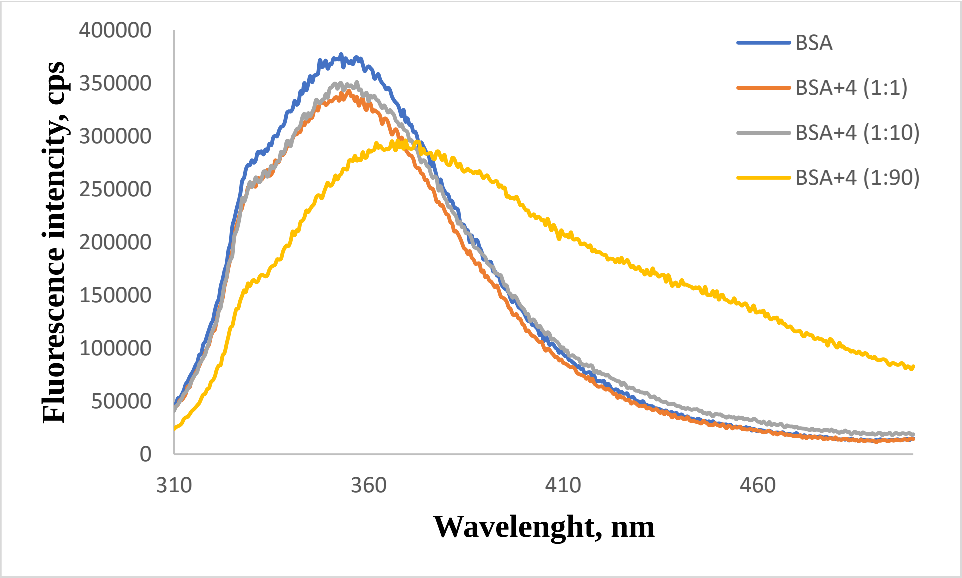

Figure S57. Fluorescence spectra of BSA (10^{-6} M) in the presence of **4** (excitation at 295 nm) in PBS.

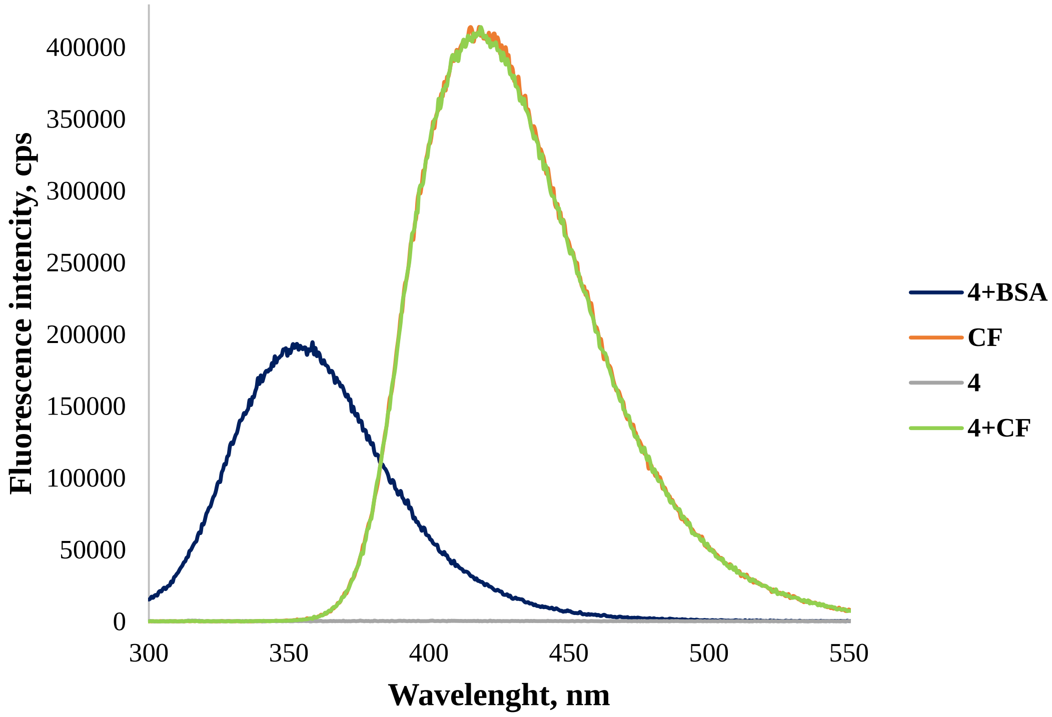

Figure S58. Fluorescence spectra of **4** (1×10^{-5} M), ciprofloxacin (4.17×10^{-5} M), **4**+BSA system (1/1, 1×10^{-5} M), **4**+ciprofloxacin system (1/1, 4.17×10^{-5} M).

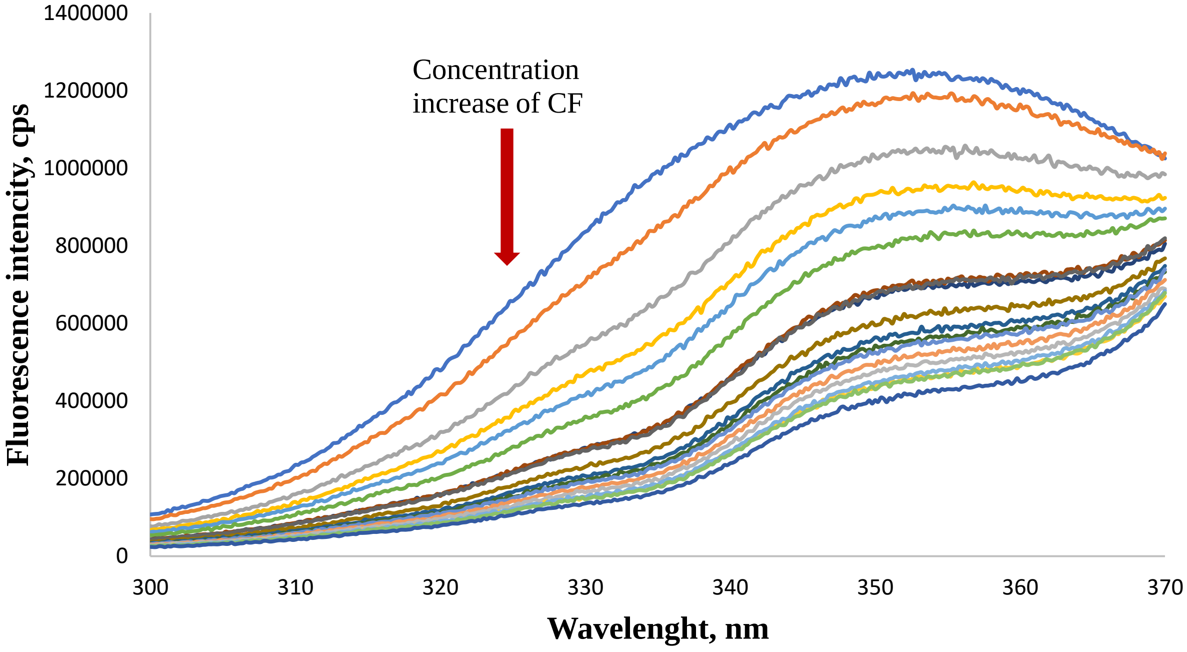

Figure S59. Fluorescence spectra of titration of BSA (1×10^{-5} M) with ciprofloxacin (concentration changed from 0 to 4×10^{-4} M).

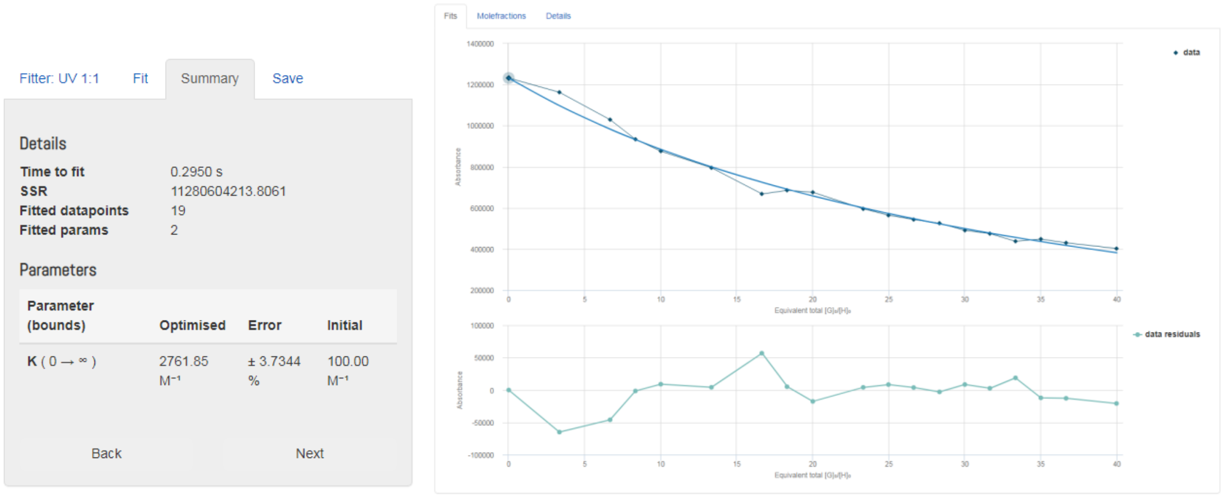

Figure S60. Bindfit (Fit data to 1:1 Host-Guest equilibria). Screenshots taken from the summary window of the website supramolecular.org. This screenshots shows the raw data for fluorescence titration of BSA with ciprofloxacin, the data fitted to 1:1 binding model.

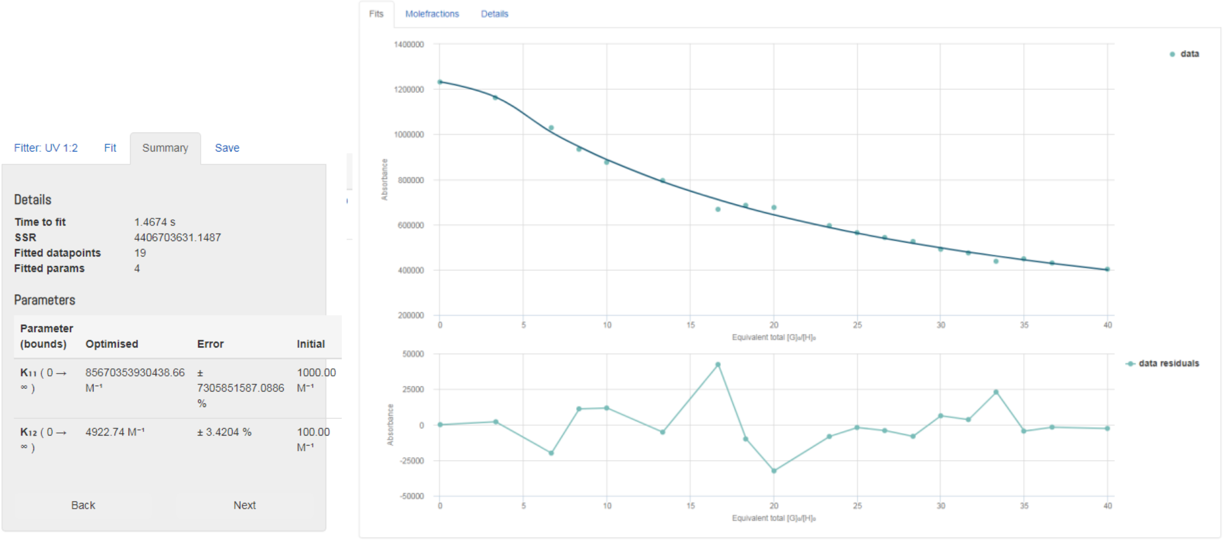

Figure S61. Bindfit (Fit data to 1:2 Host-Guest equilibria). Screenshots taken from the summary window of the website supramolecular.org. This screenshots shows the raw data for fluorescence titration of BSA with ciprofloxacin, the data fitted to 1:2 binding model.

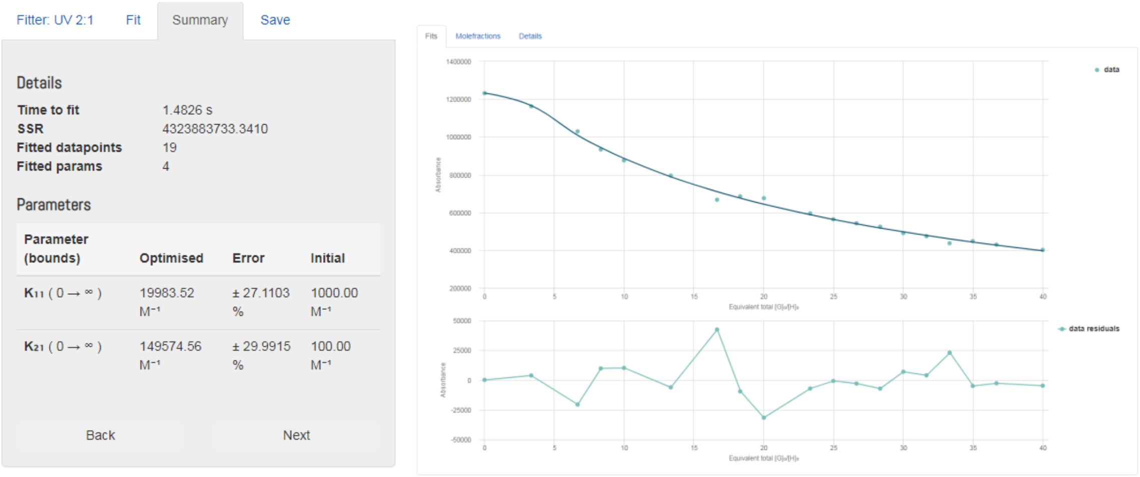

Figure S62. Bindfit (Fit data to 2:1 Host-Guest equilibria). Screenshots taken from the summary window of the website supramolecular.org. This screenshots shows the raw data for fluorescence titration of BSA with ciprofloxacin, the data fitted to 2:1 binding model.

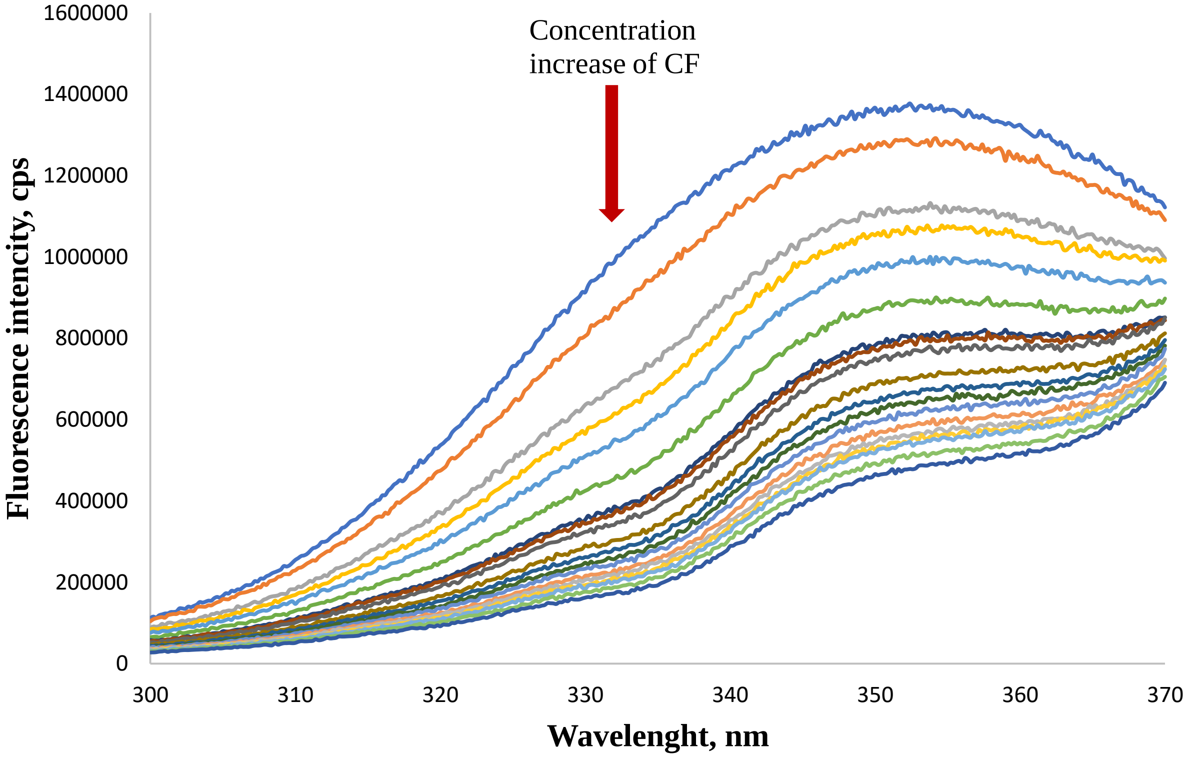

Figure S63. Fluorescence spectra of titration of **3** (1×10^{-5} M)/BSA (1×10^{-5} M) with ciprofloxacin (concentration changed from 0 to 4×10^{-4} M).

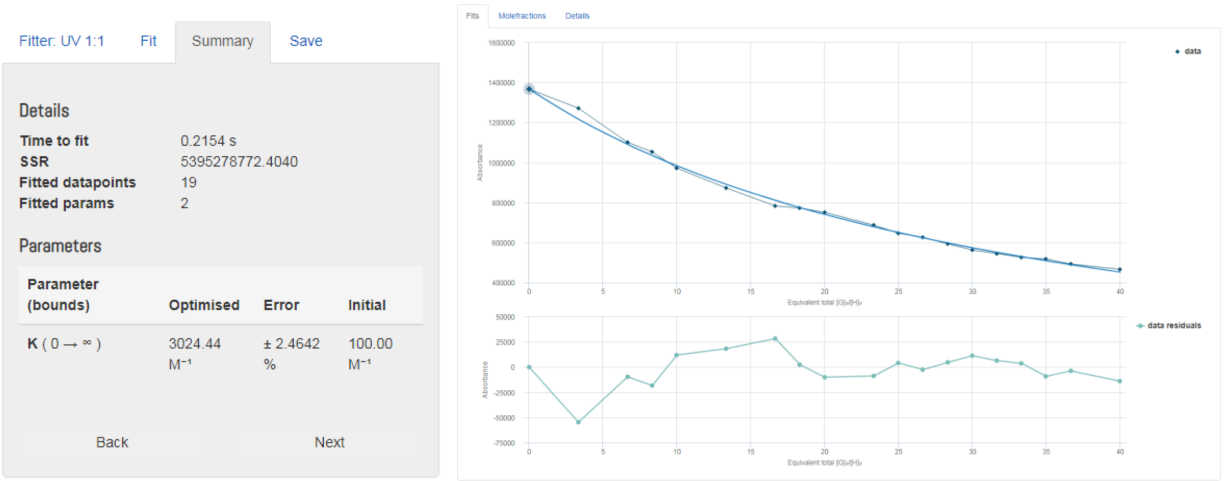

Figure S64. Bindfit (Fit data to 1:1 Host-Guest equilibria). Screenshots taken from the summary window of the website supramolecular.org. This screenshots shows the raw data for fluorescence titration of **3**/BSA (1:1) with ciprofloxacin, the data fitted to 1:1 binding model.

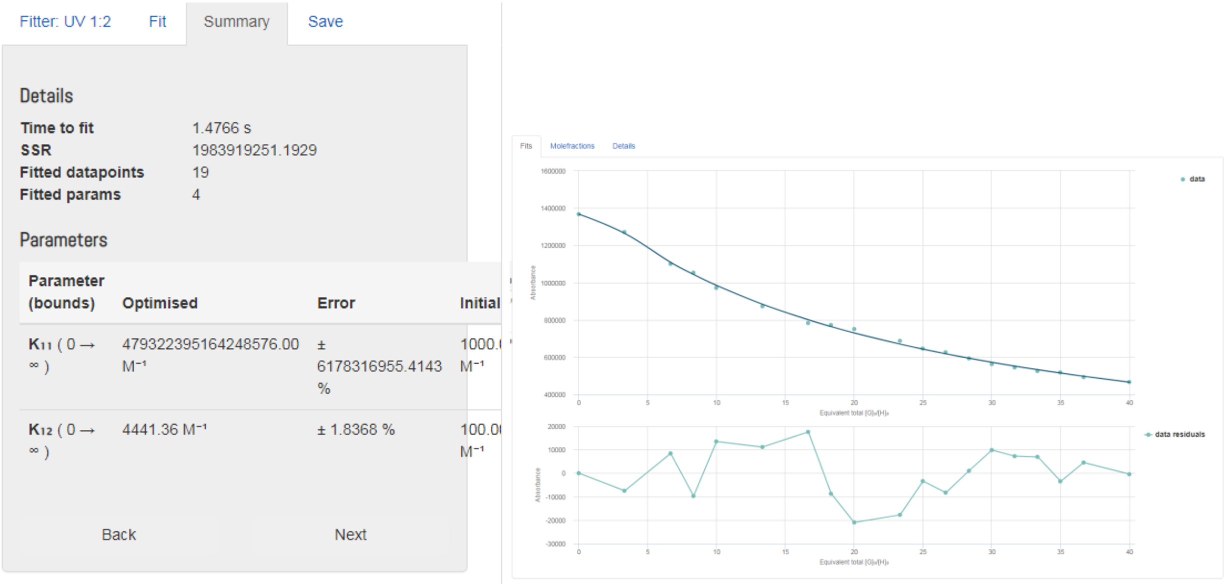

Figure S65. Bindfit (Fit data to 1:2 Host-Guest equilibria). Screenshots taken from the summary window of the website supramolecular.org. This screenshots shows the raw data for fluorescence titration of **3**/BSA (1:1) with ciprofloxacin, the data fitted to 1:2 binding model.

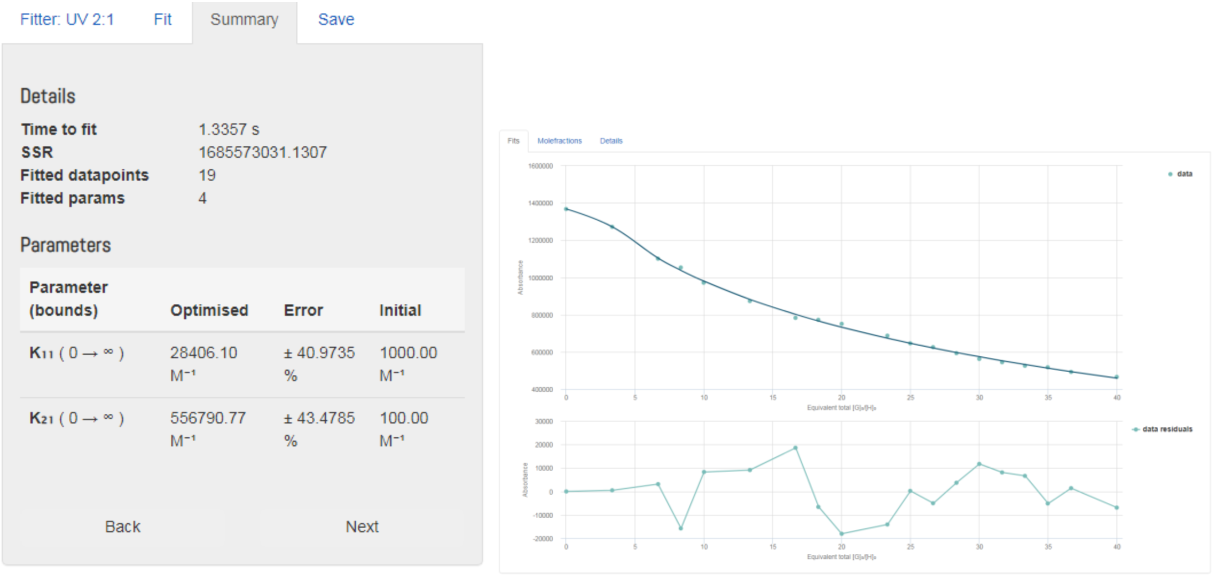

Figure S66. Bindfit (Fit data to 2:1 Host-Guest equilibria). Screenshots taken from the summary window of the website supramolecular.org. This screenshots shows the raw data for fluorescence titration of **3**/BSA (1:1) with ciprofloxacin, the data fitted to 2:1 binding model.

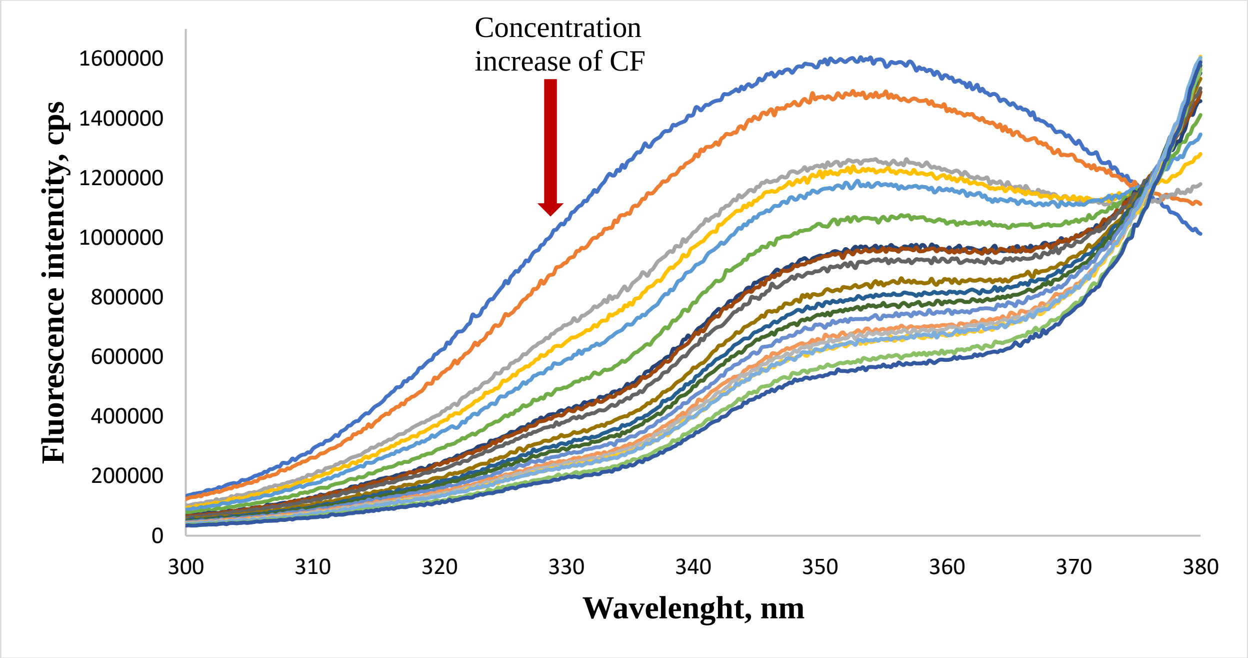

Figure S67. Fluorescence spectra of titration of **3** (1×10^{-5} M)/BSA (1×10^{-4} M) with ciprofloxacin (concentration changed from 0 to 4×10^{-4} M).

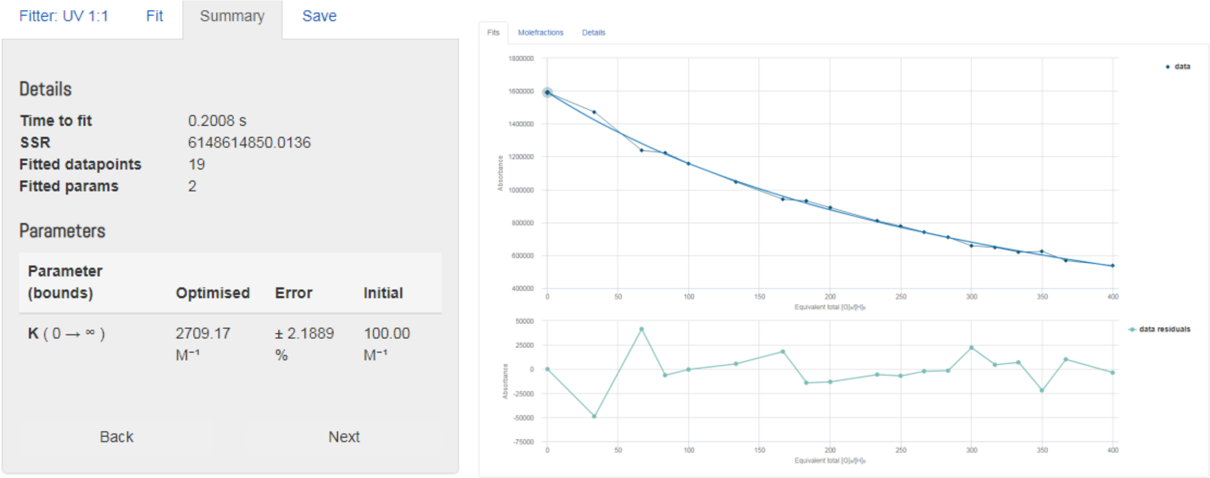

Figure S68. Bindfit (Fit data to 1:1 Host-Guest equilibria). Screenshots taken from the summary window of the website supramolecular.org. This screenshots shows the raw data for fluorescence titration of **3**/BSA (1:10) with ciprofloxacin, the data fitted to 1:1 binding model.

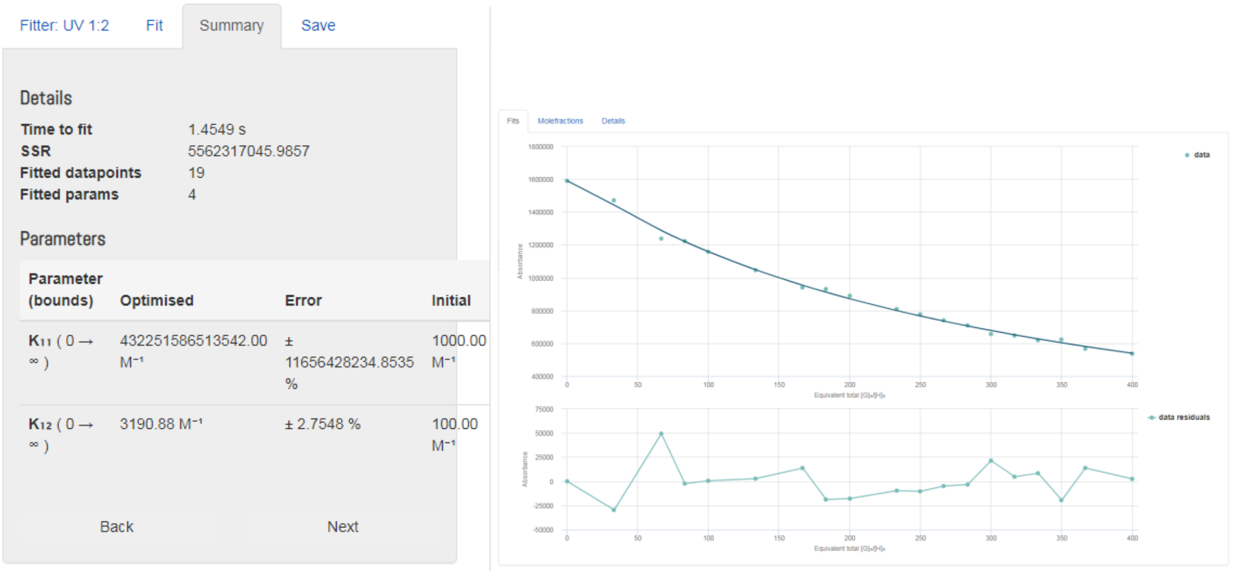

Figure S69. Bindfit (Fit data to 1:2 Host-Guest equilibria). Screenshots taken from the summary window of the website supramolecular.org. This screenshots shows the raw data for fluorescence titration of **3**/BSA (1:10) with ciprofloxacin, the data fitted to 1:2 binding model.

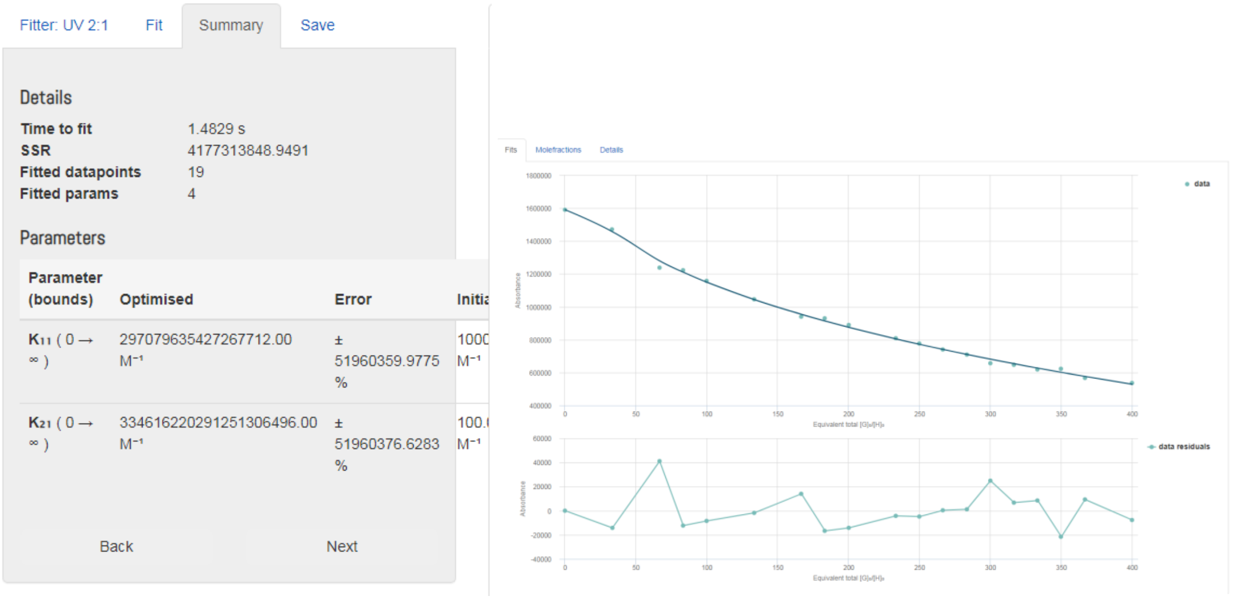

Figure S70. Bindfit (Fit data to 2:1 Host-Guest equilibria). Screenshots taken from the summary window of the website supramolecular.org. This screenshots shows the raw data for fluorescence titration of **3**/BSA (1:10) with ciprofloxacin, the data fitted to 2:1 binding model.

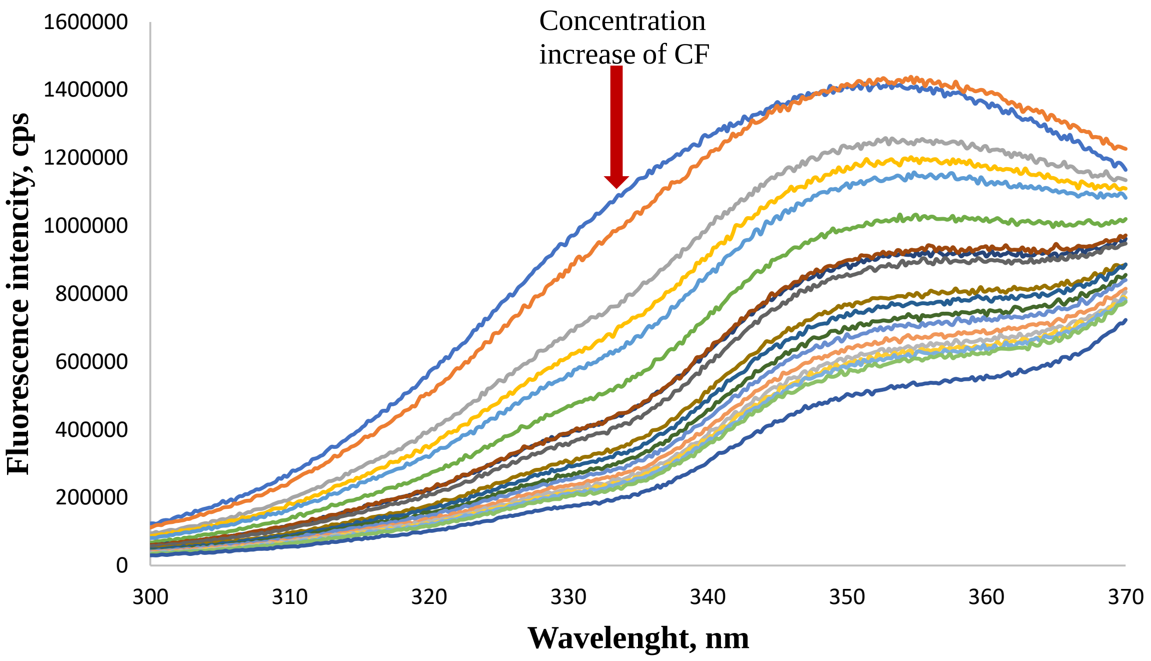

Figure S71. Fluorescence spectra of titration of **4** (1×10^{-5} M)/BSA (1×10^{-5} M) with ciprofloxacin (concentration changed from 0 to 4×10^{-4} M).

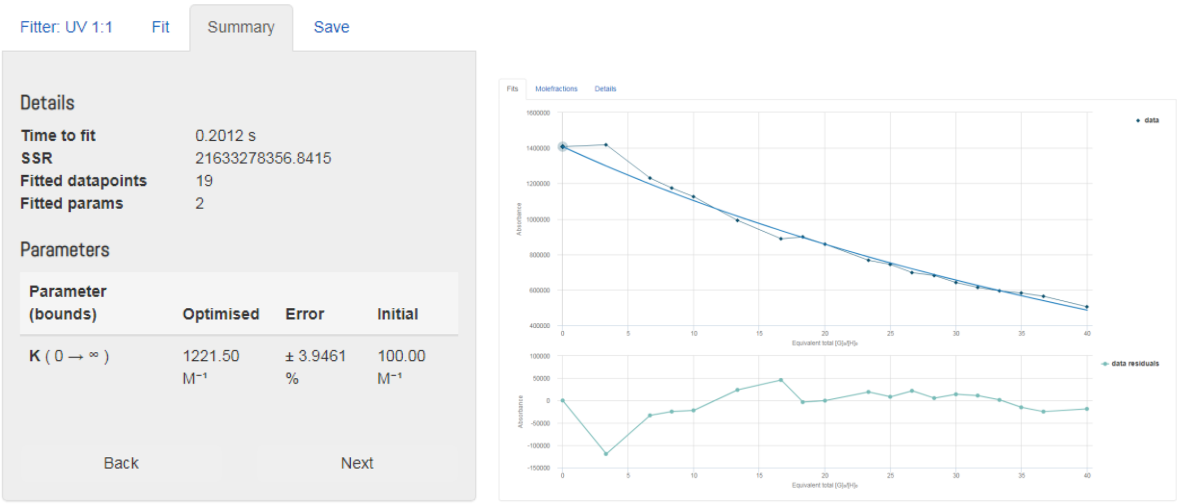

Figure S72. Bindfit (Fit data to 1:1 Host-Guest equilibria). Screenshots taken from the summary window of the website supramolecular.org. This screenshot shows the raw data for fluorescence titration of **4**/BSA (1:1) with ciprofloxacin, the data fitted to 1:1 binding model.

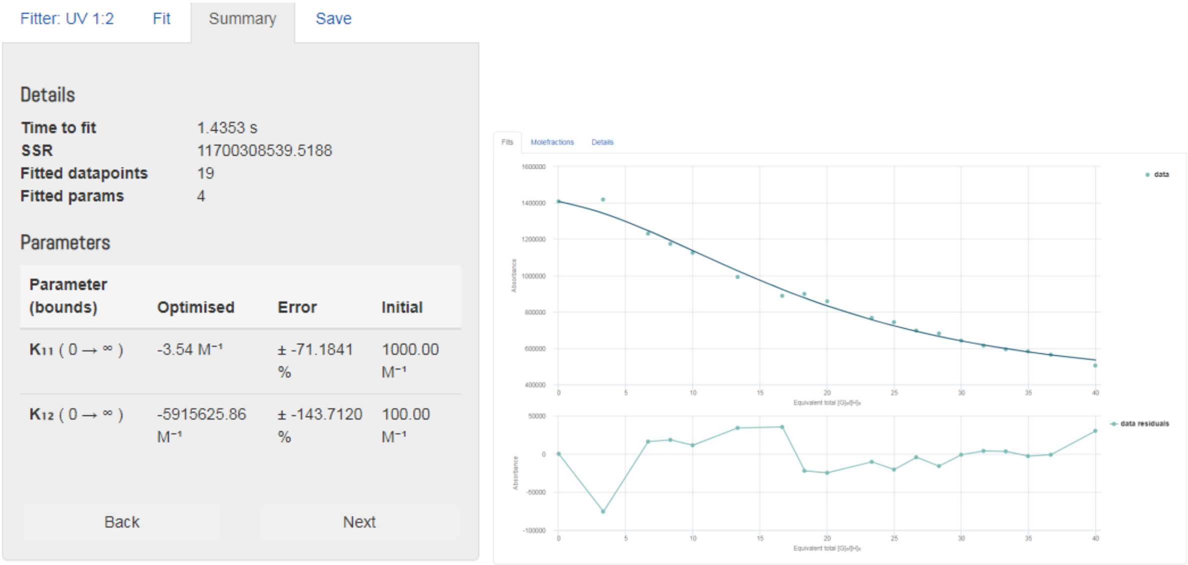

Figure S73. Bindfit (Fit data to 1:2 Host-Guest equilibria). Screenshots taken from the summary window of the website supramolecular.org. This screenshots shows the raw data for fluorescence titration of **4**/BSA (1:1) with ciprofloxacin, the data fitted to 1:2 binding model.

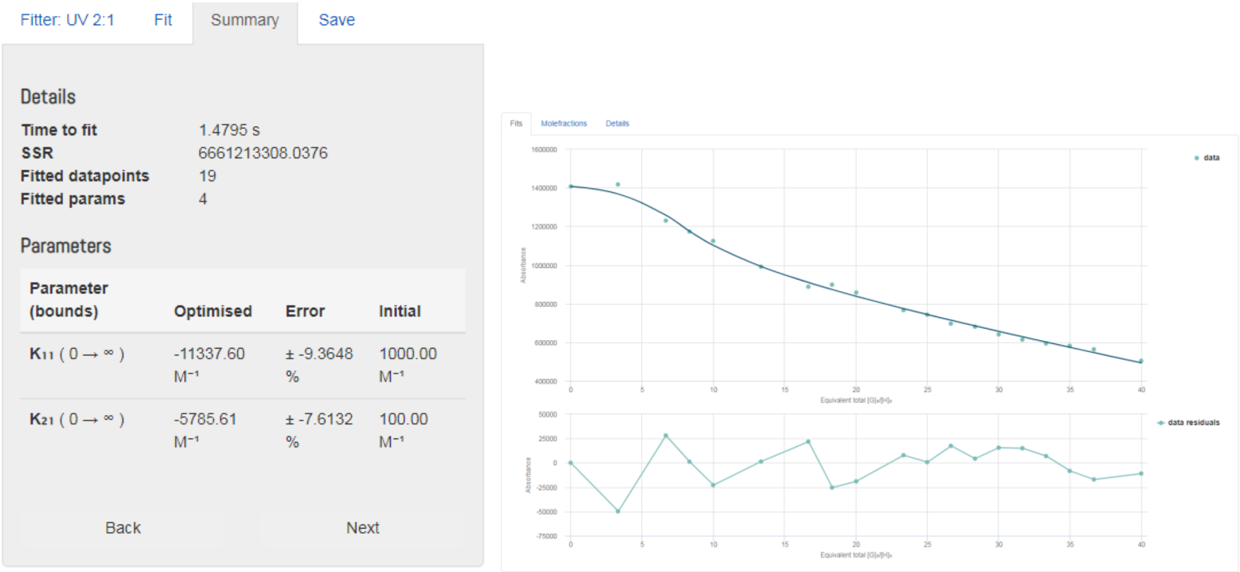

Figure S74. Bindfit (Fit data to 2:1 Host-Guest equilibria). Screenshots taken from the summary window of the website supramolecular.org. This screenshots shows the raw data for fluorescence titration of **4**/BSA (1:1) with ciprofloxacin, the data fitted to 2:1 binding model.

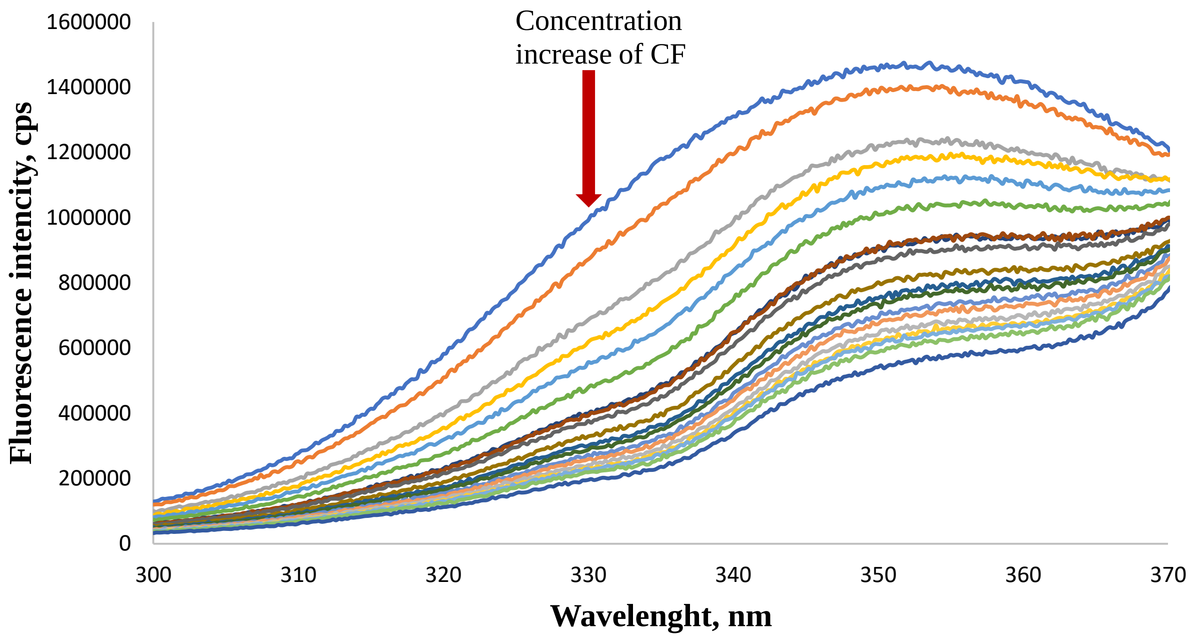

Figure S75. Fluorescence spectra of titration of **4** (1×10^{-5} M)/BSA (1×10^{-4} M) with ciprofloxacin (concentration changed from 0 to 4×10^{-4} M).

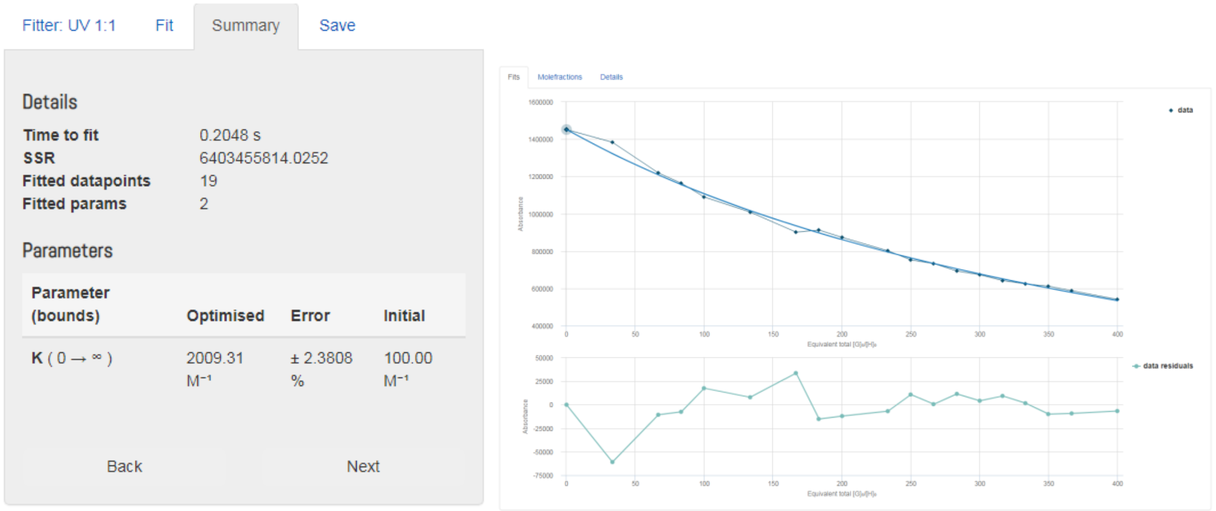

Figure S76. Bindfit (Fit data to 1:1 Host-Guest equilibria). Screenshots taken from the summary window of the website supramolecular.org. This screenshot shows the raw data for fluorescence titration of **4**/BSA (1:10) with ciprofloxacin, the data fitted to 1:1 binding model.

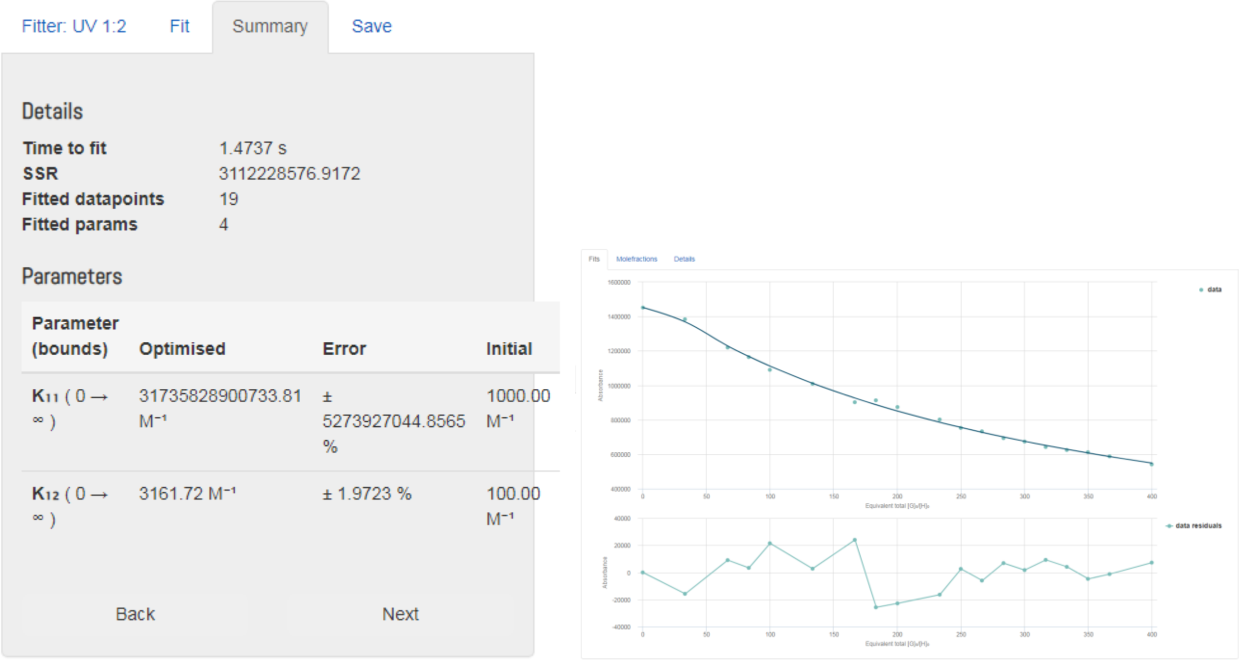

Figure S77. Bindfit (Fit data to 1:2 Host-Guest equilibria). Screenshots taken from the summary window of the website supramolecular.org. This screenshots shows the raw data for fluorescence titration of **4**/BSA (1:10) with ciprofloxacin, the data fitted to 1:2 binding model.

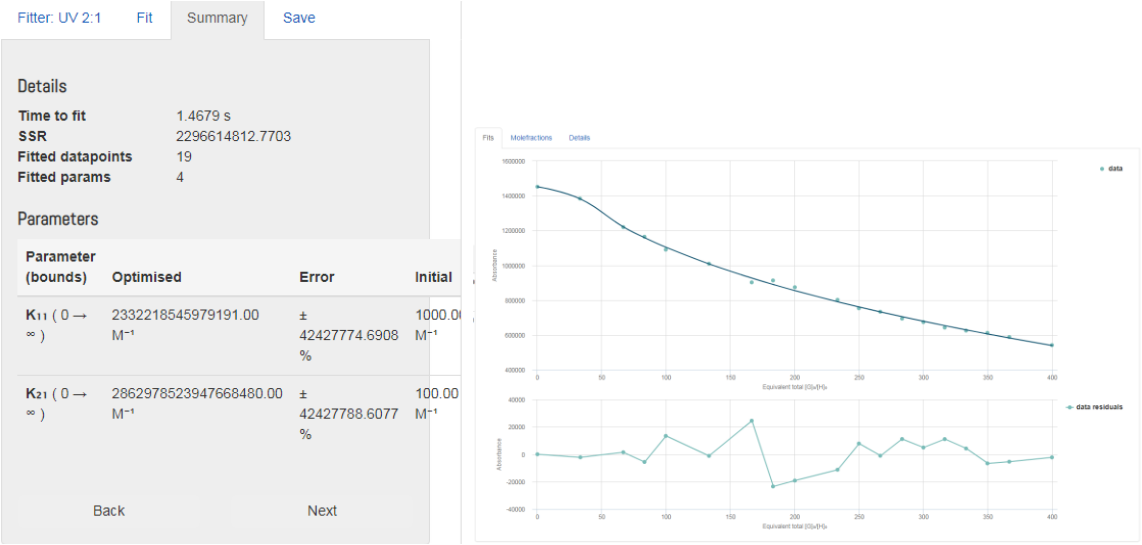

Figure S78. Bindfit (Fit data to 2:1 Host-Guest equilibria). Screenshots taken from the summary window of the website supramolecular.org. This screenshots shows the raw data for fluorescence titration of **4**/BSA (1:10) with ciprofloxacin, the data fitted to 2:1 binding model.

5. Tables

Table S1. Size of aggregates are formed during association of compounds **3** and **4** in phosphate buffer in the presence / absence of BSA (macrocycle/BSA 1:1).

System	C, M	D1, nm / S, %	D2, nm / S, %	D3, nm / S, %	PDI
BSA	5×10^{-5}	**7.0±0.07, 99%**	1521±2358, 1%		0.13±0.03
BSA	1×10^{-5}	**7.1±0.14, 97%**	3324±1883, 3%		0.17±0.05
BSA	5×10^{-6}	**6.9±0.16, 92%**	2712±1904, 7%	1864±2553, 1%	0.25±0.04
BSA	1×10^{-6}	7.0±0.43, 80%	1885±2217, 14%	3357±2287, 6%	0.18±0.04
**3**	5×10^{-5}	271±37, 92%	1136±2473, 6%	1098±2451, 2%	0.49±0.08
**3**	1×10^{-5}	343±4, 86%	3421±2937, 10%	1797±3099, 4%	0.45±0.06
**3**	5×10^{-6}	170±6, 94%	3212±2933, 6%		0.53±0.05
**3**	1×10^{-6}	325±27, 99%	2224±3045, 1%		0.56±0.09
**4**	5×10^{-5}	208±16, 95%	3352±2566, 4%	1685±2597, 1%	0.32±0.03
**4**	1×10^{-5}	186±18,100%			0.30±0.09
**4**	5×10^{-6}	187±20, 99%	2202±3016, 1%		0.27±0.06
**4**	1×10^{-6}	180±25, 93%	2344±2706, 7%		0.54±0.16
**3**/BSA	$5\times10^{-5}/5\times10^{-5}$	**7.2±0.15, 98%**	3748±1655, 2%		0.15±0.02
**3**/BSA	$1\times10^{-5}/1\times10^{-5}$	**7.0±0.22, 94%**	3386±1533, 4%	1184±2054, 2%	0.22±0.04
**3**/BSA	$5\times10^{-6}/5\times10^{-6}$	**7.1±0.11, 89%**	1157±238, 10%	2185±2552, 1%	0.26±0.02
**3**/BSA	$1\times10^{-6}/1\times10^{-6}$	6.8±0.21, 81%	462±266, 14%	4607±436, 5%	0.26±0.05
**4**/BSA	$5\times10^{-5}/5\times10^{-5}$	**6.8±0.05, 76%**	281±14, 24%		0.38±0.01
**4**/BSA	$1\times10^{-5}/1\times10^{-5}$	**7.0±0.13, 72%**	308±32, 25%	3692±2095, 3%	0.43±0.03
**4**/BSA	$5\times10^{-6}/5\times10^{-6}$	**7.0±0.02, 80%**	300±12, 20%		0.35±0.02
**4**/BSA	$1\times10^{-6}/1\times10^{-6}$	**6.9±0.10, 72%**	264±42, 24%	4592±2402, 4%	0.28±0.10

Table S2. Size of aggregates are formed during association of compounds **3** and **4** in phosphate buffer in the presence / absence of BSA (macrocycle/BSA 1:10).

System	C, M	D1, nm / S, %	D2, nm / S, %	D3, nm / S, %	PDI
BSA	5×10^{-4}	**6.2±0.09, 93%**	3391±926, 7%		0.22±0.02
BSA	1×10^{-4}	**6.9±0.04, 99%**	1887±2585, 1%		0.14±0.02
BSA	5×10^{-5}	**7.0±0.07, 99%**	1521±2358, 1%		0.13±0.03

BSA	1×10^{-5}	**7.1±0.14, 97%**	3324±1883, 3%		0.17±0.05
BSA	5×10^{-6}	**6.9±0.16, 92%**	2712±1904, 7%	1864±2553, 1%	0.25±0.04
BSA	1×10^{-6}	7.0±0.43, 80%	1885±2217, 14%	3357±2287, 6%	0.18±0.04
**3**	5×10^{-5}	271±37, 92%	1136±2473, 6%	1098±2451, 2%	0.49±0.08
**3**	1×10^{-5}	343±4, 86%	3421±2937, 10%	1797±3099, 4%	0.45±0.06
**3**	5×10^{-6}	170±6, 94%	3212±2933, 6%		0.53±0.05
**3**	1×10^{-6}	325±27, 99%	2224±3045, 1%		0.56±0.09
**4**	5×10^{-5}	208±16, 95%	3352±2566, 4%	1685±2597, 1%	0.32±0.03
**4**	1×10^{-5}	186±18,100%			0.30±0.09
**4**	5×10^{-6}	187±20, 99%	2202±3016, 1%		0.27±0.06
**4**	1×10^{-6}	180±25, 93%	2344±2706, 7%		0.54±0.16
**3**/BSA	5×10^{-5}/5×10^{-4}	**6.0±0.03, 75%**	623±100, 23%	2334±2695, 2%	0.38±0.01
**3**/BSA	1×10^{-5}/1×10^{-4}	**6.6±0.06, 84%**	710±95, 16%		0.31±0.02
**3**/BSA	5×10^{-6}/5×10^{-5}	**6.6±0.16, 83%**±	612±140, 16%	1981±2714, 1%	0.30±0.03
**3**/BSA	1×10^{-6}/1×10^{-5}	**6.8±0.05, 85%**±	667±94, 14%	2305±2662	0.30±0.01
**4**/BSA	5×10^{-5}/5×10^{-4}	**6.1±0.14, 93%**	2661±1274, 6%	1159±1975	0.22±0.03
**4**/BSA	1×10^{-5}/1×10^{-4}	**7.0±0.07, 99%**	2856±2216, 1%		0.15±0.02
**4**/BSA	5×10^{-6}/5×10^{-5}	**7.2±0.07, 99%**	3640±1784, 1%		0.15±0.01
**4**/BSA	1×10^{-6}/1×10^{-5}	**7.1±0.10, 91%**	1652±759, 8%	1417±2196, 1%	0.25±0.03
